# GDF8 inhibition enhances musculoskeletal recovery and mitigates posttraumatic osteoarthritis following joint injury

**DOI:** 10.1126/sciadv.adi9134

**Published:** 2023-11-29

**Authors:** Camille R. Brightwell, Christine M. Latham, Alexander R. Keeble, Nicholas T. Thomas, Allison M. Owen, Kelsey A. Reeves, Douglas E. Long, Matthew Patrick, Sara Gonzalez-Velez, Varag Abed, Ramkumar T. Annamalai, Cale Jacobs, Caitlin E. Conley, Gregory S. Hawk, Austin V. Stone, Jean L. Fry, Katherine L. Thompson, Darren L. Johnson, Brian Noehren, Christopher S. Fry

**Affiliations:** ^1^ Center for Muscle Biology, University of Kentucky, Lexington, KY, USA.; ^2^ Department of Athletic Training and Clinical Nutrition, College of Health Sciences, University of Kentucky, Lexington, KY, USA.; ^3^ Department of Physical Therapy, College of Health Sciences, University of Kentucky, Lexington, KY, USA.; ^4^ Department of Biomedical Engineering, College of Engineering, University of Kentucky, Lexington, KY, USA.; ^5^ Department of Orthopaedic Surgery and Sports Medicine, College of Medicine, University of Kentucky, Lexington, KY, USA.; ^6^ Department of Statistics, College of Arts and Sciences, University of Kentucky, Lexington, KY, USA.

## Abstract

Musculoskeletal disorders contribute substantially to worldwide disability. Anterior cruciate ligament (ACL) tears result in unresolved muscle weakness and posttraumatic osteoarthritis (PTOA). Growth differentiation factor 8 (GDF8) has been implicated in the pathogenesis of musculoskeletal degeneration following ACL injury. We investigated GDF8 levels in ACL-injured human skeletal muscle and serum and tested a humanized monoclonal GDF8 antibody against a placebo in a mouse model of PTOA (surgically induced ACL tear). In patients, muscle GDF8 was predictive of atrophy, weakness, and periarticular bone loss 6 months following surgical ACL reconstruction. In mice, GDF8 antibody administration substantially mitigated muscle atrophy, weakness, and fibrosis. GDF8 antibody treatment rescued the skeletal muscle and articular cartilage transcriptomic response to ACL injury and attenuated PTOA severity and deficits in periarticular bone microarchitecture. Furthermore, GDF8 genetic deletion neutralized musculoskeletal deficits in response to ACL injury. Our findings support an opportunity for rapid targeting of GDF8 to enhance functional musculoskeletal recovery and mitigate the severity of PTOA after injury.

## Teaser

Blockade of growth differentiation factor 8 abates progressive musculoskeletal degeneration in posttraumatic osteoarthritis.

## INTRODUCTION

Musculoskeletal disorders contribute to a substantial proportion of disability among people worldwide (*1*, *2*). Knee osteoarthritis (OA) is an exceptionally prevalent chronic disease that is characterized by the progressive destruction of cartilage, causing pain and disability (*3*, *4*). Prior joint injury, such as that sustained during anterior cruciate ligament (ACL) tear, markedly predisposes the development of posttraumatic osteoarthritis (PTOA). More than 200,000 ACL tears occur annually in the United States (*5*, *6*), with 50 to 90% of individuals going on to develop PTOA within 15 years (*7*, *8*). There are currently no treatments to address the persistent weakness and joint degeneration in PTOA, but the development of interventions given at the time of ligament injury offers an opportunity for prevention of PTOA.

Growth differentiation factor 8 (GDF8), or myostatin, is a myokine that has been implicated in the pathogenesis of musculoskeletal degeneration in knee OA and following ACL injury (*9*–*11*). As a member of the transforming growth factor–β superfamily, GDF8 is most known for its role as a negative regulator of muscle size (*12*, *13*). GDF8 activates SMAD2/3 signaling to affect transcriptional activity, where, in addition to promoting muscle atrophy (*13*), it can inhibit osteogenic differentiation (*14*, *15*) and chondrocyte proliferation (*16*). Preclinical work from our group and others has also revealed that GDF8 directly regulates skeletal muscle fibrosis (*9*, *17*). Given its potent musculoskeletal growth antagonism, strategies to inhibit GDF8 have been thoroughly investigated. Genetic deletion strategies and soluble receptor/neutralizing antibodies have shown efficacy in attenuating musculoskeletal loss during cancer cachexia (*18*, *19*) and spaceflight (*20*), and several GDF8-targeting pharmaceutical products are currently in clinical development (*21*–*24*).

We hypothesized that early blockade of GDF8 via humanized monoclonal antibody following ACL injury would protect against rapid deficits in muscle strength and size and attenuate fibrosis development—in addition to mitigating longer-term loss of periarticular bone microarchitecture and the development of PTOA. Here, we describe in human participants the rapid increase in GDF8 following ACL injury that predicts muscle atrophy, weakness, and periarticular bone loss in patients 6 months following surgical reconstruction. Using mouse models of PTOA, we demonstrate that genetic and antibody blockade of GDF8 restored muscle size and functionality and reduced the induction of metalloproteinases within articular cartilage. GDF8 inhibition reduced muscle fibrosis, rescued the skeletal muscle and articular cartilage transcriptomic response to ACL injury, and attenuated PTOA severity. Together, these data demonstrate that GDF8 represents an early modifiable target in human joint injury, and anti-GDF8 antibody (anti-GDF8 Ab) administration is an effective countermeasure to musculoskeletal perturbations following joint injury.

## RESULTS

### Early induction of GDF8 in skeletal muscle is predictive of prolonged musculoskeletal deficits following a ligament injury and surgical reconstruction

**Fig. 1.**
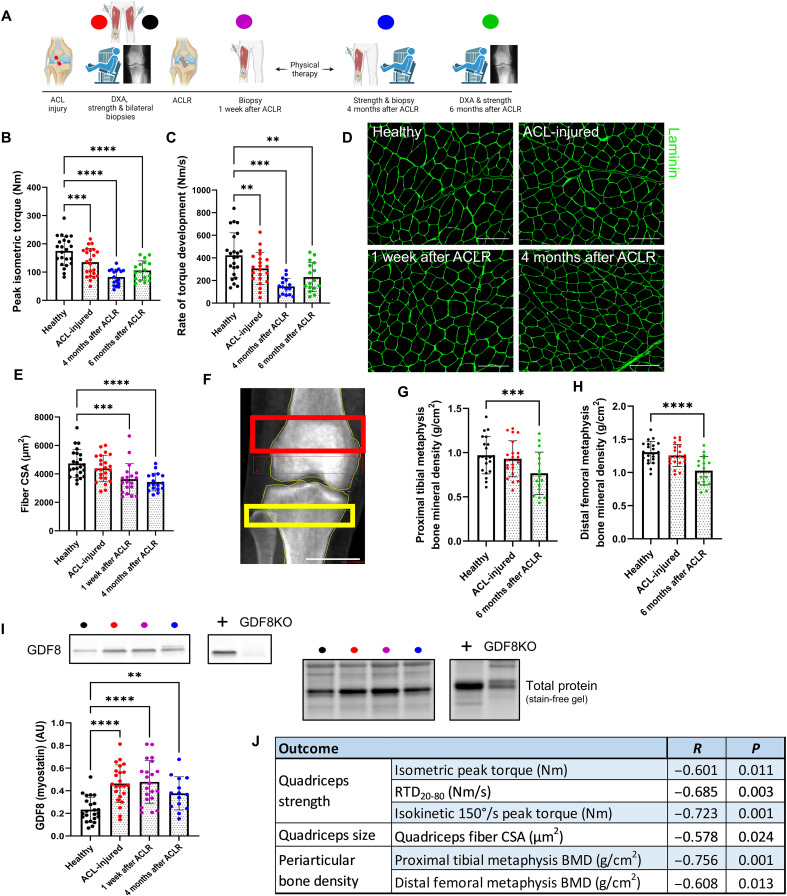
GDF8 is a molecular transducer of musculoskeletal deficits that are obstinate to surgical reconstruction and rehabilitation following ligamentous injury. (**A**) Participants with ACL injury underwent measures of knee extensor dynamometry, quadriceps biopsies, and periarticular bone mineral density at indicated time points pre- and post-reconstruction. (**B**) Knee extension peak isometric torque and (**C**) rate of torque development are reduced after ACL injury and do not recover. (**D**) Representative immunohistochemistry (IHC) image of muscle fiber cross-sectional area (CSA). Scale bars, 200 μm. (**E**) Quadriceps muscle fiber atrophy occurs progressively after ACL injury and does not recover. (**F**) Representative image from dual-energy x-ray absorptiometry (DXA). The red box indicates distal femoral metaphysis; the yellow box indicates proximal tibial metaphysis. Scale bar, 4 cm. Bone mineral density is decreased in the (**G**) proximal tibial metaphysis and (**H**) distal femoral metaphysis 6 months following ACLR. (**I**) GDF8/myostatin expression is elevated in the quadriceps muscle after ACL injury and remains elevated after ACL surgical reconstruction (ACLR). (**J**) Greater loss of quadriceps/knee extension strength, quadriceps size, and periarticular bone density at 4 to 6 months after reconstruction are correlated with quadriceps muscle GDF8 expression after ACL injury. Scatterplots can be seen in fig. S3. *N* = 23 (B to E and I), 21 (G and H). **P < 0.01, ***P < 0.005, and ****P < 0.001 versus healthy via mixed effects model and Dunnett’s correction for multiple comparisons. AU, arbitrary units; GDF8KO, GDF8 knockout; BMD, bone mineral density; RTD, rate of torque development.

Across our patient cohort (table S1), quadriceps strength and ability to rapidly generate torque (rate of torque development) were depressed immediately following injury to the ACL and failed to recover following surgical reconstruction and physical therapy (Fig. 1, A to C, and fig. S1A). Strength loss was accompanied by quadriceps muscle fiber atrophy [25% reduction in fiber cross-sectional area (CSA); Fig. 1, D and E] and collagen accumulation (fig. S1B) in the injured limb. Quadriceps myocellular deficits (atrophy and fibrosis) following ACL injury are unresponsive to the current standard of care (*9*, *25*) and persist in patients with knee OA (*26*, *27*). As a consequence of quadriceps atrophy and weakness, ACL injury alters joint mechanics, disrupting the physiological loading of periarticular bone and cartilage, contributing to PTOA pathology (*28*). Notably, subchondral bone remodeling precedes cartilage destruction in PTOA (*29*). Bone mineral density (BMD) of the proximal tibial metaphysis and distal femoral metaphysis declined by ~20% 6 months following surgical reconstruction in our cohort (Fig. 1, F to H). The initial ACL rupture induced a rapid >2-fold elevation in GDF8 protein abundance in the injured limb quadriceps that remained elevated at 1 week and 4 months after surgical reconstruction (Fig. 1I). Immunoblot detection of GDF8 was validated in a subset of participants via enzyme-linked immunosorbent assay (fig. S1C). Serum concentration of GDF8 was highest before surgical reconstruction (fig. S1D), and quadriceps GDF8 levels were positively correlated to serum GDF8 concentration before reconstruction (*R* = 0.465; fig. S1E). The mature GDF8 protein dimerizes before binding the activin receptor type IIB (ActRIIB) and recruiting anaplastic lymphoma kinase 4/5 to activate the SMAD2/3 signaling pathway to affect the target gene transcriptional activity (*30*). SMAD3 phosphorylation (Ser^423/425^) is elevated in whole quadriceps homogenates and myonuclei following initial ligament injury and 1 week following surgical reconstruction (figs. S1, F to H, and S2, A and B). Notably, participants who had the largest induction in quadriceps GDF8 following ACL injury showed the most severe performance and musculoskeletal deficits (strength, atrophy, and periarticular bone loss) 4 to 6 months following reconstruction (Pearson’s correlations in Fig. 1J and scatterplots in fig. S3). Quadriceps atrophy and weakness, as well as periarticular bone loss, are normalized to the noninvolved contralateral limb. Measures of muscle strength and BMD within the “healthy” noninvolved contralateral limb showed no meaningful change across 6 months of follow-up, supporting its utility as an internal genetic control (fig. S4).

### Humanized monoclonal antibody targeting GDF8 reduces phospho-SMAD3 and prevents quadriceps atrophy and weakness following ACL injury

**Fig. 2.**
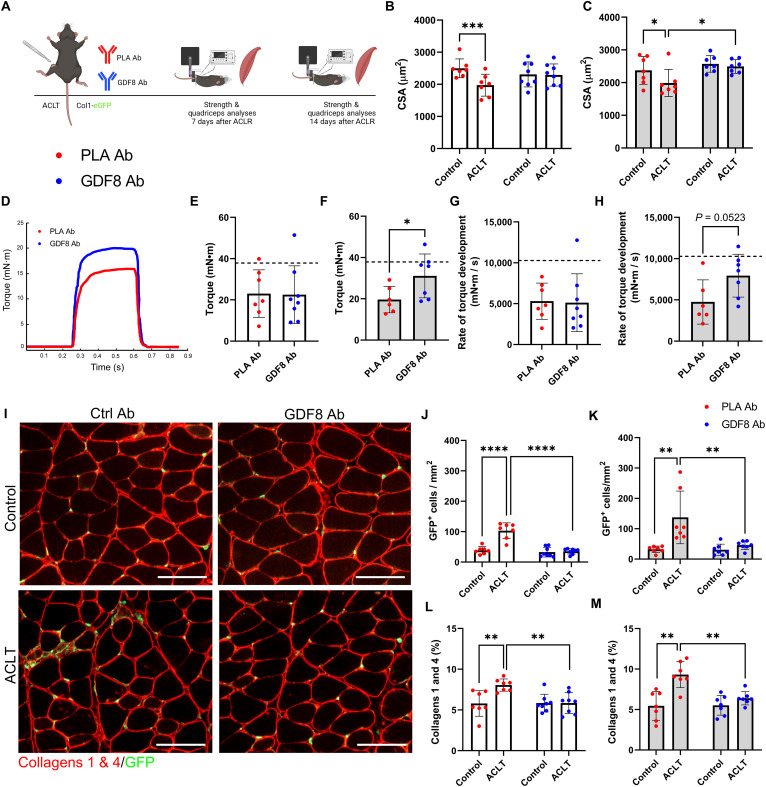
Humanized monoclonal antibody targeting GDF8 mitigates quadriceps atrophy and weakness and improves muscle quality following ACL injury. (**A**) Study diagram; mice were treated biweekly. (**B**) PLA Ab–treated mice show quadriceps atrophy 1-week after ACL transection (ACLT) (drug × injury interaction, *P* = 0.002). (**C**) GDF8 Ab treatment rescues quadriceps fiber atrophy at 2 weeks after ACLT (drug × injury interaction, *P* = 0.024). (**D**) Representative tetanic graph from quadriceps peak isometric torque. (**E**) PLA Ab– and GDF8 Ab–treated mice show similar knee extension torque at 1 week after ACLT. (**F**) Knee extension weakness is mitigated by GDF8 Ab treatment 2 weeks after ACLT. Dashed lines in (E) and (F) represent mean values from uninjured control mice from historical laboratory data. (**G**) PLA Ab– and GDF8 Ab–treated mice show similar knee extension rates of torque development at 1 week after ACLT. (**H**) The knee extension rate of torque development is enhanced by GDF8 Ab treatment 2 weeks after ACLT injury. Dashed lines in (G) and (H) represent mean values from uninjured control mice from historical laboratory data. (**I**) Representative IHC images of quadriceps collagens 1 and 4 and collagen 1 (Col1)–GFP^+^ cells. scale bars, 100 μm. (**J** and **K**) GDF8 Ab treatment attenuates elevated abundance of Col1-GFP^+^ cells in quadriceps (J) 1 and (K) 2 weeks after ACLT [drug × injury interaction, *P* < 0.001 (J) and *P* = 0.019 (K)]. (**L** and **M**) GDF8 Ab treatment blocks the increase in collagens 1 and 4 in quadriceps (L) 1 and (M) 2 weeks after ACLT [drug × injury interaction, *P* = 0.006 (L) and *P* = 0.036 (M)]. *N* = 7 to 8 mice per group. *P < 0.05, **P < 0.01, ***P < 0.005, and ****P < 0.001 represent Šidák’s multiple comparison post hoc tests performed when significant interactions were detected via mixed models (B, C, J, K, L, and M) or independent sample *t* test (E to H). PLA Ab, placebo antibody; GDF8 Ab, GDF8 antibody; GFP, green fluorescent protein.

We performed in vivo muscle strength testing, immunohistomorphometry, fluorescent activated cell sorting (FACS) and collagen assays on quadriceps muscle after ACL transection (ACLT) in a murine PTOA model (Fig. 2A). The ACLT model induces rapid elevation in quadriceps GDF8 abundance (fig. S5A) that mimics clinical GDF8 response to ACL tear (fig. S1C). To conceptualize the quadriceps GDF8 increase following ACLT, a small cohort of mice were subjected to 1-week unilateral limb immobilization (fig. S5B) (*31*). Unilateral limb immobilization resulted in a significant elevation in quadriceps GDF8 that was less than half of the GDF8 increase observed 1 week after ACLT (fig. S5, A and B). To further interrogate the contribution of disuse to ACL injury–induced GDF8, a cohort of mice underwent unilateral limb immobilization for 1 week after ACLT. The combination of forced disuse plus ACLT did not meaningfully enhance quadriceps GDF8 beyond ACLT injury alone (fig. S5C), suggesting that post-injury disuse is not the sole effector of GDF8 or quadriceps atrophy (fig. S5, D and E) after ACL injury.

Following ACLT, mice were randomized to receive twice weekly subcutaneous injections of either a human monoclonal antibody specific to GDF8/myostatin [GDF8 Ab; 10 mg/kg; REGN647 (*23*), Regeneron Pharmaceuticals] or a placebo antibody (PLA Ab; 10 mg/kg; REGN1945, Regeneron Pharmaceuticals). One and 2 weeks after ACLT, PLA Ab–treated mice showed quadriceps muscle fiber atrophy (Fig. 2, B and C); anti-GDF8 treatment protected against atrophy (Fig. 2C). Anti-GDF8 treatment resulted in higher bodyweight 1 and 2 weeks after ACLT (fig. S6, A and B). In vivo tetanic torque analysis of quadriceps muscles at 2 weeks after ACLT showed increased muscle strength with anti-GDF8 treatment (Fig. 2, D to F, and fig. S6, C and D). Bodyweight-normalized torque, but not specific (i.e., fiber CSA-normalized) torque, was higher with anti-GDF8 treatment compared to PLA (fig. S6, E to H). In addition, the rate of torque development was greater with anti-GDF8 treatment 2 weeks after ACLT (Fig. 2, G and H). When normalized to peak torque, the rate of torque development showed a numerical, but not statistical, improvement with anti-GDF8 treatment 2 weeks after ACLT (fig. S6, I to K). We then evaluated the effects of GDF8 administration on SMAD3 phosphorylation (pSMAD3^Ser423/425^), both on whole quadriceps homogenates and myonuclei that are known to be pSMAD3^+^ after a knee injury (*32*). Immunoblot analysis revealed a reduction in pSMAD3 1 week after ACLT in animals treated with anti-GDF8 Ab (fig. S7, A to C). Probing myonuclei after ACLT demonstrated a reduction in pSMAD3 fluorescence intensity in animals treated with anti-GDF8 Ab as compared to placebo controls (fig. S7, D to F). Thus, twice weekly, anti-GDF8 Ab dosing decreased pSMAD3 and preserved quadriceps fiber size, tetanic torque, and rate of torque development after ACLT, as compared to placebo treatment.

### Anti-GDF8 treatment reduces fibrogenic cell abundance and quadriceps fibrosis following ACL injury

**Fig. 3.**
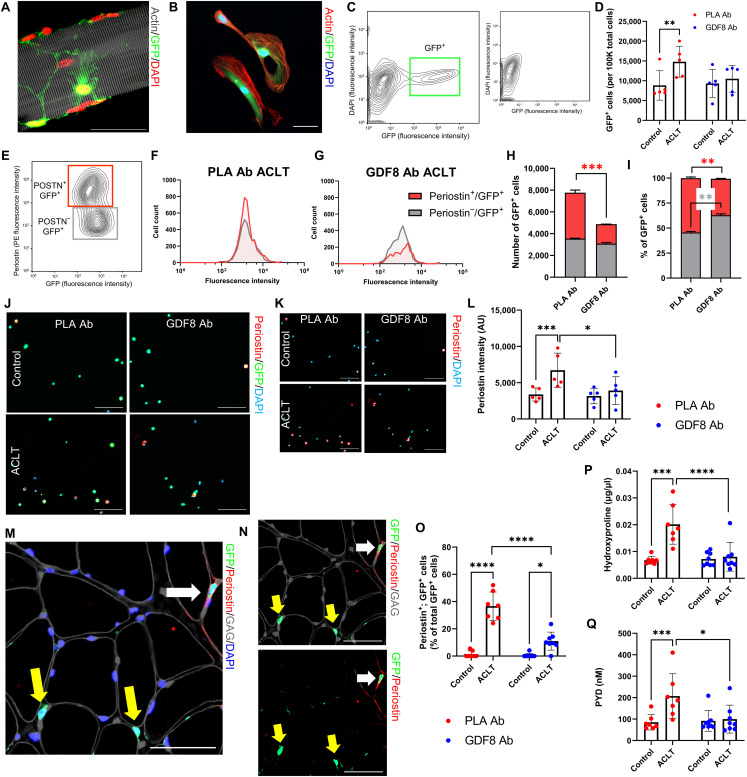
Treatment with anti-GDF8 antibody reduces muscle fibrogenic cell expansion and fibrosis following ACL injury. (**A** and **B**) Representative images demonstrating Col1-GFP^+^ cells on (A) a muscle fiber and (B) following FACS isolation. Scale bars, 50 μm. (**C**) Representative FACS plot indicating muscle Col1-GFP^+^ cells; small inset indicates GFP^+^ cell absence in wild-type mice. (**D**) ACLT increases quadriceps Col1-GFP^+^ cell abundance in PLA Ab–treated mice (drug × injury interaction, *P* = 0.029). (**E**) Col1-GFP^+^ cells were gated by periostin expression level. (**F** and **G**) Representative histograms demonstrating periostin cell counts and GFP fluorescence intensity in ACLT limbs of PLA Ab– and GDF8 Ab–treated mice. (**H** and **I**) GDF8 Ab treatment lowers (H) raw and (I) relative cell abundance of periostin^+^; Col1-GFP^+^ cells in the ACLT quadriceps [drug × cell type interaction, *P* = 0.012 for both (H) and (I)]. (**J** and **K**) Representative images of isolated Col1-GFP^+^ cells stained for periostin. Scale bars, 100 μm. (**L**) GDF8 Ab treatment attenuates elevated periostin intensity in Col1-GFP^+^ cells 1 week after ACLT (drug × injury interaction, *P* = 0.011). (**M** and **N**) Representative IHC images denoting periostin, Col1-GFP, glycosaminoglycans (GAG), and 4′,6-diamidino-2-phenylindole (DAPI) in ACLT-injured quadriceps. White arrow: periostin^+^; Col1-GFP^+^ cell; yellow arrows: periostin^−^; Col1-GFP^+^ cells. Scale bars, 50 μm. (**O**) GDF8 Ab treatment attenuates elevated frequency of quadriceps periostin^+^; Col1-GFP^+^ cells 1 week after ACLT (drug × injury interaction, *P* < 0.001). (**P** and **Q**) GDF8 Ab treatment rescues elevated (P) hydroxyproline abundance and (Q) pyridinoline (PYD) cross-linking in quadriceps muscle post-ACLT [drug × injury interaction, *P* = 0.002 for both (P) and (Q)]. *N* = 2 mice per group (E to I), 5 mice per group (C, D, and J to L), and 7 to 8 mice per group (M to Q). *P < 0.05, **P < 0.01, ***P < 0.005, and ****P < 0.001 represent Šidák’s multiple comparison post hoc tests performed when significant interactions were detected via mixed models. POSTN, periostin.

GDF8 signaling is a potent stimulator of fibrosis in skeletal muscle (*9*, *17*), with collagen 1 being the primary contributor to muscle fibrosis (*33*). Collagen 1–expressing cells were identified using a mouse line expressing enhanced green fluorescent protein (eGFP) under the control of the *collagen-*α*1(I)* promoter (mice denoted as Col1-GFP^+^) (*34*). To determine the effect of anti-GDF8 treatment on collagen 1–expressing cells, the quadriceps muscle was evaluated immunohistochemically for GFP^+^ cells after ACLT. Anti-GDF8 treatment reduced the abundance of GFP^+^ cells after ACLT as compared to placebo treatment (Fig. 2, I to K). This was associated with lower collagen 1 and 4 abundance in quadriceps muscle after ACLT (Fig. 2, L and M). GFP^+^ cells were primarily located outside of the basal lamina and exhibited adherent properties along the length of isolated single muscle fibers (Figs. 2I and 3A). To provide insight into the cellular identity of collagen 1–expressing cells, we performed FACS, capturing cells that were GFP^+^ (Fig. 3, B and C). GFP^+^ cells were isolated 1 week following ACLT, and placebo-treated animals had increased abundance of GFP^+^ cells isolated from the quadriceps after ACLT (Fig. 3D). Col1-GFP^+^ cells from the ACLT limb were further identified as fibro/adipogenic progenitor cells (FAPs) and satellite cells on the basis of labeling with Stem cell antigen 1 (Sca-1; GFP^+^, Sca-1^+^) and integrin α7 (α7; GFP^+^, α7^+^), respectively (fig. S8, A to D) (*33*). GDF8 Ab treatment did not induce substantive changes to the abundance of FAPs or satellite cells within the Col1-GFP^+^ cell fraction 1 week after ACLT injury (fig. S8, E to H). Unlabeled Col1-GFP^+^ cells (GFP^+^, Sca-1^−^, α7^−^) were further profiled for periostin (Fig. 3E), a marker that has been associated with myofibroblasts and other cell types that contribute to ECM remodeling post-injury (*35*). GDF8 Ab treatment mitigated the increase in periostin^+^; Col1-GFP^+^ cells in the quadriceps 1 week after ACLT (Fig. 3, F to I). Immunocytochemical analysis of Col1-GFP^+^ cells from the same mice showed an increase in periostin signal intensity 1 week after ACLT that was mitigated by GDF8 Ab treatment (Fig. 3, J to L). Further immunohistochemical analysis of quadriceps Col1-GFP^+^ cells demonstrated an elevated fraction that stained positive for periostin 1 week after ACLT that was mitigated by GDF8 Ab treatment (Fig. 3, M to O). To further explore the fraction of Col1-GFP^+^ cells that may be classified as myofibroblasts, immunohistochemical analysis of quadriceps demonstrated an elevated fraction that stained positive for myofibroblast marker alpha–smooth muscle actin (αSMA) (*33*) 1 week after ACLT that was mitigated by GDF8 Ab treatment (fig. S8, I to K). Gene expression analysis of *Acta2* revealed similar findings, with GDF8 Ab treatment attenuating the increase in *Acta2* 1 week following ACLT (fig. S8L). We observed frequent co-labeling of Col1-GFP^+^ cells with αSMA and periostin in the ACLT-injured quadriceps of PLA Ab–treated mice (fig. S8, M to O).

A majority of Col1-GFP^+^ cells were classified as FAPs (fig. S8, E to H), and additional immunocytochemical and immunohistochemical analysis of Col1-GFP^+^ cells showed a large proportion that stained positively for transcription factor 7 l2 (Tcf7l2) and platelet-derived growth factor receptor alpha (Pdgfrα), both canonical markers of fibroblasts and FAPs (*36*). The frequency of Tcf7l2^+^, Col1-GFP^+^ cells was not affected by ACLT or drug treatment nor was the fluorescent intensity of Tcf7l2 and Pdgfrα affected by ACLT or drug treatment (figs. S8, P to R, and S9, A to D).

To determine the effect of anti-GDF8 antibodies on muscle fibrosis, quadriceps muscles from 1-week treated mice were evaluated for hydroxyproline content, a measure of collagen deposition (*37*). Anti-GDF8 treatment reduced hydroxyproline content compared to placebo controls in quadriceps muscle after ACLT (Fig. 3P). Collagen fibrils undergo substantial posttranslational modification to alter the strength and stiffness of the extracellular matrix (ECM). Pyridinium cross-links are enzymatically added to collagen fibrils to increase passive muscle stiffness (*38*). Animals receiving anti-GDF8 treatment showed lower pyridinium cross-links in the quadriceps 1 week following ACLT (Fig. 3Q). To further quantify protection against fibrosis, quadriceps muscles were evaluated by Sirius Red staining. Anti-GDF8 treatment decreased fibrosis/collagen content in quadriceps muscles compared to placebo controls (fig. S10, A to C). Sirius Red–stained slides were also imaged under polarized light to quantify dense (red), intermediate (yellow), and loose (green) collagen (*39*, *40*). Quadriceps muscle in placebo-treated animals increased loose and intermediate collagen 1 week after ACLT with an increase in dense collagen 2 weeks after ACLT (fig. S10, D to H). Animals treated with anti-GDF8 antibodies showed a less robust alteration in collagen orientation in quadriceps muscle 1 and 2 weeks after ACLT (fig. S10, D to H). Together, these data indicate that treatment with anti-GDF8 Ab is effective in decreasing collagen 1–eGFP^+^ (Col1-eGFP^+^) cell abundance and quadriceps fibrosis following ACLT.

As a control for ACLT, mice underwent sham surgery, in which the joint capsule was opened but the ACL was not transected (fig. S11A). The sham-operated animals showed a significant, but subtle increase in quadriceps GDF8 (25% increase; fig. S11B), which was far less robust than the 280% elevation in quadriceps GDF8 noted with ACLT (fig. S5A). In addition, sham-operated mice did not show significant quadriceps fiber atrophy nor was there an increase in Col1-eGFP^+^ cell abundance (fig. S11, C to E). Sham surgery induced a slight increase in quadriceps collagen abundance (*P* = 0.06; fig. S11F).

### Distinct quadriceps transcriptomic signatures are associated with anti-GDF8 treatment and ACL injury

**Fig. 4.**
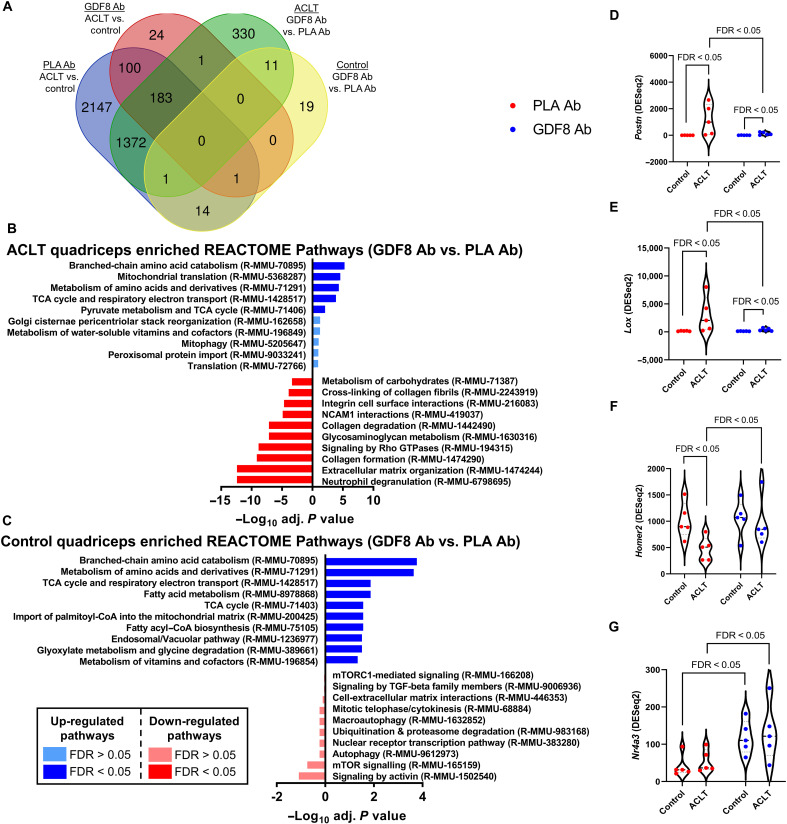
Transcriptomic analysis of most-changed genes by GDF8 inhibition in quadriceps muscle following ACL injury. RNA-seq analysis was performed on quadriceps muscles from the study depicted in Fig. 2A at 1 week after ACLT (*n* = 5 mice per group; samples were not pooled). Analysis was performed on PLA Ab– and anti-GDF8 Ab–treated control and ACLT quadriceps. (**A**) Venn diagram depicting a number of genes differentially expressed by limb and treatment group. (**B** and **C**) Most activated (blue) and inhibited (red) pathways associated with GDF8 inhibition in the (B) ACLT quadriceps and (C) control quadriceps using REACTOME Pathway Knowledgebase. Pathways having false discovery rate (FDR) *P* < 0.05 for either direction are bright colors; pathways having FDR *P* > 0.05 for either direction are muted colors. (**D**) Expression of *periostin* (*Postn*) is elevated following ACLT and is mitigated by treatment with GDF8 Ab. (**E**) Expression of *lysyl oxidase* (*Lox*) is elevated following ACLT and is mitigated by treatment with GDF8 Ab. (**F**) Expression of *Homer 2* (*Homer2*) is depressed following ACLT and is greater with anti-GDF8 Ab treatment. (**G**) Expression of *nuclear receptor 4A3* (*Nr4a3*) is elevated in control and ACLT quadriceps following anti-GDF8 Ab treatment. Gene expression levels were calculated using the DESeq2 method. FDR < 0.05 denote specific comparisons.

To identify the pathways that differ among placebo versus anti-GDF8–treated skeletal muscle in both control and ACLT limbs, RNA sequencing (RNA-seq) analysis was performed on quadriceps from these four groups. A large number of transcripts were substantially and distinctly changed by ACLT and anti-GDF8 treatment or placebo treatment (Fig. 4A). Unbiased investigation of the most affected cellular pathways associated with anti-GDF8 or placebo treatment after ACLT in quadriceps was performed using the REACTOME Pathways Knowledgebase (Fig. 4, B and C, and fig. S12) (*41*). In placebo-treated animals, ACLT significantly increased the abundance of genes associated with ECM remodeling in the quadriceps 1 week after injury (fig. S12). Notable pathways down-regulated by ACLT in placebo-treated animals included neddylation, ubiquitination and proteasome degradation, mTOR signaling, and translation (fig. S12).

Anti-GDF8 treatment significantly increased the abundance of genes associated with oxidative respiration and amino acid metabolism 1 week after ACLT as compared to placebo treatment (Fig. 4B). Another notable (although not significant) pathway up-regulated by anti-GDF8 treatment after ACLT includes gene changes associated with translation (fig. S13A), which is depressed following ACL injury (fig. S12A) (*42*). We did not observe changes in E3 ubiquitin ligase genes *Trim63* and *Fbxo32* (fig. S13, B and C). Noteworthy, pathways down-regulated by anti-GDF8 treatment after ACLT (compared to placebo) include genes associated with ECM remodeling, collagen biosynthesis, degradation, and cross-linking (fig. S14), including periostin (*Postn*) and lysyl oxidase (*Lox*) (Fig. 4, D and E), congruent with phenotypic fibrosis and cross-linked collagen data presented in Figs. 2 and 3. In addition, anti-GDF8 treatment protected against injury-induced decreases in *Homer2* abundance (Fig. 4F). Homer 2 antagonizes protein degradation (*43*), and anti-GDF8 treatment may act to preserve muscle fiber size through Homer 2 after ACLT.

There were substantially fewer genes affected by anti-GDF8 treatment in the control limb as compared to placebo; however, notable pathways include gene changes associated with activation of the tricarboxylic acid cycle (TCA) and down-regulation of SMAD2/3 transcriptional activity (Fig. 4C). Anti-GDF8 treatment increased the abundance of *Nr4a3* in both the control and ACLT limb (Fig. 4G). The nuclear receptor Nor-1/Nr4a3 is one of the most exercise- and inactivity-responsive genes for its role in mediating the metabolic response to exercise (*44*).

### Longer-term anti-GDF8 dosing increases body mass and muscle fiber size and reduces muscle fibrosis

We performed a longer-term study evaluating animals undergoing treatment for 4 weeks after ACLT to assess muscle pathological features, such as fibrosis and atrophy, associated with knee OA (*26*, *27*). C57BL/6J mice were injected subcutaneously with anti-GDF8 or PLA Abs, at a dosage of 10 mg/kg, twice weekly, for a total of four consecutive weeks after ACLT (fig. S15A). Mice receiving anti-GDF8 Ab had increased body mass (fig. S15B) and quadriceps fiber CSA (both control and ACLT limbs; fig. S15C). Anti-GDF8–treated animals trended toward greater in vivo quadriceps tetanic torque (*P* = 0.09; fig. S15, D and E), but there was no difference in tetanic torque when normalized to bodyweight or fiber size (specific torque; fig. S15, F and G). Quadriceps fibrosis was reduced with anti-GDF8 treatment 4 weeks after ACLT (fig. S16, A to G). Together, these data indicate that longer-term treatment with anti-GDF8 Ab after ACLT increases body mass and muscle mass and reduces muscle fibrosis.

### GDF8 knockout reduces phospho-SMAD3 and prevents quadriceps atrophy, weakness, and fibrosis following ACL injury

**Fig. 5.**
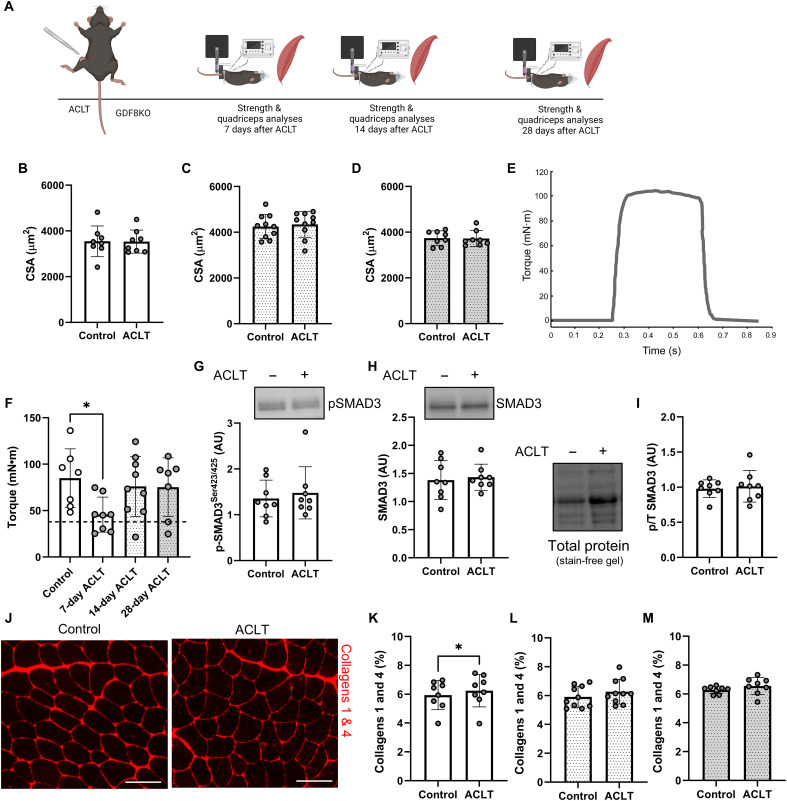
GDF8 knockout preserves quadriceps size, strength, and quality following ACL injury. (**A**) Study diagram; mice were studied at 7, 14, and 28 days after ACLT. (**B** to **D**) GDF8KO preserves quadriceps muscle fiber size (B) 7, (C) 14, and (D) 28 days after ACLT. (**E**) Representative tetanic graph from GDF8KO mouse knee extension peak isometric torque 14 days after ACLT. (**F**) Knee extension peak isometric torque is impaired 7 days after ACLT in GDF8KO mice (the dashed line represents the mean value from wild-type mice), but the strength impairment is rescued in GDF8KO mice 14 and 28 days after injury. (**G**) GDF8KO mitigates induction of pSMAD3 in the quadriceps 7 days following ACLT. (**H**) SMAD3 protein expression in GDF8KO mouse quadriceps muscle 7 days following ACLT. (**I**) pSMAD3 normalized to total SMAD3 in GDF8KO mouse quadriceps muscle 7 days following ACLT. (**J**) Representative IHC images of collagens 1 and 4 in control and ACLT quadriceps muscle. Scale bars, 100 μm. (**K** to **M**) GDF8KO protects against elevated collagen content in quadriceps (K) 7, (L) 14, and (M) 28 days after ACLT. *N* = 8 to 10 mice per group. *P < 0.05 via one-factor analysis of variance (ANOVA).

We performed in vivo muscle performance testing, immunohistomorphometry, immunoblotting, and collagen assays on quadriceps muscle after ACLT in GDF8 knockout (GDF8KO) mice (Fig. 5A). GDF8KO mice had an approximate doubling of muscle mass compared to Col1-eGFP mice (fig. S17, A to C), and genetic loss of GDF8 protected against quadriceps atrophy following ACLT (Fig. 5, B to D). In vivo tetanic torque analysis of quadriceps muscles at 1 week after ACLT point showed a deficit in muscle strength in GDF8KO mice, but quadriceps torque was restored to control limb values at 2 and 4 weeks after ACLT (Fig. 5, E and F, and fig. S17, D and E). Notably, GDF8KO mouse torque values normalized to fiber CSA (“specific torque”) are similar to Col1-eGFP mice (fig. S6, G and H). GDF8KO mice exhibit markedly increased muscle mass from an increased number of muscle fibers (hyperplasia) formed during development (*12*) and increased fiber size (hypertrophy) in adulthood (Figs. 2, B and C, and 5, B to D). Immunoblot analysis revealed no effect of ACLT on pSMAD3 1 week after ACLT in GDF8KO animals (Fig. 5, G to I).

To determine the effect of GDF8KO on muscle fibrosis, quadriceps muscles were evaluated histochemically and immunohistochemically for collagen content after ACLT. Collagen abundance exhibited minimal differences in GDF8KO animals between ACLT and control limbs evaluated at 1, 2, and 4 weeks after ACLT (Fig. 5, J to M, and fig. S17, F to I). The assessment of collagen organization via polarized light showed alterations in the ACLT-affected quadriceps in GDF8KO mice (fig. S17, J to P), but this was not associated with differences in total collagen content. Hydroxyproline and pyridinium cross-link assessment at 1 week after ACLT showed no difference between limbs in GDF8KO mice (fig. S17, Q and R). Together, these data indicate that GDF8KO mitigates SMAD3 signaling and is effective in preventing quadriceps atrophy, weakness, and fibrosis following ACLT.

### Anti-GDF8 dosing and GDF8KO suppress injury-induced molecular, cellular, and joint structure change in PTOA

**Fig. 6.**
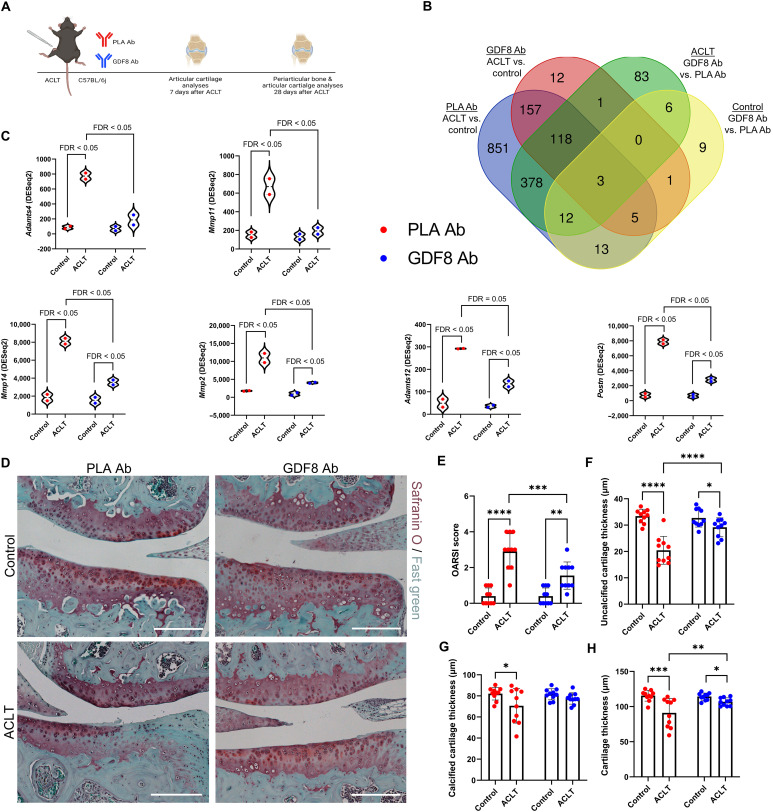
Treatment with anti-GDF8 antibody mitigates the severity of PTOA. (**A**) Mice were treated biweekly; articular cartilage was collected from control and ACL transected knees at 1 week after ACLT for transcriptomic analysis and 4 weeks after ACLT for histopathology. (**B**) Eight mice were studied per group, and RNA was pooled from four mice to yield two biological replicates per group. A number of genes differentially expressed by limb (control and ACLT) and treatment (placebo- and GDF8-treated) groups were shown. (**C**) Expression of matrix metalloproteinases and factors affecting cartilage remodeling were elevated in articular cartilage 7 days after ACLT that were mitigated by GDF8 Ab treatment. Gene expression levels were calculated using DESeq2. (**D**) Representative images of safranin O and fast green from knee joints 4 weeks after ACLT. Scale bars, 200 μm. (**E**) Medial tibial plateau joint score (OARSI scoring system) shows the attenuation of PTOA severity in animals treated with anti-GDF8 Ab (drug × injury interaction, *P* = 0.005). (**F**) Uncalcified cartilage zones show thinning 28 days after ACLT that is protected by treatment with anti-GDF8 Ab (drug × injury interaction, *P* < 0.001). (**G**) Calcified cartilage zones show thinning 28 days after ACLT in placebo-treated animals (drug × injury interaction, *P* = 0.048). (**H**) Anti-GDF8 Ab treatment partially protects against total articular cartilage thinning 28 days after ACLT (drug × injury interaction, *P* = 0.019). *N* = 8 to 10 mice per group. *P < 0.05, **P < 0.01, ***P < 0.005, and ****P < 0.001 represent Šidák’s multiple comparison post hoc tests performed when significant interactions were detected via mixed models.

ACLT induces moderate to severe PTOA in mice (*45*), and we evaluated animals undergoing anti-GDF8 treatment for 1 or 4 weeks after ACLT to assess PTOA pathogenesis (Fig. 6A). To identify the pathways that differ among placebo versus anti-GDF8–treated animals in both control and ACLT limbs, RNA-seq analysis was performed on articular cartilage from these four groups 1 week after ACLT. Because of low RNA yields from cartilage, tissue was pooled from four mouse limbs (four pooled control and four pooled ACLT limbs) with eight mice per group studied in total (*n* = 2 pooled samples per group). A large number of transcripts were substantially and distinctly changed by ACLT + placebo treatment compared to ACLT + anti-GDF8 treatment (Fig. 6B). Unbiased investigation of the most affected cellular pathways associated with anti-GDF8 treatment after ACLT in articular cartilage was performed using the REACTOME Pathways Knowledgebase (fig. S18) (*41*). Most notably, anti-GDF8 treatment significantly decreased the abundance of genes associated with ECM organization and degradation, as well as matrix metalloproteinase (MMP) activation (figs. S18A and S19). Anti-GDF8 treatment provided partial protection against increased abundance of several metalloproteinases associated with cartilage erosion in PTOA, including *Adamts4*, *Mmp11*, *Mmp14*, *Mmp2*, and *Adamts12* (Fig. 6C) (*46*). Animals treated with anti-GDF8 were also partially protected against an increase in *Postn* abundance (Fig. 6C). Elevated levels of periostin (*Postn*) in cartilage are associated with both age-related OA and PTOA (*47*), and periostin loss of function protects mice against PTOA (*48*). Anti-GDF8 treatment significantly increased the abundance of genes associated with the cell cycle/mitosis and TCA cycle 1 week after ACLT (fig. S18A).

There were substantially fewer genes affected by anti-GDF8 treatment in the control limb with no statistically significant enrichment of up-regulated pathways. However, notable down-regulated pathways include gene changes associated with collagen degradation and respiratory electron transport (fig. S18B). In both placebo- and anti-GDF8–treated animals, ACLT significantly increased the abundance of genes associated with ECM remodeling, glycosaminoglycan remodeling, ECM proteoglycans, and activation of MMPs in articular cartilage 1 week after injury (fig. S20, A and B). Anti-GDF8–treated animals had relatively few pathways statistically decreased in the articular cartilage 1 week after ACLT (fig. S20A). In placebo-treated animals, ACLT significantly decreased the abundance of genes associated with the TCA cycle, respiratory electron transport, and pyruvate metabolism 1 week after injury (fig. S20B).

ACLT reduced proteoglycan staining and resulted in cartilage thinning and surface irregularities 4 weeks after ACLT (Fig. 6D) (*49*). Treatment with anti-GDF8 partially protected articular cartilage erosion such that proteoglycan staining was more robust and integrity was better preserved 4 weeks after ACLT (Fig. 6, E to H).

As a control for ACLT, a cohort of mice underwent sham surgery, in which the joint capsule was opened but the ACL was not transected. In sham-injured animals, the articular cartilage was relatively intact with a smooth surface and uniform organization 4 weeks after ACLT (fig. S21). Joints from GDF8KO animals were assessed as well for articular cartilage integrity 4 weeks after ACLT. Lesions in the proteoglycan staining were apparent 4 weeks after ACLT, but the severity of PTOA in GDF8KO knee joints was less robust than in placebo-treated mice (Fig. 6E and fig. S22). Together, these data indicate that targeting GDF8 is effective in attenuating PTOA severity following ACLT.

### Anti-GDF8 dosing and GDF8KO preserve subchondral and trabecular bone microarchitecture following ACLT injury

**Fig. 7.**
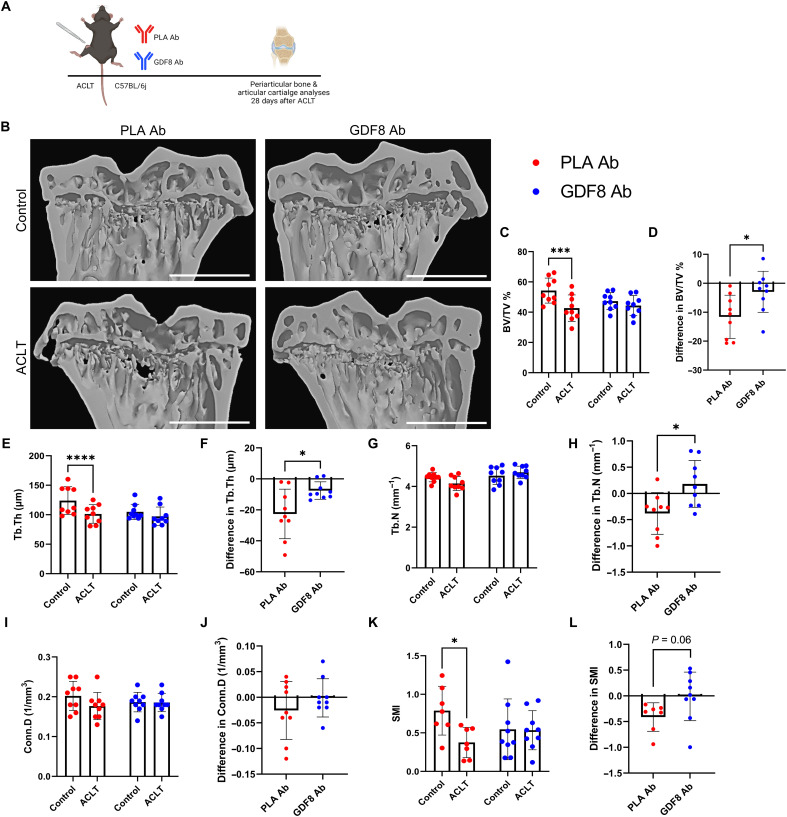
GDF8 antibody preserves periarticular bone microarchitecture following ACL injury. (**A**) Study diagram; mice were treated biweekly with subcutaneous injections; whole knees were fixed for micro–computed tomography (micro-CT) analysis 4 weeks after ACLT. (**B**) Representative micro-CT images showing three-dimensional reconstruction of coronal sections. Scale bars, 1 mm. (**C** to **L**) Morphometric parameters of tibia subchondral trabecular bone. Anti-GDF8 treatment preserved bone volume fraction [BV/TV % (C and D), drug × injury interaction, *P* = 0.023], trabecular thickness [Tb.Th (E and F), drug × injury interaction, *P* = 0.017], and partially preserved trabecular number [Tb.N (G and H), drug × injury interaction, *P* = 0.020] 28 days after ACLT. There were no significant effects of ACLT or anti-GDF8 treatment on connectivity density [Conn.D (I and J)], but anti-GDF8 treatment protected against a loss of structure model index [SMI (K and L), drug × injury interaction, *P* = 0.047]. *N* = 9 mice per group. *P < 0.05, ***P < 0.005, and ****P < 0.001 represent Šidák’s multiple comparison post hoc tests performed when significant interactions were detected via mixed models (C, E, G, I, and K); *P < 0.05 via independent sample *t* test (D, F, H, J, and L).

PTOA is clinically characterized by a loss of cartilage; however, subchondral bone remodeling plays a significant role in the initiation and progression of the disease (*28*). We evaluated the proximal tibial metaphysis in animals undergoing anti-GDF8 treatment for 4 weeks after ACLT to assess periarticular bone remodeling during PTOA (Fig. 7, A and B). The proximal tibial metaphysis was chosen for analysis as BMD loss in the proximal tibial metaphysis in humans was most significantly correlated with acute GDF8 induction following ACL injury (Fig. 1J and fig. S3E). Bone volume fraction (BV/TV) was decreased in placebo-treated animals, but treatment with anti-GDF8 rescued deficits in bone volume fraction (Fig. 7, C and D). BMD was not different between groups (fig. S23, A and B). Micro–computed tomography (Micro-CT) images in the coronal plane revealed that anti-GDF8 treatment significantly protected against loss of trabecular thickness (Tb.Th) and increased trabecular porosity (Tb.Po) compared to placebo-treated animals (Fig. 7, E and F, and fig. S23, C and D). The relative loss of trabecular number (Tb.N) was also protected by anti-GDF8 treatment (Fig. 7, G and H). Trabecular separation (Tb.Sp) and connectivity density (Conn.D) were not different between groups (Fig. 7, I and J, and fig. S23, E and F); however, structure model index scores were protected with anti-GDF8 treatment (Fig. 7, K and L). Medial subchondral bone plate thickness (Sb.Pl.Th) and porosity (Sb.Po) were not different in either group (fig. S23, G to J). Coronal plane micro-CT analysis was also performed on the proximal tibial metaphysis in GDF8KO animals 4 weeks following ACLT (fig. S24, A and B). GDF8KO protected the ACLT-injured limb from loss of trabecular and subchondral bone (fig. S24, C to L). Together, these data indicate that genetic and antibody-targeting of GDF8 is effective in preserving tibial metaphysis trabecular bone volume and structure following ACLT-initiated PTOA.

## DISCUSSION

We evaluated the in vivo efficacy of a humanized antibody against GDF8 aimed at promoting muscle strength and size, reducing muscle fibrosis, and mitigating PTOA. This preclinical testing was carried out in Col1-eGFP mice and GDF8KO mice with concordant results across quadriceps muscle, articular cartilage, and subchondral and trabecular bone in short- and long-term dosing schemes. We report GDF8 abundance and concentration in human quadriceps and serum that varied over time after ACL injury and reconstruction. Acute GDF8 induction in the quadriceps after ACL injury explained significant variance in measures of serum GDF8 concentration, muscle weakness, and atrophy, as well as periarticular bone loss obtained several months following ACL injury and reconstruction. Both temporal-specific variability and patient-specific variability in quadriceps GDF8, as well as efficacy results in a preclinical PTOA model, reveal an opportunity for therapeutic targeting in an acute human intervention.

A majority of individuals who sustain an ACL tear go on to develop PTOA (*5*, *6*), and mouse ACLT models develop moderate-to-severe PTOA within a month of injury (*45*). Our findings that anti-GDF8 therapy attenuated cartilage MMP expression and the severity of PTOA is the first demonstration of our knowledge of a critical causative role for GDF8 in the etiology of knee PTOA. Aberrant transforming growth factor–β signaling is causative in subchondral bone sclerosis (*50*) and the development of joint OA (*51*), and the protection against cartilage destruction after ACLT reflects a pivotal role for GDF8 specifically in the etiology of PTOA. In healthy articular cartilage, matrix turnover and MMP activity remain relatively low, and treatment with anti-GDF8 Ab markedly dampened measures of cartilage matrix remodeling and MMP abundance acutely after ACLT. Anti-GDF8 effects were further realized with the preservation of trabecular bone and cartilage integrity 1 month after a knee injury. Our findings strongly suggest that targeting GDF8 rapidly after traumatic joint injury may diminish cartilage matrix destruction and protect against PTOA development.

Injuries to the ACL result in persistent quadriceps muscle atrophy and weakness despite surgical reconstruction and intense postoperative rehabilitation programs (*11*, *52*, *53*). We therefore assessed the protective effect of a humanized monoclonal GDF8 Ab administered at the time of ACLT on muscle size and function. Anti-GDF8 treatment after ACLT improved quadriceps strength by 58% 2 weeks following injury so as to be not different from normative healthy values. After treatment with anti-GDF8 Ab, mice were protected against quadriceps atrophy as quickly as 1 week, and sustained through 4 weeks, after injury. GDF8KO restored peak isometric quadriceps tetanic torque at 2 and 4 weeks after ACLT, in addition to preserving quadriceps muscle fiber size. Both anti-GDF8–treated and GDF8KO mice displayed deficits in torque 1 week following injury despite an absence of measurable atrophy. Early strength deficits following ACL injury and reconstruction may be attributable to a reduced neural drive to knee extensor muscles (*54*) that may exist irrespective of muscle fiber size maintenance via anti-GDF8 therapies (*55*, *56*). Speed, perhaps more so than magnitude, of quadriceps torque production predicts knee function after ACL injury (*57*), and anti-GDF8 Ab treatment improved the rate of torque development of the ACLT-injured quadriceps. Incomplete recovery of muscle strength following ACL injury can alter knee biomechanics and may predispose patients to develop OA. Our findings support anti-GDF8 therapy to prevent weakness and atrophy after injury.

Our previous work indicates sustained quadriceps fibrosis after ACL injury (*25*, *32*, *40*) that persists in patients with knee OA (*26*, *27*). We also report that GDF8 induces human muscle fibroblast proliferation and predicts FAP abundance following ACL injury (*9*). Anti-GDF8 treatment reduced quadriceps muscle fibrosis and expansion of collagen 1–expressing cells. Aberrant GDF8 signaling is a driver of muscle fibrosis (*17*), and induction of GDF8 within the quadriceps after ACL injury appears unique among transforming growth factor–β superfamily members (*9*, *11*). Our data suggest anti-GDF8 strategies mitigate periostin and αSMA activation, which can occur in myofibroblasts involved in muscle remodeling in response to injury (*35*). This, in turn, improved quadriceps collagen organization and cross-linking, which may restore passive mechanical properties within the muscle after joint injury (*38*). Muscle fibrosis is considered irreversible across many acute and chronic pathologies (*58*), underscoring the need for rapid delivery of therapies (*59*). Two doses of anti-GDF8 Ab reduced the muscle fibrotic burden, restoring values to those of the uninjured limb. We observed a significant down-regulation of pathways concerning ECM organization, collagen biosynthesis, and cross-linking with anti-GDF8 treatment in the ACLT limb. Dosing acutely after injury may reflect the intervention window in the ACLT injury model, given the rapid onset of collagen remodeling after the initial ACL tear (*9*, *40*).

We recently described similarities in the quadriceps transcriptome signature of mice after ACLT as compared to human participants following ACL injury (*42*). We sought to assess the effect of a humanized monoclonal GDF8 Ab on quadriceps transcriptome alterations after ACLT. The mechanism of action for anti-GDF8–based therapies is widely regarded as the repression of muscle protein degradation (*56*) through activation of SMAD signaling and ubiquitin-proteasome activation. Genetic and antibody targeting of GDF8 reduced SMAD3 phosphorylation after ACL injury, but we did not observe gene changes in canonical E3 ubiquitin ligases *Trim63* and *Fbxo32* after ACLT. Our prior work suggests diminished protein anabolism potentiates quadriceps atrophy after ACL injury (*42*), and anti-GDF8 Ab treatment may act to preserve quadriceps size through enhanced translation. Our findings support intriguing evidence that specific targeting of GDF8 may support improvements in quadriceps proteostasis through improved protein translation.

Much has been written regarding the challenges faced by the clinical translation of anti-GDF8 therapies (*60*, *61*). Clinical trials using GDF8 inhibitors have covered patients with various muscular dystrophies, idiopathic inflammatory myopathies, cancer cachexia, chronic obstructive pulmonary disease (COPD), and aging/sarcopenia (*61*). Many of these trials have been met with limited/modest success (e.g., increases in lean mass but poorer improvements in functional outcomes), and this can be attributed to several potential explanations. In many of these genetic and chronic conditions, patients are concurrently treated with corticosteroids, which have been shown to completely counteract the hypertrophic effects of GDF8 inhibition in skeletal muscle (*62*). In addition, decreased GDF8 levels (both within muscle and systemically) have been reported in patients with muscular dystrophy and older adults who show greater disease progression/muscle wasting (*63*, *64*), which would limit the effectiveness of anti-GDF8 strategies. Last, some anti-GDF8 therapies also bind GDF11, which leads to a cap on GDF8 inhibition. Together, these hurdles have likely hampered the true potential of anti-GDF8 therapies. ACL rupture and subsequent PTOA as clinical indications for anti-GDF8 therapy offer a number of key differences that address the complications that have limited the success of anti-GDF8 treatment in genetic and chronic conditions. (i) Corticosteroid prescription is not standard of care following ACL injury, which will allow GDF8 inhibition to better rescue muscle atrophy and weakness. (ii) We (*9*) and others (*11*) have shown that GDF8 levels peak in the muscle and serum shortly following initial ACL injury, indicating an optimal time to begin anti-GDF8 treatment. With genetic conditions such as muscular dystrophy or chronic conditions such as cancer cachexia/COPD, there is often not a precipitating event to benchmark the initiation of pharmacotherapy. With a majority of ACL injuries leading to PTOA within 10 to 15 years, irrespective of reconstruction/treatment of the injured ligament, there is a clear catalyst to guide treatment strategies. Supportive of this point are our data on human participants connecting GDF8 signaling to musculoskeletal deficits. Our findings underscore a remaining need to establish the duration of elevated muscle GDF8 signaling to guide treatment windows. (iii) The GDF8 neutralizing monoclonal antibody that we used shows no detectable binding affinity for GDF11 (*23*), alleviating a potential cap on GDF8 inhibition. Together, these concepts support GDF8 as a treatable and modifiable molecular effector of poor outcomes (muscle, bone, and cartilage) following traumatic joint injury.

Our study is not without limitations; our mouse injury involves surgical transection of the ACL, differing from clinical ACL cases that are overwhelmingly closed-compartment ruptures. While invasive, the ACLT induced comparable deficits in muscle size, strength, and periarticular bone as observed in our clinical participants. In addition, experiments in sham-operated mice showed a very modest increase in quadriceps GDF8 and no robust phenotypic alterations in either the quadriceps or articular cartilage. Compression of PTOA development in mice makes the establishment of treatment guidelines for patients challenging, where PTOA develops over 10 to 15 years after injury. A recent review article sought to define a relationship between mouse and human age, establishing that during adulthood, 2.6 mouse days is equivalent to 1 human year (*65*). The onset of moderate PTOA 28 days after injury in mice would then equate to ~11 years following a knee injury in a human, a time when the incidence of PTOA in humans after ACL reconstruction is ~40% (*66*). While supportive of our premise, guidelines for treatment duration in patients following a knee injury need to be established with consideration given to potential off-target effects of GDF8 inhibition (*67*).

In conclusion, muscle GDF8 induction acutely following a knee injury in humans predicts prolonged deficits in muscle size, strength, and periarticular bone loss. We have demonstrated that inhibition of GDF8 through humanized monoclonal antibody or genetic knockout protects against muscle weakness and fibrosis while preserving trabecular bone and mitigating PTOA. Our results provide reinvigorated support for anti-GDF8 therapies in the context of knee OA and recovery following joint injury.

## MATERIALS AND METHODS

Please see the Supplemental Materials for complete methods.

### Human study methods: Study group and biospecimen collection

This study was approved by the Institutional Review Board (protocol #43046) at University of Kentucky (UK) and performed in accordance with the ethical standards of the 1964 Declaration of Helsinki. All participants provided informed consent and were confirmed to have a complete tear of the ACL; subject demographic information can be found in table S1. Bone-patellar tendon-bone graft ACL surgeries were completed by one of two physicians at the UK Orthopaedic Surgery and Sports Medicine. Muscle biopsy specimens from the ACL-injured (ACL-inj) and contralateral healthy vastus lateralis were collected immediately before ACL surgical reconstruction (ACLR) with follow-up biopsies of the ACL-injured limb obtained 1 week after ACLR and 4 months after ACLR.

Periarticular BMD was assessed with x-ray absorptiometry (DXA) scans (Lunar iDXA, GE Healthcare) and was completed pre-ACLR and 6 months after ACLR in both the ACL-injured and healthy limbs (*68*).

Participants’ peak isometric torque and the mean slope of the torque-time curve between 20 and 80% of the first 200 ms from muscle contraction onset (rate of torque development) were evaluated in the healthy limb and ACL-injured limbs before ACLR and at 4 and 6 months after ACLR as previously described (*69*).

### Mouse study methods and drug treatment

This study was approved by the Institutional Animal Care and Use Committee (protocol #2019-3241) at UK in accordance with the National Institutes of Health (NIH) Guide for the Care and Use of Laboratory Animals. C57BL6/J mice were obtained from the Jackson Laboratory (Bar Harbor, ME). GDF8KO mice were obtained from Regeneron Pharmaceuticals (Tarrytown, NY) (*23*). Col1-eGFP mice were a gift from D. Brenner (*34*). Mice were studied between 4 and 5 months of age and both males and females were used in all experiments. For drug treatments, C57BL/6J and Col1-eGFP mice were injected subcutaneously twice weekly with a human monoclonal antibody specific to GDF8/myostatin [10 mg/kg; GDF8 Ab; REGN647 (*23*), Regeneron Pharmaceuticals] or a PLA Ab (10 mg/kg; REGN1945, Regeneron Pharmaceuticals). The GDF8 Ab is a fully humanized monoclonal anti-myostatin antibody with an IgG4 Fc domain which selectively inhibits pro-, latent, and mature myostatin without binding GDF11 or activin A (*23*).

### Surgically induced PTOA mouse model

Surgical ACLT was performed on a single limb for all mice, with the contralateral hind limb serving as an internal healthy control, similar to our prior work (*42*).

### In vivo tetanic torque and RTD

Muscle isometric mechanics were assessed as described previously (*70*) on the quadriceps muscle using a Whole Animal System for Mice (catalog no. 1300A; Aurora Scientific, ON, Canada).

### Micro–computed tomography analysis

Before decalcification, whole fixed knees from control and ACLT limbs were scanned using a Skyscan 1276 micro-CT (Bruker, Billerica, MA). Knees were scanned 28 days after ACLT in both GDF8 Ab– and PLA Ab–treated mice as well as GDF8KO mice. Whole knees were scanned with a voxel size of 9 μm for analysis of three-dimensional bone structure with the following scanning parameters used for both limbs: energy = 50 kV, intensity = 200 μA, integration time = 300 ms, and 0.25-mm Al beam hardening filter similar to our prior work (*71*).

### Immunohistochemistry

Frozen human and mouse quadriceps tissue was sectioned (7 μm thickness) on a cryostat and staining was performed as previously published (*72*–*76*).

### RNA sequencing

RNA was isolated and sequenced from quadriceps and articular cartilage from both control and ACLT limbs in both GDF8 Ab– and PLA Ab–treated mice 7 days after ACLT (*42*, *77*). Quadriceps RNA-seq data are deposited in Gene Expression Omnibus: GSE230708. Cartilage RNA-seq data are deposited in Gene Expression Omnibus: GSE230736.

### FACS of Col1-eGFP^+^ cells

Col1-eGFP^+^ cells were isolated from the quadriceps muscles of mice 7 days after ACLT by enzymatic digestion and FACS via a modified protocol (*78*). Entire quadriceps muscles were dissected from the hind limb and mechanically minced under sterile conditions.

### Hydroxyproline and pyridinium cross-link assays

To determine total collagen content, hydroxyproline was measured with a modified protocol using a commercially available Hydroxyproline Assay Kit (MAK008, Millipore Sigma, Darmstadt, Germany) (*37*). Pyridinium (PYD) cross-link (pyridinoline and deoxypyridinoline) concentration was determined by enzyme-linked immunoassay Metra PYD EIA kit (Quidel Corporation, San Diego, CA), as previously described using skeletal muscle hydrolysate (*79*).

### Statistical analysis

For human studies, statistical analysis was performed on continuous data using mixed models with no sphericity assumption to account for repeated observations within the same subject. Dunnett’s multiple comparison tests were performed with individual variances computed for each comparison to specifically compare post-injury data with the healthy limb value. For mouse studies, we fit a series of mixed models with drug as a fixed effect (GDF8 Ab versus PLA Ab) and a random effect modeling correlation between limbs within each mouse (comparing ACLT and control). Separate comparisons were performed within each distinct time point (we did not compare across time points; e.g., 7, 14, or 28 days after ACLT was each analyzed separately). If significant interactions were identified (drug × injury, *P* < 0.05), then Šidák’s multiple comparison post hoc testing was performed. Pairwise comparisons of interest via Šidák’s multiple comparison post hoc tests are denoted in figures with asterisks defined for given *P* value references. Drug × injury interaction *P* values are provided for all comparisons in the respective figure legends. For instances where two independent samples were compared, we performed two-sample *t* tests. Statistical testing is denoted in all figure legends. Data are presented as means ± SD with individual data points overlaid on bar charts. No assumption violations were observed for parametric statistical testing for human and mouse data; these assumptions include distributional and equal variance. *P* values < 0.05 were considered statistically significant. All analyses were performed with GraphPad Prism 9.0.2.
