# Supplementary material for: GDF8 inhibition enhances musculoskeletal recovery and mitigates posttraumatic osteoarthritis following joint injury
Source: Sci Adv. 2023 Nov 29;9(48):eadi9134. doi: 10.1126/sciadv.adi9134 (PMC10686569; doi:10.1126/sciadv.adi9134)
Supplement: Supplementary file 1 — Supplementary Materials and Methods Figs. S1 to S24 Tables S1 and S2 [file sciadv.adi9134_sm.pdf]

Science Advances
AAAS

Supplementary Materials for

GDF8 inhibition enhances musculoskeletal recovery and mitigates posttraumatic osteoarthritis following joint injury

Camille R. Brightwell *et al*.

Corresponding author: Christopher S. Fry, cfr223@uky.edu

Sci. Adv. **9**, eadi9134 (2023)
DOI: 10.1126/sciadv.adi9134

This PDF file includes:

Supplementary Materials and Methods
Figs. S1 to S24
Tables S1 and S2

SUPPLEMENTAL MATERIALS

Supplemental Materials and Methods

Human study methods: study group and biospecimen collection

This study was approved by the Institutional Review Board (protocol #43046) at the University of Kentucky (UK) and performed in accordance with the ethical standards of the 1964 Declaration of Helsinki. Informed written consent was obtained from each patient before participation in this study. Additionally, all minors in this study assented to participation. Participants had to be between 15-40 years of age, with their ACL tear occurring no more than 6 months prior to enrollment and register at least a 5/10 on the Tegner Activity Scale, or have torn their ACL during an athletic activity. Exclusion criteria included complete prior ACL injury/reconstruction, total knee dislocation, spinal fusion, taking anti-coagulant medication, recent inflammation or infection of the lower limb(s), diabetes, pregnancy, diminished capacity to provide informed consent, or inability to attend physical therapy or study visits. The diagnosis of the ACL injury was made by an orthopedic surgeon via magnetic resonance imaging (MRI) and physical examination. All subjects were confirmed to have a complete tear of the ACL; 18 of 23 subjects also had a meniscus tear. Subject demographic information can be found in Supplemental Table 1. Seventeen subjects completed all study visits; due to human subjects' research restrictions in place during the peak of the COVID19 pandemic, a subset of subjects did not undergo certain follow-up visits. Sample number is denoted in all figure legends. Bone-patellar-bone graft ACL surgeries were completed by one of two physicians at the University of Kentucky Orthopedic Surgery & Sports Medicine practice.

Muscle biopsy specimens from the ACL-injured (ACL-inj) and contralateral Healthy vastus lateralis were collected immediately prior to ACL surgical reconstruction (ACLR). Percutaneous muscle biopsy specimens (~100 mg) from the vastus lateralis were obtained after application of local anesthesia (lidocaine). Follow-up biopsies of the ACL-injured limb were obtained one-week post-ACLR (1wk post-ACLR) and four months post-ACLR (4mo post-ACLR) at the UK Center for Clinical and Translational Science. Samples were dissected free of fat and connective tissue and were quickly blotted to remove excess blood; approximately 50 mg each

were 1) immediately flash frozen in liquid nitrogen for protein analyses or 2) mounted in OCT on cork and flash frozen in liquid nitrogen–cooled 2-methylbutane. Samples were stored at –80°C until processing. Blood samples were collected and processed for serum at intervals during recovery from ACL-injury: pre-ACLR, 1wk, 2wk, 1mo, 4mo, 6mo post-ACLR.

Periarticular bone mineral density (BMD) was assessed with DXA scans (Lunar iDXA, GE Healthcare) and was completed pre-ACLR and 6mo post-ACLR in both the ACL-injured and Healthy limbs. We utilized a validated protocol for determining BMD in the femur and tibia (*68*). Participants completed the DXA scan lying on their backs while fully extending the scanned leg with the foot supported and rotated toward midline. Images were obtained frontally so that the patella and fibular head were both completely visible in the scan and that the joint space was horizontal. The DXA laser light was positioned at 3.5 cm above the proximal border of the patella. Two regions were measured: the proximal tibia metaphysis was measured by the distal horizontal edge of the region of interest placed at the most distal point of contact between the fibular head and the tibia while the proximal edge was placed at the upper edge of the fibular head. The distal femoral metaphysis was measured encompassing the whole patella with the exact bottom horizontal edge of the region of interest positioned at the top of the joint line between the femoral condyles with a height set to match that of the proximal tibia, with the proximal edge set at the upper edge of the patella. The DXA enCORE software platform automatically calculated BMD.

Participants' peak isometric torque and the mean slope of the torque-time curve between 20% and 80% of the first 200 milliseconds from muscle contraction onset (rate of torque development) were evaluated in the Healthy limb and ACL-injured limbs before ACLR and at 4mo and 6mo post-ACLR. Muscle contraction onset was defined as the point when the torque signal surpassed a 6 Nm threshold. Participants completed strength testing using a Biodex 4 isokinetic dynamometer (Biodex Medical Systems Inc., Shirley, NY, USA) with the subject's knee and hip placed at 90 degrees of flexion and secured with straps to limit extraneous movements. The dynamometer attachment arm was secured to the shank approximately 5 cm proximal to the medial malleolus. After practicing once, participants completed four subsequent

test trials and were instructed to kick and hold as hard and as quickly as they could for 5 seconds while strong verbal encouragement was given. Participants rested for 30 seconds between sets and 5 minutes rest between limbs. The torque signal was sampled at 100 Hz and filtered using a fourth-order, low-pass Butterworth zero-lag digital filter with a 24 Hz cut-off frequency. Results were analyzed with custom MATLAB code as previously described (*69*).

Mouse study methods and drug treatment

This study was approved by the Institutional Animal Care and Use Committee (protocol #2019-3241) at UK in accordance with the National Institutes of Health (NIH) *Guide for the Care and Use of Laboratory Animals*. All mice were housed under pathogen-free conditions with five or fewer mice per cage. Mice had free access to food and water. Mice were studied between 4 and 5 months of age and both males and females were used in all experiments. Bodyweights were obtained at the time of surgery and tissue collection. C57BL6/J mice were obtained from the Jackson Laboratory (Bar Harbor, ME). GDF8 knock out (GDF8KO) mice were obtained from Regeneron Pharmaceuticals (Tarrytown, NY) *(23)*. Collagen 1-enhanced green fluorescent protein (Col1-eGFP) mice were a kind gift from Dr. David Brenner *(34)*. The mice used for all experiments were randomly assigned to control or treatment groups. For drug treatments, C57BL/6J and Col1-eGFP mice were injected subcutaneously twice weekly with a human monoclonal antibody specific to GDF8/myostatin (REGN647 *(23)*; Regeneron Pharmaceuticals; 10mg/kg; GDF8 Ab) or a placebo antibody (REGN1945; Regeneron Pharmaceuticals; 10mg/kg; PLA Ab). The GDF8 Ab is a fully humanized monoclonal anti-myostatin antibody with an IgG4 Fc domain which selectively inhibits pro-, latent, and mature myostatin without binding GDF11 or Activin A (*23*). At specified time points following ACL transection or sham surgery, mice were euthanized by cervical dislocation under deep anesthesia and quadriceps muscle and whole knee joints were rapidly collected. A portion of quadriceps muscle from each limb was immediately frozen in liquid nitrogen for molecular analyses, and the remaining portion (corresponding to the vastus lateralis) was mounted in optimal cutting temperature mounting media (OCT, Tissue Tek) on cork and frozen in liquid nitrogen-cooled 2-methylbutane for

immunohistochemical analyses (C57BL/6J and GDF8-KO). For Col1-eGFP mice, to retain the fluorescent GFP label, a portion of the quadriceps was drop-fixed in 4% PFA for 24hr, equilibrated in 30% sucrose overnight and then mounted in optimal cutting temperature mounting media on cork and frozen in liquid nitrogen-cooled 2-methylbutane for immunohistochemical analyses. All samples were stored at –80°C. Whole knee joints were fixed in 4% paraformaldehyde for 48hr, washed in PBS, scanned with a micro-CT, then decalcified in 10% ethylenediaminetetraacetic acid (EDTA, pH 7.4, Sigma Aldrich) for fourteen days prior to dehydration in increasing concentrations of ethanol and embedding in paraffin.

Surgically induced PTOA mouse model

Surgical ACL transection was performed on a single limb for all mice, with the contralateral hind limb serving as an internal healthy control, similar to our prior work *(42)*. Briefly, anesthetized mice had their surgical limb shaved and prepared with povidone-iodide scrub. A 3 mm longitudinal incision was made over the distal patella to proximal tibial plateau. The joint capsule was opened by incision. The infrapatellar fat pad over the intercondylar area was dissected and the patellar tendon gently dislocated laterally to provide greater exposure of the femoral-tibial joint and allow visualization of the ACL in the intercondylar region. The ACL was completely transected with scalpel under visualization with a stereo dissecting microscope, and damage to the posterior cruciate ligament was avoided. The incision was closed with a suture, and the mouse was placed in a clean cage over a heat pad and monitored for recovery. Transection was confirmed with a positive anterior drawer. Mice were monitored regularly throughout the post-surgical time course for signs of pain and inflammation and to confirm proper healing of the incision site. To control for the effects of surgery on study outcomes, we performed sham surgery on subsets of mice (n=8 at 7 days post sham surgery, and n=4 at 28 days post sham surgery). Sham surgery consisted of the same pre-operative care, longitudinal incision and opening of the joint capsule with no disturbance of the ACL, followed by the same suture protocol to close the incision.

Unilateral limb immobilization mouse model

Unilateral hindlimb immobilization was performed in mice with modifications as previously described *(31)*. To make a splint, a 4 (length) x 1.7cm (diameter) cast was 3D printed and composed of polylactic acid (#B0B96ZVRLP, Creality 3D, Shenzhen, China). The splint was applied while mice were under isoflurane anesthesia (2-4%), with a single limb placed in the cast and fastened at two points by wrapping the limb together with the adhesive side of 5 x 1.1cm Velcro loop (Velcro #90198, Velcro USA, Inc., Manchester, NH). The Velcro loops were used to secure the 1) dorsal aspect of the mouse foot, and 2) the tibia just distal to the patella to the cast, with both points in direct contact with the fabric side of the Velcro loop. The immobilized limb was maintained with the knee in an extended position and the ankle in a plantar-flexed position for one week. Contralateral quadriceps muscles that were not placed in the splint served as controls. A subset of mice underwent combined surgical ACL transection (ACLT) plus unilateral hindlimb immobilization, where a single limb underwent ACLT followed by immediate unilateral immobilization of the same limb. The ACLT + immobilized limb was maintained with the knee in an extended position and the ankle in a plantar-flexed position for one week. Contralateral quadriceps muscles that did not undergo ACLT plus unilateral hindlimb immobilization served as controls.

In vivo tetanic torque and RTD

Muscle isometric mechanics were assessed as described previously *(70)* on the quadriceps muscle using a Whole Mouse Test System (Cat #1300A; Aurora Scientific, ON, Canada). Mice were anesthetized with 2.5% isoflurane, and their hind limb was fixed to a platform (Aurora Scientific 809c in vivo testing apparatus) heated to 37°C (Anova Industries Model 10 water circulator). The leg was then mounted into a clamp to hold it static with the lower hind limb placed inside the knee extension apparatus connected to the isometric force transducer (Aurora Scientific), with the tibia lightly resting against the knee extension apparatus (confirmed by

negative Force Out reading). The motor point of the quadriceps was identified and stimulated by bipolar silver electrode anterior to the hip joint. With an Aurora Physiology system (Model 6650LR Force Transducer, Dual Mode lever System, Hi power Bi-Phase Stimulator, Signal Interface, and software: Dynamic Muscle Control v5.5 and Dynamic muscle Analysis version 5.3), the proper maximal current used to stimulate muscle contraction was determined by eliciting a twitch (pulse duration 0.2 s) with the stimulus starting at 50 mA. A torque-frequency curve was then conducted to find the maximum torque output (10, 40, 100, 150, 180, 200Hz with 2 min rest periods between each contraction). Quadriceps torque was assessed at various time-points post-ACLT (7, 14, or 28 days). Rate of torque development (RTD) was assessed with a custom Matlab script. The frequency that elicited maximal torque output for each mouse was used for RTD calculation. The linear torque-time slope was calculated with a least squares regression fit during twentieth to eightieth percentile of peak tetanic torque. RTD values were also measured controlling for peak tetanic torque. The time torque curve was normalized to the peak value, and the linear torque-time slope was calculated with a least squares regression fit during twentieth to eightieth percentile of peak tetanic torque to reflect peak torque normalized RTD.

Immunohistochemistry

Frozen human and mouse quadriceps tissue was sectioned (7µm thickness) on a cryostat and air dried for one hour. Laminin immunohistochemical staining was performed with sections fixed in acetone for 3 minutes at -20°C, brought to room temperature, washed in PBS and then incubated overnight in and rabbit polyclonal anti-laminin (1:100, #L9393, Sigma, St. Louis, MO) in 1% bovine serum albumin *(72)*. The following day, slides were incubated in goat anti rabbit AF350 (#A21068, Life Technologies) and then cover slipped.

pSMAD3 and laminin/dystrophin. Slides were fixed in 4% paraformaldehyde followed by epitope retrieval using sodium citrate (10 mM, pH 6.5) at 92°C for 20 min. Slides were incubated overnight in anti-p-SMAD3 (1:75, #9520; Cell Signaling) and anti-dystrophin (1:50, #VP D505;

Vector Laboratories, Burlingame, CA) or laminin (1:100; MA5-24,656, Invitrogen). The following day, slides were incubated in goat anti-rabbit AF555 (1:500, #A-21429) and goat anti-mouse AF488 (1:500, #A-21121), both from ThermoFisher, then co-stained with DAPI and mounted with fluorescent mounting media.

Collagen 1 and 4 and GFP. Slides were fixed in 4% paraformaldehyde followed by epitope retrieval using sodium citrate (10 mM, pH 6.5) at 92°C for 20 min. Slides were incubated overnight in anti-GFP (1:100, Abcam #ab13970) and anti-collagen 1 and 4 (both 1:100, Abcam, #ab34710 and #ab6586). The following day, slides were incubated in goat anti-rabbit AF555 (1:500, #A-21429) and goat anti-chicken AF488 (1:500, #A-11039), both from ThermoFisher, then co-stained with DAPI and mounted with fluorescent mounting media.

Actin and GFP. Using fine forceps under a stereo microscope, single muscle fibers were mechanically dissected from fixed quadriceps in Col1-eGFP mice prior to incubation in AF594 Phalloidin (1:200, #A12381) for 2hr. Fibers were then incubated co-stained with DAPI and mounted with fluorescent mounting media. Col1-eGFP+ cells were fixed in 4% paraformaldehyde prior to permeabilization with 0.5% Triton-X100. Cells were incubated in AF594 Phalloidin (1:200, #A12381) for 2hr and then co-stained with DAPI.

Periostin and GFP. Slides were fixed in 4% paraformaldehyde followed by epitope retrieval using sodium citrate (10 mM, pH 6.5) at 92°C for 20 min. Slides were incubated overnight in anti-GFP (1:100, Abcam #ab13970) and anti-periostin (1:100, Abcam, #ab14041). The following day, slides were incubated in goat anti-rabbit AF555 (1:500, #A-21429), goat anti-chicken AF488 (1:500, #A-11039), and wheat germ agglutinin AF647 (1:50, #W32466), all from ThermoFisher, then co-stained with DAPI and mounted with fluorescent mounting media.

Tcf7l2 and GFP. Slides were fixed in 4% paraformaldehyde followed by epitope retrieval using sodium citrate (10 mM, pH 6.5) at 92°C for 20 min. Slides were incubated overnight in anti-GFP (1:100, Abcam #ab13970) and anti-Tcf7l2 (1:100, Cell Signaling, #2569). The following day, slides were incubated in Biotin-SP Goat anti-Rabbit (1:1,000, #111-065-003, Jackson Immuno Research, West Grove, PA), and goat anti-chicken AF488 (1:500, #A-11039) and wheat germ

agglutinin AF647 (1:50, #W32466), both from ThermoFisher, followed by incubation in streptavidin conjugated to horseradish peroxidase and amplification via tyramide signal amplification AF555 (AF555 Tyramide SuperBoost Kit, streptavidin, #B40933, ThermoFisher) prior to co-staining with DAPI.

Alpha smooth muscle actin (αSMA) and GFP. Slides were fixed in 4% paraformaldehyde followed by epitope retrieval using sodium citrate (10 mM, pH 6.5) at 92°C for 20 min. Slides were incubated overnight in anti-GFP (1:100, Abcam #ab13970) and anti-αSMA (1:100, Santa Cruz, #sc-53015). The following day, slides were incubated in goat anti-mouse IgG2B AF555 (1:500, #A-21147), goat anti-chicken AF488 (1:500, #A-11039), and wheat germ agglutinin AF647 (1:50, #W32466), all from ThermoFisher, then co-stained with DAPI and mounted with fluorescent mounting media.

αSMA, Periostin, and GFP. Slides were fixed in 4% paraformaldehyde followed by epitope retrieval using sodium citrate (10 mM, pH 6.5) at 92°C for 20 min. Slides were incubated overnight in anti-GFP (1:100, Abcam #ab13970), anti-periostin (1:100, Abcam, #ab14041), and anti-αSMA (1:100, Santa Cruz, #sc-53015). The following day, slides were incubated in goat anti-mouse IgG2B AF555 (1:500, #A-21147), goat anti-rabbit AF647 (1:500, #A-21245), and goat anti-chicken AF488 (1:500, #A-11039), all from ThermoFisher, then co-stained with DAPI and mounted with fluorescent mounting media.

Histology

For collagen staining, quadriceps slides were fixed 1hr at 56°C in Bouin's fixative then incubated in picro-sirius red, washed in 0.5% acetic acid, dehydrated, equilibrated with xylenes and then mounted with cytoseal XYL (Thermo Scientific). Following paraffin embedding, whole knees were sectioned at 4μm thickness in the coronal plane. Sections from the center of the joint were stained with Safranin O/Fast Green for the evaluation of cartilage damage. Briefly, 1.0% solution of Safranin O and 0.2% solution of Fast Green were applied to sections for five and 1 minutes, respectively, each at room temperature prior to dehydration and mounting.

Image acquisition and analysis

Images were captured at x100-200 magnification at room temperature using a Zeiss upright microscope (AxioImager M2; Zeiss, Oberkochen, Germany). CSA (μm^2) was directly assessed using MyoVision automated software *(73)*. Image analysis was performed in a blinded manner to drug and injury status using Image J Fiji or Zen software (v3.1, Zeiss). Images appearing in Fig. 3A-B were captured at x200 total magnification on a Nikon A1R inverted confocal microscope.

Picro-sirius red staining was quantified to measure collagen content using Image J Fiji software as previously described *(32)*. The area of picro-sirius red+ collagen was normalized to the total muscle area (mm^2). Two blinded raters assessed pico-sirius red+ collagen from all GDF8 Ab- and PLA Ab-treated mice and the intraclass correlation coefficient (ICC) was calculated to determine reliability of the data. ICC estimate and 95% confidence intervals was calculated using SPSS statistical software (IBM Corp. IBM SPSS Statistics for Windows, Version 25. Armonk, NY) based on a mean-rating, consistency, 2-way mixed-effects model with 2 raters across 98 total images. The ICC for pico-sirius red+ collagen was 0.881, 95% CI (0.822, 0.920), indicating excellent reliability and reproducibility of the data.

To evaluate collagen organization, picro-sirius red-stained muscle sections were also viewed under circularly polarized light *(39)*. Under quarter wavelength polarized light, whole muscle images were obtained as with light microscopy to quantify densely packed (red), intermediate (yellow), and loosely packed (green) collagen using the ImageJ FIJI Color Pixel Counter and expressed relative to total muscle area and to the area of total collagen/picro-sirius red *(74, 75)*. Col1-eGFP+ cells were manually counted and normalized to the total muscle area (mm^2). Collagen 1 and 4 area was normalized to the total muscle area (mm^2). Periostin+; Col1-GFP+ cells were manually counted and normalized to total Col1-eGFP+ cells (%). Tcf7l2+; Col1-GFP+ cells were manually counted and normalized to total Col1-eGFP+ cells (%). αSMA+, Col1-GFP+ cells were manually counted and normalized to total Col1-eGFP+ cells (%).

Immunocytochemical analysis of Col1-eGFP+ cells was performed using an automated algorithm (Zeiss Zen 3.1) to identify all Col1-eGFP+ cells (GFP+/DAPI+) so that relative intensity of the specific antibody fluorescent signals (periostin, transcription factor 7-like 2 [Tcf7l2], platelet derived growth factor receptor alpha [PDGFRα]) could be determined on a per-cell basis. The algorithm automatically identified Col1-eGFP+ cells (GFP+/DAPI+), and then determined the fluorescent signal intensity for periostin, Tcf7l2 or PDGFRα. A minimum of 500 cells were analyzed per limb (control and ACLT) from each mouse and mean integrated density was averaged across cells.

Histological assessment of safranin O-stained knee joints was performed on the medial tibial plateau through blinded graded observations following the Osteoarthritis Research Society International (OARSI) scoring system *(49)*. To measure the thicknesses of articular cartilage, uncalcified cartilage was defined as the area from articular surface to tide mark and calcified cartilage was defined as the area from tide mark to cement line, similar to our prior work *(76)*. Thickness of these regions was manually assessed by a single blinded observer at three separate locations along the medial tibial plateau (lateral, midline and medial) with the final measurement representing an average of these three thickness measures.

Micro-computed tomography (microCT) analysis

Prior to decalcification, whole fixed knees from Control and ACLT limbs were scanned using a Skyscan 1276 micro-CT (Bruker, Billerica, MA). Knees were scanned 28d post-ACLT in both GDF8 Ab- and PLA Ab-treated mice as well as GDF8KO mice. Whole knees were scanned with a voxel size of 9µm for analysis of 3-dimensional bone structure with the following scanning parameters used for both limbs: energy = 50 kV, intensity = 200 µA, integration time = 300 ms, and 0.25 mm Al beam hardening filter. Each microCT image was reconstructed using the software provided by the manufacturer (NRecon, Bruker; ring artifact correction = 4) using a polynomial beam hardening correction function based on the scans of two phantoms at 0.25 and 0.75 g/cm^3 as we have described previously *(71)*. We focused analysis on the proximal tibia

as loss in BMD in the proximal tibia in patients showed the strongest correlation with GDF8 levels. Trabecular bone regions of interest (ROIs) were identified by manually contouring the trabecular bone between the subchondral plate and growth plate in 2D cross-sections in microCT images every 5 slices. The following parameters were evaluated: trabecular bone volume fraction (BV/TV), bone mineral density (BMD), trabecular thickness (Tb.Th), trabecular separation (Tb.Sp), trabecular number (Tb.N), trabecular porosity (Tb.Po), structure model index (SMI) and connectivity density (Conn.D). For the cortical subchondral plate analysis, one ROI was identified in the medial portion of the tibial plateau, after removing the underlying trabecular bone. The ROI was centered in the medial condyle and was identified with the same size (500 µm x 500 µm) and same spatial position for all images. The following parameters were evaluated: subchondral bone cortical plate thickness (Sb.Pl.Th) and subchondral bone cortical plate porosity (Sb.Po).

Fluorescent activated cell sorting (FACS) of Col1-eGFP+ cells and immunocytochemical analysis

Col1-eGFP+ cells were isolated from the quadriceps muscles of mice 7 days after ACLT by enzymatic digestion and FACS via a modified protocol *(78)*. Entire quadriceps muscles were dissected from the hind limb and mechanically minced under sterile conditions. A single cell suspension was created by incubating the freshly minced muscle at 37°C in 800U/ml Collagenase II (Worthington Biochemical, Lakewood, NJ), followed by centrifugation (500g for 5 minutes) and an additional incubation of the resultant pellet in Collagenase II and 11U/ml Dispase (Life Technologies). The suspension was centrifuged again (500g for 5 minutes), and the resultant pellet was resuspended and filtered through a 40µm cell strainer. The filtered sample was centrifuged (500g for 5 minutes), and the remaining cell pellet was resuspended; DAPI was added to the cell suspension as a viability marker, and the cell suspension was then sorted using an iCyt FACS machine (Sony Biotechnology, Champaign, IL, USA)—along with appropriate negative controls for gating (muscle cell suspension from C57BL/6 wild type mouse). Appropriate isotype specific controls were used to validate antibody specificity, and

dead cells were gated out using DAPI. GFP+/DAPI- cells were sorted directly into 4% paraformaldehyde for further analysis. A portion of fixed GFP+/DAPI- cells were spun onto glass microscope slides using a Cytospin 4 (Thermo Scientific, 750 rpm for 2 minutes) for immunocytochemical analysis. A portion of GFP+ cells from only the ACLT limbs were centrifuged and resuspended in 2% BSA prior to incubation in the following antibodies: Anti-Integrin α7- Phycoerythrin (PE) (to identify satellite cells; 1:50; # K0046-5, MBL, Woburn, MA) and anti-stem cell antigen-1 (Sca-1)- Allophycocyanin (APC) (to identify fibro/adipogenic progenitor cells (FAPs), 1:50, #122512, BioLegend, San Diego, CA). Fixed cells were incubated on ice for 40min with primary antibodies, washed, centrifuged and resuspended in 2% BSA for FACS.

GFP+ cells were sorted using a iCyt FACS machine equipped with five lasers (355 nm, 405nm, 488nm, 561nm and 642nm) with GFP+ cells sorted with the following strategy: GFP+/Sca-1+/α7-, GFP+/Sca-1-/α7+, and GFP+/Sca-1-/α7- using WinList (Verity Software House, Topsham, ME). The fluorescent antibodies were detected at the following wavelengths: 455 nm (UV/DAPI), 525nm (GFP), 585nm (PE), and 665nm (APC). Very few GFP+ satellite cells (GFP+/Sca-1-/α7+) cells were detected (<5% of total GFP+ cells), and GFP+ satellite cells were sorted into a collection vial filled with 4% paraformaldehyde for immunocytochemical verification. The majority of GFP+ cells were sorted as positive or negative for Sca-1 (FAPs or not FAPs). GFP+ FAPs (GFP+/Sca-1+/α7-) were sorted into a collection vial filled with 4% paraformaldehyde for immunocytochemical verification. GFP+ cells that were negative for Sca-1 and α7 integrin (GFP+/Sca-1-/α7-) were sorted into vials filled with 4% paraformaldehyde. These GFP+ cells were permeabilized in a buffer consisting of 2% bovine serum albumin and 0.1% Triton X-100 for 10 min prior to incubation in the following antibody: anti-periostin-PE (1:50, #sc-398631 PE, Santa Cruz Biotechnology, Santa Cruz, CA). Fixed cells were incubated on ice for 40min with primary antibody, washed, centrifuged and resuspended in 2% BSA for FACS. GFP+ cells sorted with the following strategy: GFP+/periostin+ and GFP+/periostin-. All data were analyzed with FlowJo software (version 10.7.2, FlowJo, Ashland, OR).

GFP+/DAPI- cells were sorted directly into 4% paraformaldehyde and spun onto glass microscope slides using a Cytospin 4 (Thermo Scientific, 750 rpm for 2 minutes). Slides were incubated in primary antibodies against periostin (1:100, #ab14041, Abcam), Tcf7l2 (1:100, #2569, Cell Signaling), or PDGFRα (1:100, #AF1062, R&D Systems) overnight in 2% bovine serum albumin. The following day, slides were incubated in anti-rabbit secondary antibodies (Goat anti-rabbit AF555, 1:500, #A-21428 [persiotin and Tcf7l2], ThermoFisher or donkey anti-goat AF555, 1:500, #A-21432 [PDGFRα], ThermoFisher) prior to co-staining with DAPI. GFP+/Sca-1+ cells were spun onto glass microscope slides, incubated in primary antibody against PDGFRα (1:100, #AF1062) overnight in 2% bovine serum albumin. The following day, slides were incubated in donkey anti-goat AF555 (1:500, #A-21432, ThermoFisher) prior to co-staining with DAPI. GFP+/α7+ cells were spun onto glass microscope slides, incubated in primary antibody against Pax7 (1:100, #Pax7, Developmental Studies Hybridoma Bank, Iowa City, IA) overnight in 2% bovine serum albumin. The following day, slides were incubated for 90 min in a goat anti-mouse biotinylated secondary antibody (1:1,000; #115–065–205, Jackson Immuno Research, West Grove, PA), followed by incubation in streptavidin conjugated to horseradish peroxidase and amplification via tyramide signal amplification AF647 (AF647 Tyramide SuperBoost Kit, streptavidin, #B40936, ThermoFisher) prior to co-staining with DAPI.

Immunoblotting

Immunoblot experiments were performed as previously described *(42)*. A portion of the mouse quadriceps and human vastus lateralis were homogenized by bead milling with 0.5-mm zinc oxide beads (Bullet Blender; Next Advance) in ice-cold buffer with phosphatase and protease inhibitor tablets. Protein concentration of all fractions was assessed using the Bradford Assay. Fifty micrograms of protein was then diluted 1:1 in sample buffer (2x Laemmli Sample Buffer, Bio-Rad, #1610737EDU), boiled at 100°C for three minutes, and loaded onto a 4-20% gradient gel (Bio-Rad, #5678094, TGX Stain-Free Protein Gels) and ran electrophoretically under non-reducing conditions at 150V until the protein bands reached the bottom of the gel. An internal control was loaded onto each membrane for comparison across membranes. Following

electrophoretic separation, stain free gels were activated on a ChemiDoc MP for 1 minute under UV illumination. Protein was then transferred to PVDF membranes (Bio-Rad, #1620175) at 50V for one hour in ice cold transfer buffer. Equivalent loading and efficient transfer was verified using stain free imaging of the PVDF membranes. Membranes were subsequently blocked for one hour at room temperature in a 2% bovine serum albumin solution and then incubated in primary antibody overnight with gentle agitation: anti-GDF8 (#ab203076; Abcam), anti-phosphorylated SMAD3 (Ser423/425) (#9520; Cell Signaling), or anti-SMAD3 (#56783; Cell Signaling), all 1:1,000. A horse radish peroxidase (HRP)-linked donkey anti-rabbit IgG secondary antibody (#GENA934; Sigma Aldrich) was used followed by ECL detection. Chemiluminescent solution (#32132, Thermo Scientific) was applied to each blot and incubated for 5 minutes. Membranes were imaged using optical density measurements on a ChemiDoc MP (Bio-Rad) and quantified using Image Lab software (Image Lab, Bio-Rad). Density values obtained from quantification of bands of interest were normalized to total protein via stain-free gels. Comparison across membranes was further considered by normalizing all density values to an internal loading control present on all membranes.

GDF8 ELISA

Human serum, human vastus lateralis biopsy homogenate and mouse quadriceps homogenate were analyzed in duplicate for each sample using the GDF8/myostatin ELISA kit (#ab267656, Abcam) following manufacturer’s instructions.

Hydroxyproline assay

To determine total collagen content, hydroxyproline was measured with a modified protocol using a commercially available Hydroxyproline Assay Kit *(37)* (MAK008, Millipore Sigma, Darmstadt, Germany). Human vastus lateralis muscle samples and mouse quadriceps muscle samples were homogenized by bead milling with 0.5-mm zinc oxide beads (see above; Bullet Blender; Next Advance) in ice-cold buffer with phosphatase and protease inhibitor tablets.

Following centrifugation, the supernatant was removed for immunoblot analysis, and the pellet containing the collagen fraction was weighed and stored at -80°C. The pellet was homogenized in double-distilled water (volume equal to 10x pellet weight; i.e. 100μl double-distilled water for 10mg pellet weight). Following homogenization, the sample was vortexed and a volume of 12M HCl equal to the water was added to hydrolyze the sample overnight at 105°C. The next day after hydrolysis, the sample/hydrolysate was vortexed and 20μl was loaded in duplicate onto a 96-well plate along with hydroxyproline standard included in the Hydroxyproline Assay Kit; samples and standards were dried overnight in an oven at 60°C. Chloramine T/Oxidation Buffer included in the Hydroxyproline Assay Kit was added to each well followed by incubation at room temperature for 5 minutes. Diluted p–dimethylaminobenzaldehyde (DMAB) Reagent was then added to each well, and the microplate was incubated at 60°C for 90 minutes. Absorbance was measured at 595nm, and hydroxyproline content was calculated using a standard curve and normalized to the loaded sample volume (20μl).

Pyridinium crosslink assay

Pyridinium (PYD) crosslink (pyridinoline and deoxypyridinoline) concentration was determined by enzyme-linked immunoassay Metra PYD EIA kit (Quidel Corporation, San Diego, CA), as previously described using skeletal muscle hydrolysate *(79)*. All standards and samples were analyzed in duplicate, and a four-parameter logistic curve was used to calculate sample PYD concentration. Data are expressed as nmol pyridinoline (PYD)/L and relative to total collagen measured by hydroxyproline content [PYD (nmol/L)/HOP (µg/µl)].

RNA isolation – mouse quadriceps

Approximately 25mg of quadriceps muscle from both Control and ACLT limbs in both GDF8 Ab and PLA Ab-treated mice 7d post-ACLT were homogenized (n: Control = 5, ACLT = 5 for both GDF8 Ab and PLA Ab; samples were not pooled) in TRI Reagent (#R2050-1-200, Zymo

Research). RNA was isolated using the Direct-zol RNA Miniprep kit (#R2051, Zymo Research) per manufacturer's instructions. RNA concentration and quality (RIN > 8.5, average RIN: 9.3) was assessed with an RNA Nano chip kit on a bioanalyzer by the University of Kentucky Genomics Core Laboratory. Six hundred nanograms of total RNA was sent to Novogene for library construction and sequencing on an Illumina HiSeq 4000 system using a paired-end 150 bp dual-indexing protocol. Raw FASTQ files underwent pre-alignment quality control, then were aligned using STAR aligner (reference genome: GRCm38/mm10) in Partek Flow, and analyzed to obtain differential gene expression using DESeq2 (minimum read cutoff of 50). Differential gene expression was calculated comparing ACLT and Control limbs for both GDF8 Ab and PLA Ab. Additional comparisons were performed in the Control limb between GDF8 Ab and PLA Ab, as well as in the ACLT limb between GDF8 Ab and PLA Ab. Raw p-values were adjusted for multiple testing using the Benjamini-Hochberg false discovery rate (FDR) step-up method. REACTOME Pathway over-representation analysis was performed using gProfiler with non-ordered query and up-or-downregulated genes with FDR < 0.05. Quadriceps RNA-sequencing data are deposited in Gene Expression Omnibus: GSE230708.

RNA isolation – mouse cartilage

Articular cartilage from both Control and ACLT limbs in both GDF8 Ab and PLA Ab-treated mice 7d post-ACLT was collected from the knee joint by scraping approximately 1 mm of tissue from the end of both the proximal tibia and distal femur using a surgical scalpel, as previously described *(77)*. To ensure sufficient quality and quantity of RNA, articular cartilage was quickly flash frozen in liquid nitrogen and stored at -80° C until the tissue was pooled from 4 mice to constitute each sample (i.e. articular cartilage was pooled across 4 mice to constitute 1 biological replicate; 2 biological replicates were analyzed for each condition (injury and drug) reflective of 8 total mice per condition). Pooled tissue samples were homogenized in TRI Reagent (#R2050-1-200, Zymo Research) using ceramic beads and a benchtop bead homogenizer (Bullet Blender, Next Advance). Following homogenization, RNA was isolated using the Direct-zol RNA Microprep kit (#R2063, Zymo Research) per manufacturer's

instructions. RNA concentration and quality (RIN > 6.0, average RIN: 7.1) were measured by an RNA Nano chip kit on a bioanalyzer by the University of Kentucky Genomics Core Laboratory. Three hundred nanograms of total RNA was sent to Novogene for library construction and sequencing on an Illumina HiSeq 4000 system using a paired-end 150 bp dual-indexing protocol. Raw FASTQ files underwent pre-alignment quality control, then were aligned using STAR aligner (reference genome: GRCm38/mm10) in Partek Flow, and analyzed to obtain differential gene expression using DESeq2 (minimum read cutoff of 50). Differential gene expression was calculated comparing ACLT and Control limbs for both GDF8 Ab and PLA Ab. Additional comparisons were performed in the Control limb between GDF8 Ab and PLA Ab, as well as in the ACLT limb between GDF8 Ab and PLA Ab. Raw p-values were adjusted for multiple testing using the Benjamini-Hochberg false discovery rate (FDR) step-up method. REACTOME Pathway over-representation analysis was performed using gProfiler with non-ordered query and up-or-downregulated genes with FDR < 0.05. Cartilage RNA-sequencing data are deposited in Gene Expression Omnibus: GSE230736.

Statistics

For human studies, statistical analysis was performed on continuous data using mixed models with no sphericity assumption to account for repeated observations within the same subject. Dunnett's multiple comparison tests were performed with individual variances computed for each comparison to specifically compare post-injury data with the Healthy limb value. Pearson correlation coefficients were calculated in Figure 1J by determining the percent difference in quadriceps GDF8 between the Healthy limb and the ACL-injured limb and comparing those values to percent difference between the Healthy limb and ACL-injured limb 6 months post-ACLR (peak isometric torque, rate of torque development, peak isokinetic torque, proximal tibial metaphysis BMD and distal femoral metaphysis BMD) or ACL-injured limb 4 months post-ACLR (quadriceps fiber cross-sectional area). Individual scatter plots are shown in Supplemental Figure 3. Pearson correlation coefficients were calculated in Supplemental Figure 1E comparing quadriceps GDF8 abundance in the ACL-injured limb to serum GDF8 levels prior to ACLR.

For mouse studies, we fit a series of mixed models with drug as a fixed effect (GDF8 Ab vs. PLA Ab) and a random effect modeling correlation between limbs within each mouse (comparing ACLT and Control). Separate comparisons were performed within each distinct time point (we did not compare across time points; e.g. 7, 14 or 28d post-ACLT were each analyzed separately). If significant interactions were identified (drug x injury $p<0.05$), Šidák's multiple comparison post hoc testing was performed. Pairwise comparisons of interest via Šidák's are denoted in figures with asterisks defined for given p-value references. Drug x injury interaction p-values are provided for all comparisons in the respective figure legends. For instances where two independent samples were compared, we performed two-sample t-tests. Sample sizes were estimated from prior studies in the lab (*42*). No data points were excluded from any mouse or human sample. Statistical testing is denoted in all figure legends. Data are presented as mean ± SD with individual data points overlaid on bar charts. No assumption violations were observed for parametric statistical testing for human and mouse data; these assumptions include distributional and equal variance. P-values less than 0.05 were considered statistically significant. All analyses were performed with GraphPad Prism 9.0.2.

Supplemental Figures

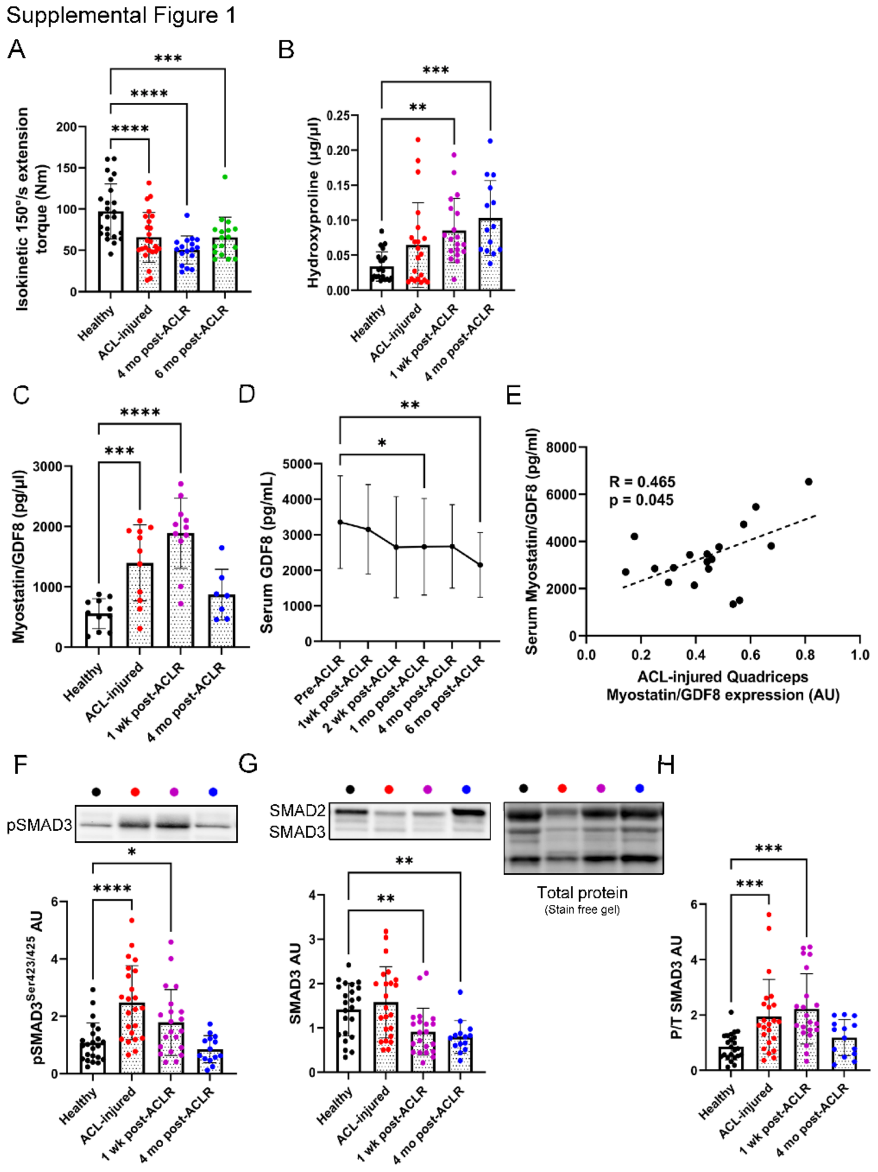

Supplemental Figure 1. Elevated muscle and systemic GDF8/myostatin following ACL injury. A. Knee extension peak isokinetic torque is reduced in the injured limb after ACL injury and is not resolved by traditional rehabilitation. B. Quadriceps hydroxyproline content is elevated following ACL reconstruction. C. Myostatin/GDF8 expression is elevated in quadriceps muscle after ACL injury. D. Serum Myostatin/GDF8 levels are most elevated following the initial ACL injury. E. Quadriceps Myostatin/GDF8 expression in the ACL-injured limb is positively correlated with serum Myostatin/GDF8 levels collected at the same time point. F. phospho-SMAD3 expression is elevated in quadriceps muscle after ACL injury and remains elevated following reconstruction. G. SMAD3 protein expression is lower in the ACL-injured quadriceps following reconstruction and remains depressed 4 months following ACLR. H. pSMAD3 normalized to total SMAD3 shows induction of SMAD3 following ACL injury and reconstruction. N=23 (A, B, F, G, H), 18 (D, E), 11 (C). *p<0.05 vs. Healthy, **p<0.01 vs. Healthy, ***p<0.005 vs. Healthy, ****p<0.001 vs. Healthy via mixed effects model and Dunnett's correction for multiple comparisons.

Supplemental Figure 2

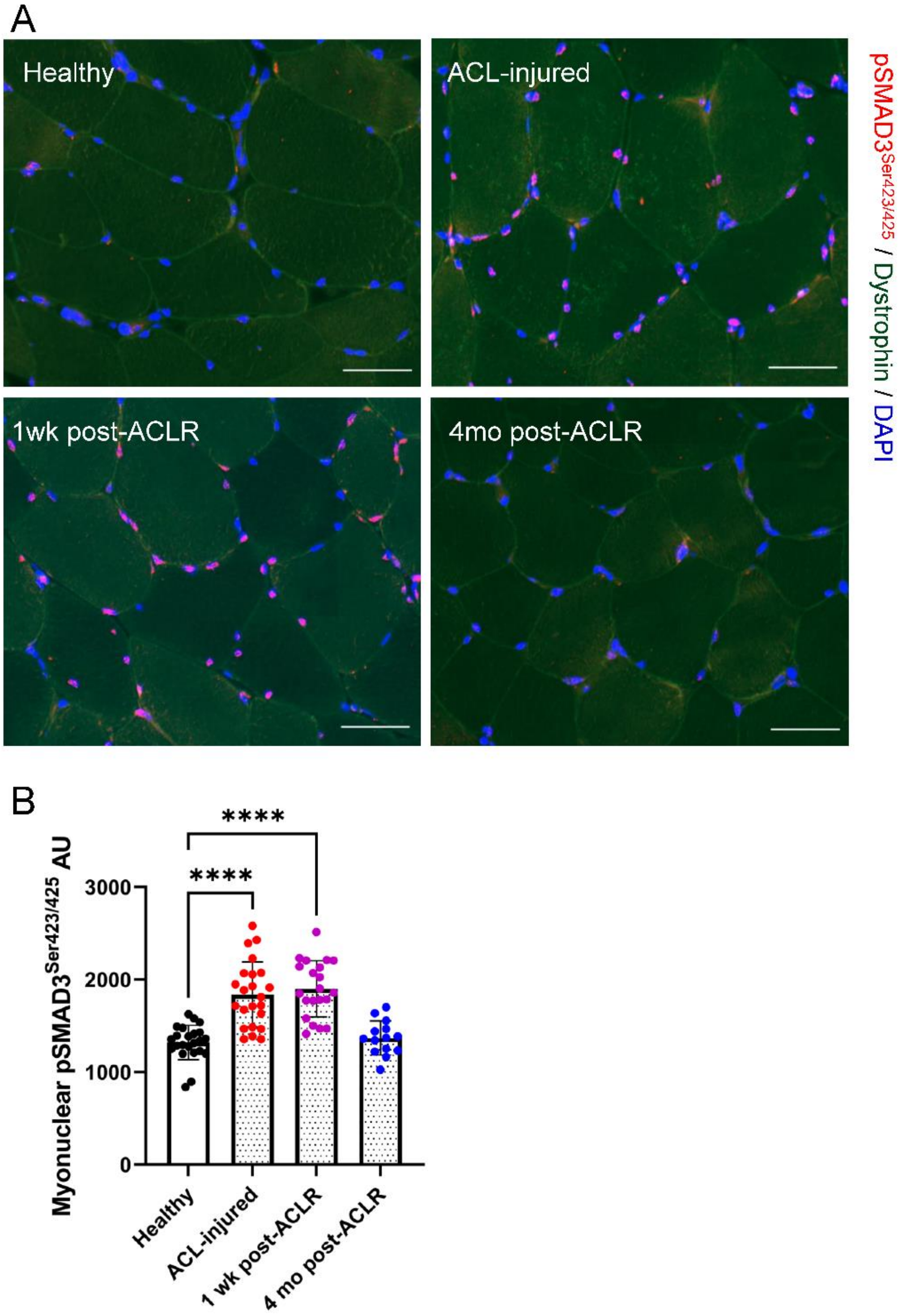

Supplemental Figure 2. ACL-injury and ACL reconstruction induce myonuclear phospho-SMAD3. A. Representative immunohistochemical images of dystrophin (green), phospho-SMAD3 (Ser423/425, red) and DAPI (blue), scale bar=50µm. B) Myonuclear phospho-SMAD3 is elevated after ACL injury and ACLR. N=23. ****$p<0.001$ vs. Healthy via mixed effects model and Dunnett's correction for multiple comparisons.

Supplemental Figure 3

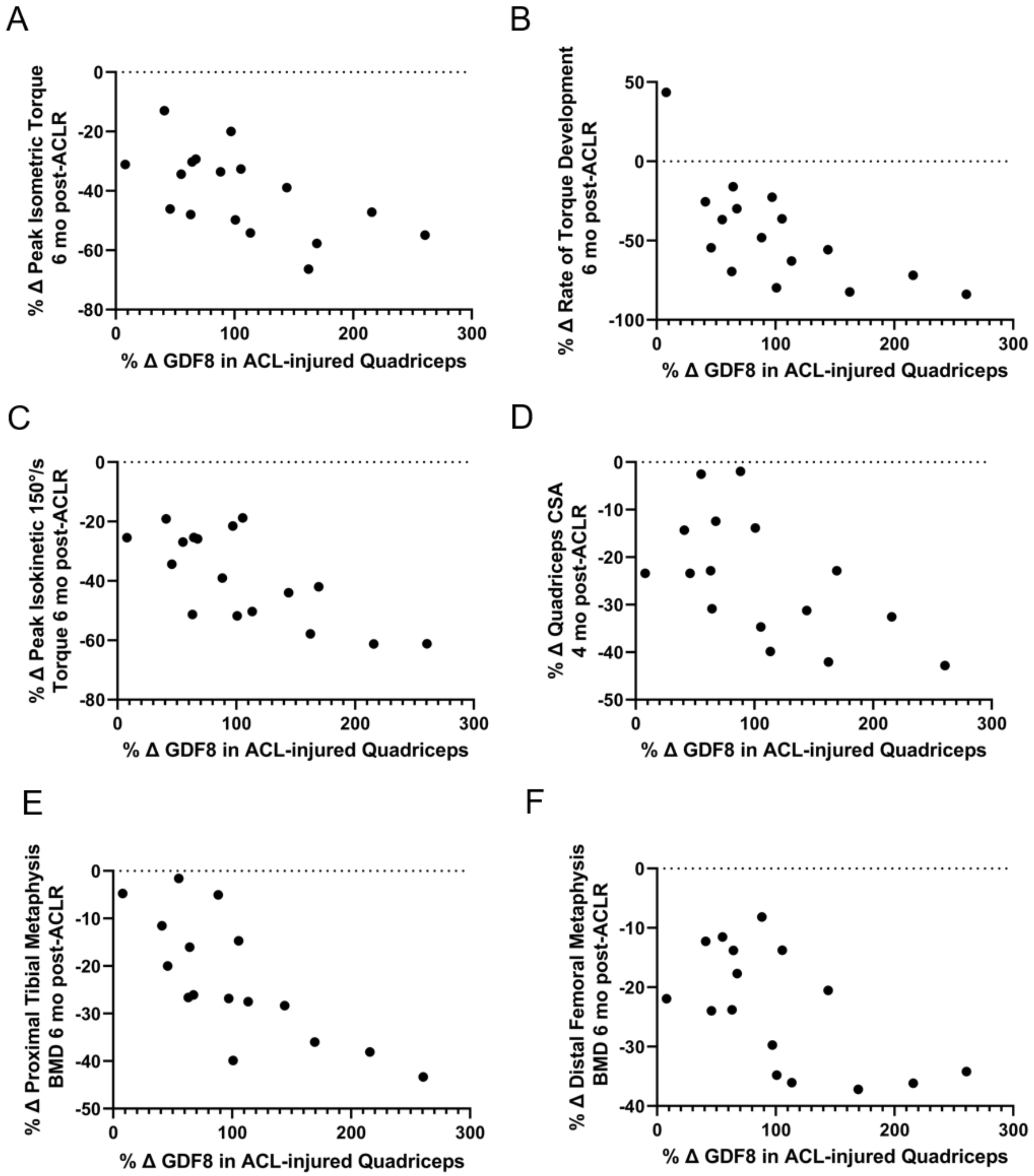

<u>Supplemental Figure 3</u>. Scatterplots depicting the percent change in myostatin/GDF8 in the ACL-injured limb and the percent change in A. quadriceps peak isometric torque 6 months following ACLR, B. quadriceps rate of torque development 6 months following ACLR, C. quadriceps peak isokinetic torque 6 months following ACLR, D. quadriceps muscle fiber cross-sectional area 4 months following ACLR, E. proximal tibial metaphysis bone mineral density 6 months following ACLR, F. distal femoral metaphysis bone mineral density 6 months following ACLR. N=17 (A, C), 16 (B, D, E, F).

Supplemental Figure 4

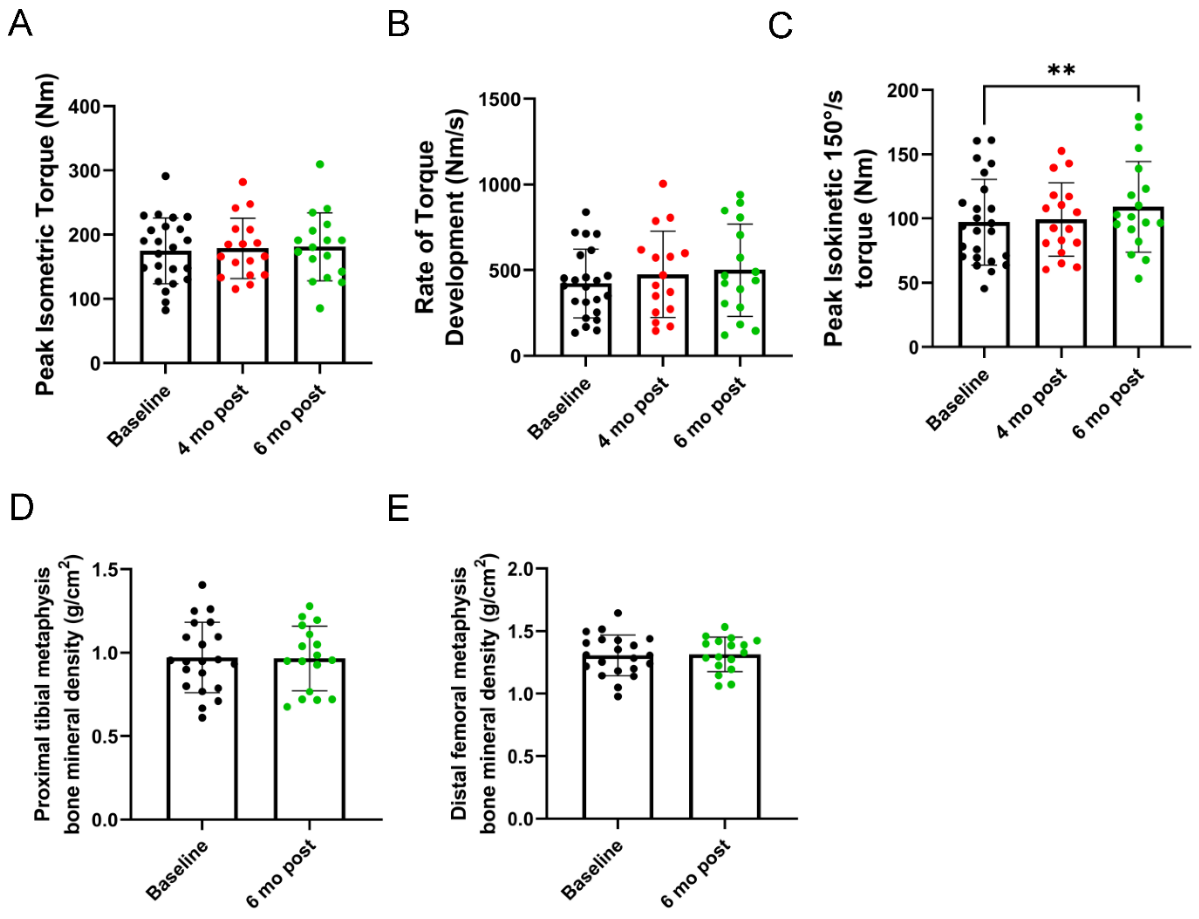

Supplemental Figure 4. Repeated assessment of quadriceps strength and periarticular bone mineral density in the healthy, non-involved limb show minimal change across the study time period. A. Knee extension peak isometric torque. B. Knee extension rate of torque development. C. Knee extension peak isokinetic torque, D. Bone mineral density in the proximal tibial metaphysis, E. Bone mineral density in the distal femoral metaphysis. **$p<0.01$ vs. Baseline via mixed effects model and Dunnett's correction for multiple comparisons. N=23 (A-C), 21 (D-E).

Supplemental Figure 5

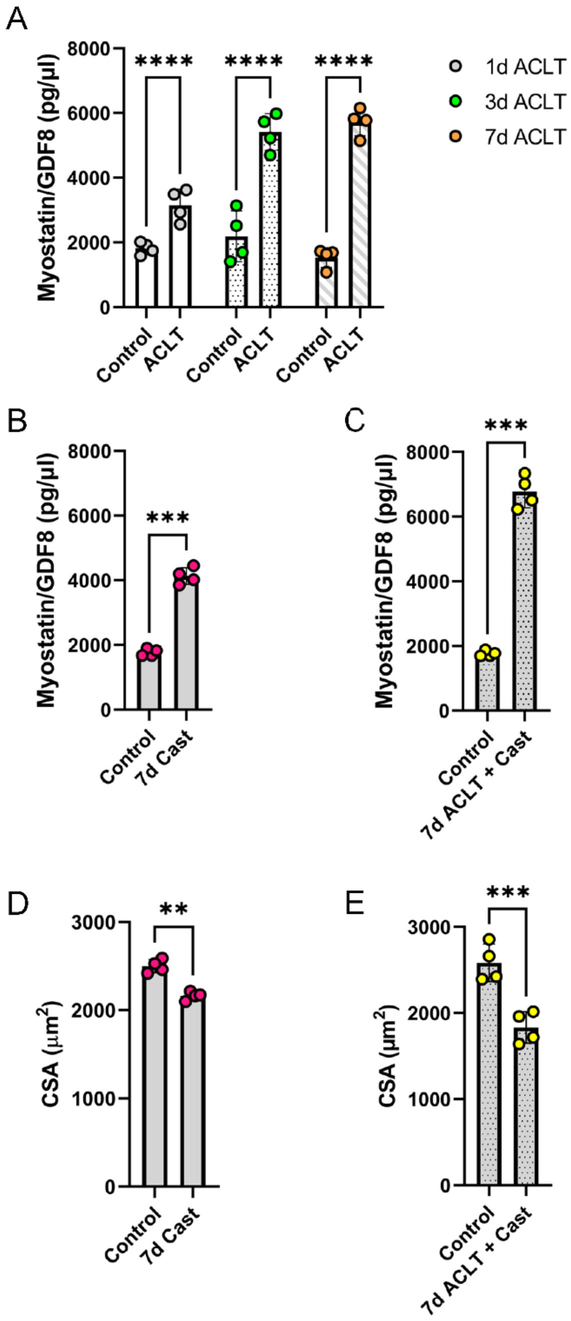

Supplemental Figure 5. Surgical ACL transection (ACLT) injury and unilateral hindlimb immobilization induce myostatin/GDF8 expression in mouse quadriceps muscle. A. Myostatin/GDF8 levels in the quadriceps after ACLT. Time period (1d, 3d, 7d) denotes time post-ACLT. B. Mice underwent unilateral hindlimb immobilization via casting for 7d, which induced an elevation in quadriceps myostatin/GDF8 levels as compared to the non-immobilized control limb. C. Mice underwent ACLT plus unilateral hindlimb immobilization via casting for 7d, which induced an elevation in quadriceps myostatin/GDF8 levels as compared to the non-immobilized control limb. D. Unilateral hindlimb immobilization via casting for 7d induced quadriceps atrophy as compared to the non-immobilized control limb. E. ACLT plus unilateral hindlimb immobilization via casting induced quadriceps atrophy as compared to the non-immobilized control limb. N=4 mice/group. **$p<0.01$, ***$p<0.005$, ****$p<0.001$ via paired sample t-test.

Supplemental Figure 6

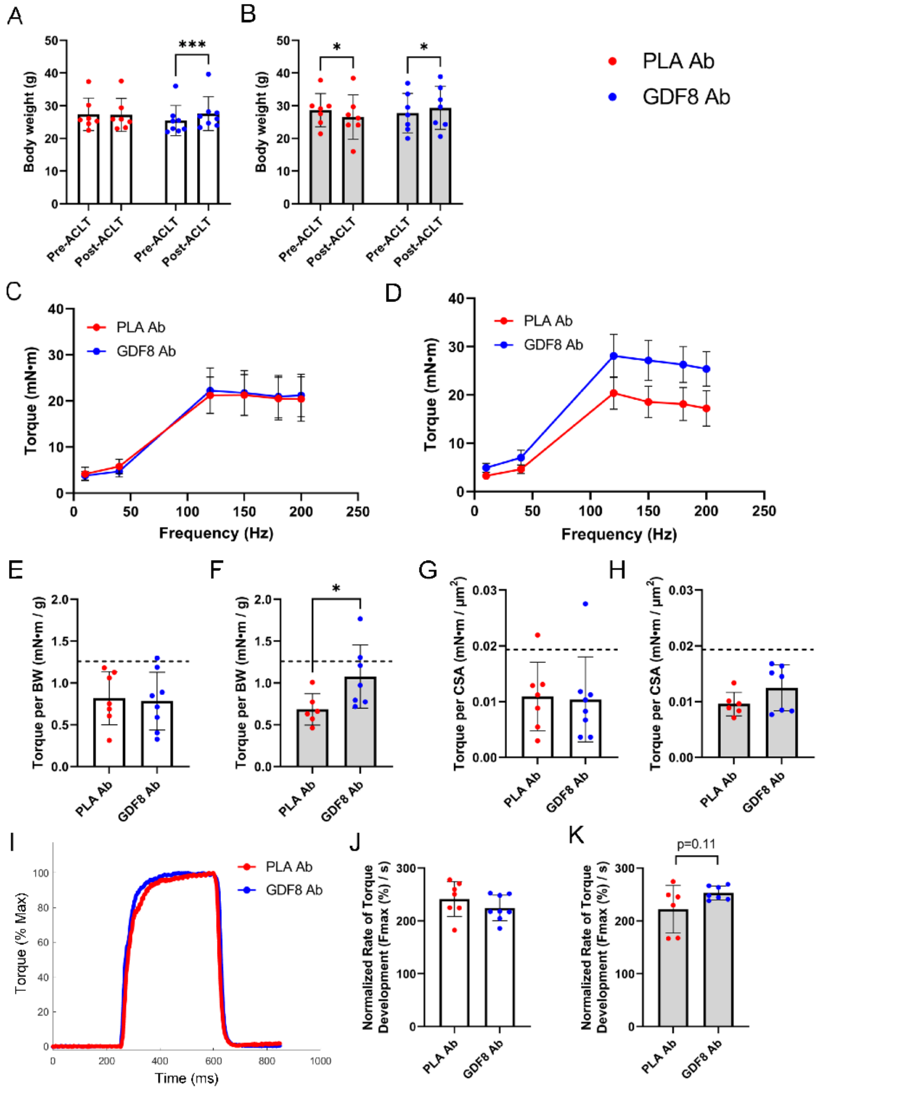

Supplemental Figure 6. Anti-myostatin/GDF8 antibody mitigates bodyweight loss and quadriceps weakness following ACL injury. A. Mouse bodyweight measured before ACL transection (pre-ACLT) and at one-week post-ACLT (drug x injury interaction p=0.009). B. Mouse bodyweight measured pre-ACLT and at two week post-ACLT (drug x injury interaction p=0.013). C. Force frequency curve of quadriceps torque at one-week post-ACLT. D. Force frequency curve of quadriceps torque at two weeks post-ACLT. E. Bodyweight-normalized peak quadriceps torque at one-week post-ACLT. F. Bodyweight-normalized peak quadriceps torque at two weeks post-ACLT. G. Mean fiber cross-sectional area (CSA) normalized peak quadriceps torque at one-week post-ACLT. H. Mean fiber cross-sectional area (CSA) normalized peak quadriceps torque at two weeks post-ACLT. I. Representative quadriceps tetanic graph from Fig 2D normalized to peak tetanic torque, so torque values represent a percentage of maximum torque. J. Knee extension rate of torque development adjusting for peak torque at one-week post-ACLT. K. Knee extension rate of torque development adjusting for peak torque at two weeks post-ACLT. N=7-8 mice/group. *p<0.05, ***p<0.005 represent Šidák's multiple comparison post hoc tests performed when significant interactions were detected via mixed models (A-B) or independent sample t-test (F). PLA Ab – placebo antibody, GDF8 Ab – GDF8 antibody, CSA – cross-sectional area.

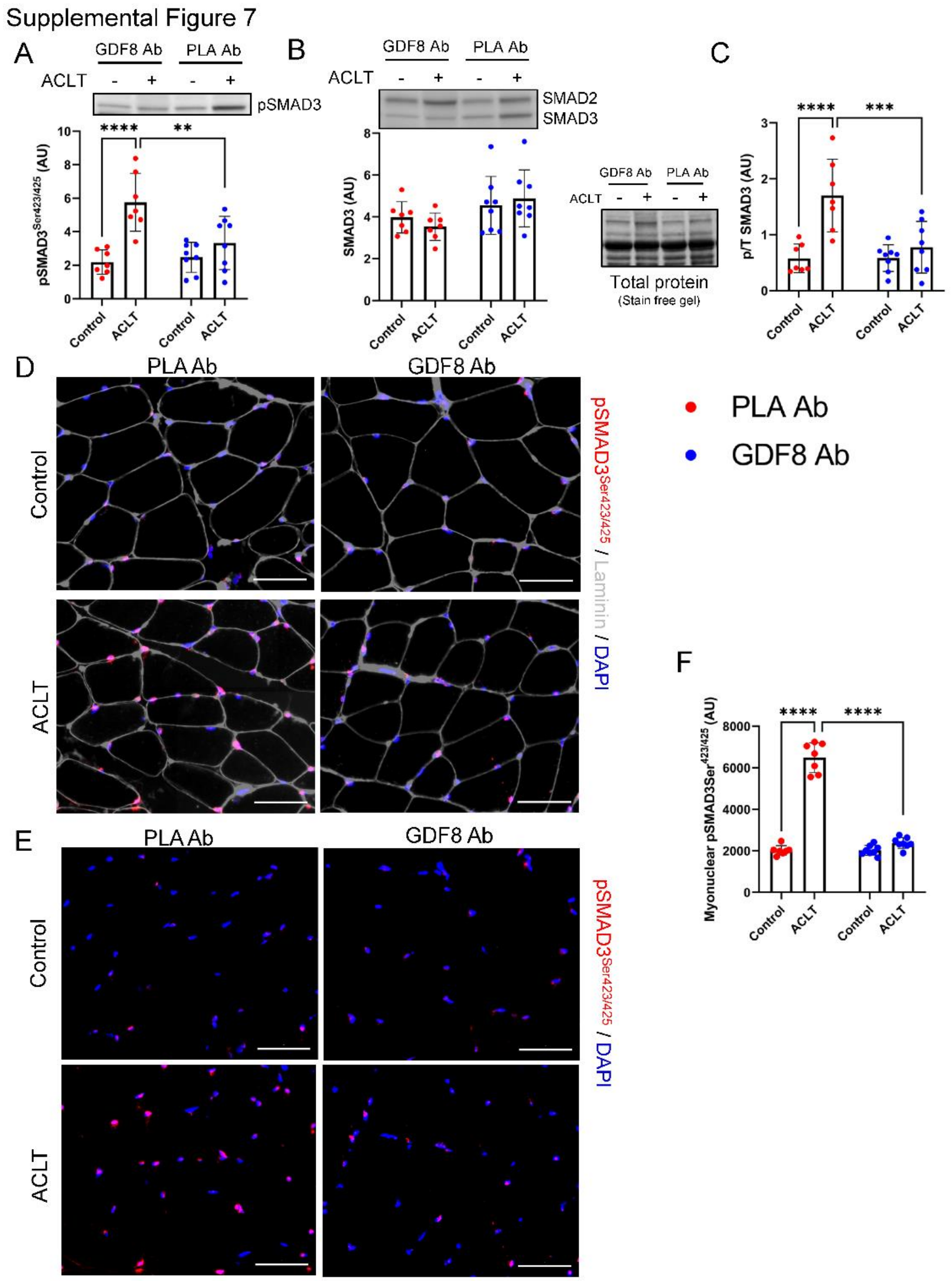

Supplemental Figure 7. Anti-myostatin/GDF8 antibody mitigates induction of SMAD3 activation in the quadriceps following ACL injury. A. phospho-SMAD3 expression in mouse quadriceps muscle 7d following ACL transection (ACLT) injury (drug x injury interaction p=0.002). B. SMAD3 protein expression in mouse quadriceps muscle 7d following ACL transection (ACLT) injury. C. pSMAD3 normalized to total SMAD3 in mouse quadriceps muscle 7d following ACL transection (ACLT) injury (drug x injury interaction p=0.003). D. Representative immunohistochemical images showing phospho-SMAD3+ nuclei (red), laminin (gray) and DAPI (blue) in mouse quadriceps muscle, scale bar=50µm. E. Representative immunohistochemical images showing phospho-SMAD3+ nuclei (red and DAPI (blue) in mouse quadriceps muscle, scale bar=50µm. F. Anti-GDF8 Ab mitigated the elevation in myonuclear phospho-SMAD3 after ACLT.N=7-8 mice/group (drug x injury interaction p<0.001). **p<0.01, ***p<0.005, ****p<0.001 represent Šidák's multiple comparison post hoc tests performed when significant interactions were detected via mixed models. PLA Ab – placebo antibody, GDF8 Ab – GDF8 antibody.

Supplemental Figure 8

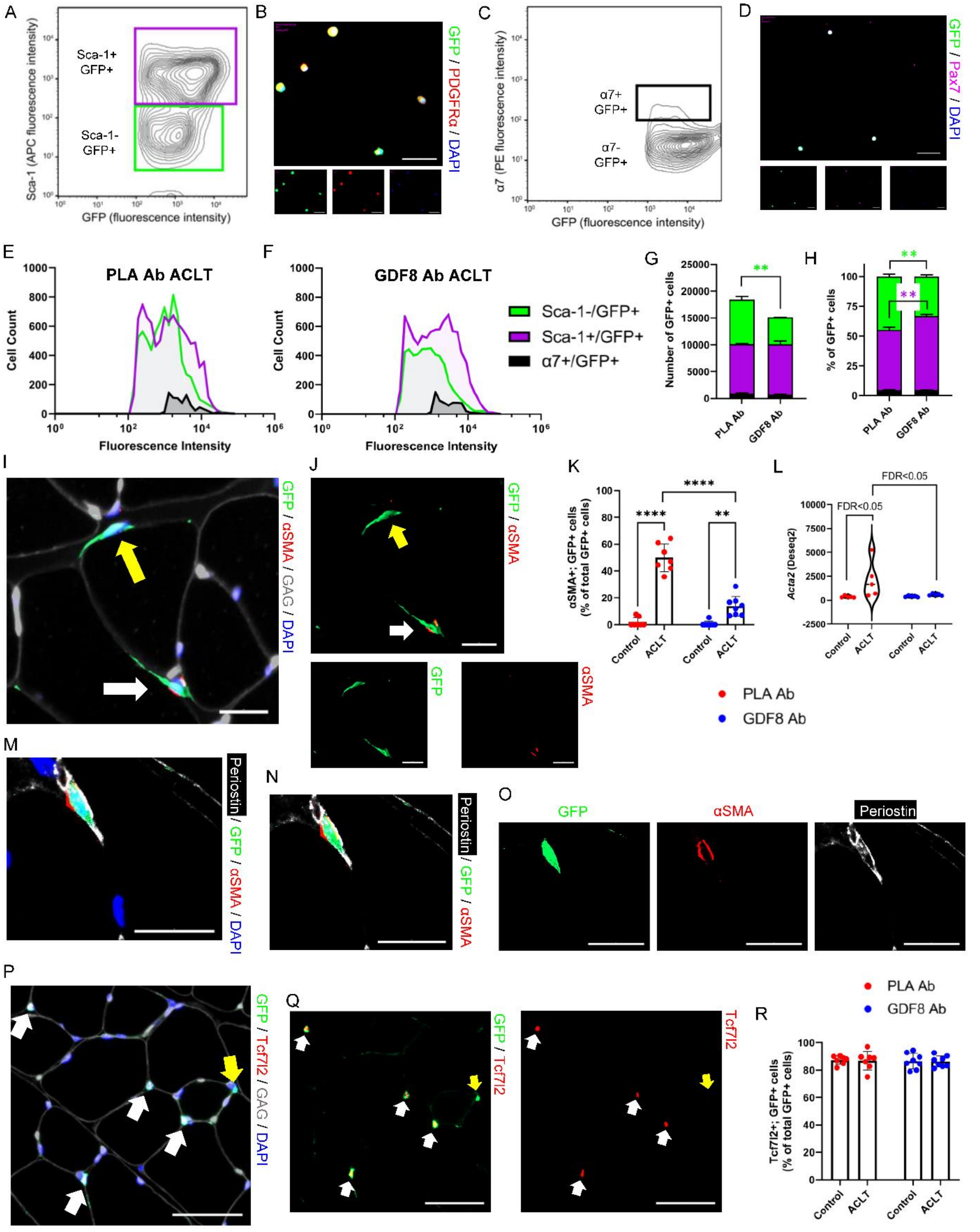

Supplemental Figure 8. Anti-GDF8 treatment alters fibrogenic cell identity and mitigates periostin expression following ACL injury. A. Representative FACS plot indicating Stem cell antigen-1 (Sca-1)+ and Sca-1-; Col1-GFP+ cells from quadriceps of mice after ACL transection (ACLT). B. Representative image of Sca-1+; Col1-GFP+ cells isolated from the quadriceps and co-stained for platelet derived growth factor receptor α (PDGFR α), scale bar=50μm. C. Representative FACS plot indicating Integrin-alpha 7 (α7)+ and α7-; Col1-GFP+ cells from quadriceps of mice after ACL transection (ACLT). D. Representative image of α7+; Col1-GFP+

cells isolated from the quadriceps and co-stained for Pax7, scale bar=50µm. E-F. Representative histograms demonstrating cell counts of: α7+; Col1-GFP+ cells, Sca-1+; Col1-GFP+ cells, and Sca-1-; Col1-GFP+ cells and GFP fluorescence intensity in the ACLT limbs of PLA Ab- and GDF8 Ab-treated mice. G. Raw Col1-GFP+ cell counts showing abundance of Sca-1-; Col1-GFP+ cells is altered in the ACLT-injured quadriceps of PLA Ab-treated mice compared to GDF8 Ab treatment (drug x cell-type interaction p=0.015). H. Relative abundance of Sca-1+; Col1-GFP+ cells and Sca-1-; Col1-GFP+ cells is altered in the ACLT-injured quadriceps of PLA Ab-treated mice compared to GDF8 Ab treatment (drug x cell-type interaction p=0.007). I. Representative IHC image denoting α smooth muscle actin (αSMA, red), Col1-GFP (green), glycosaminoglycans (GAG, gray), and DAPI (blue) in the ACLT-injured quadriceps. White arrow denotes a αSMA+; Col1-GFP+ cell, and yellow arrow denotes an αSMA-; Col1-GFP+ cell, scale bar=20µm. J. Representative images showing αSMA, Col1-GFP staining only and αSMA and Col1-GFP staining only, scale bar=20µm. K. GDF8 Ab treatment attenuates the elevated frequency of αSMA+; Col1-GFP+ cells in the quadriceps one-week after ACLT injury (drug x injury interaction p<0.001). L. Expression of *Acta2* (Actin Alpha 2, Smooth Muscle) is elevated following ACLT and is mitigated by treatment with GDF8 Ab. Gene expression levels were calculated using the DESeq2 method. FDR<0.05 denote specific comparisons. M. Representative IHC image denoting periostin (white), αSMA (red), Col1-GFP (green), and DAPI (blue) in the ACLT-injured quadriceps, scale bar=20µm. N. Representative IHC image showing periostin, αSMA, and Col1-GFP only, scale bar=20µm. O. Representative images showing periostin, αSMA, and Col1-GFP individually, scale bar=20µm. P. Representative IHC image denoting Tcf7l2 (red), Col1-GFP (green), glycosaminoglycans (GAG, gray), and DAPI (blue) in the ACLT-injured quadriceps. White arrows denote Tcf7l2+; Col1-GFP+ cells, and yellow arrow denotes a Tcf7l2-; Col1-GFP+ cell, scale bar=50µm. Q. Representative images showing Tcf7l2 and Col1-GFP staining only and Tcf7l2 staining only, scale bar=50µm. R. Tcf7l2+, Col1-GFP+ cell frequency is unchanged one-week after ACLT. N=2 mice/group (A-H), 7-8 mice/group (I-K, M-R), 5 mice/group (L). **p<0.01 and ****p<0.001 represent Šidák's multiple comparison post hoc tests performed when significant interactions were detected via mixed models. PLA Ab – placebo antibody, GDF8 Ab – GDF8 antibody, GFP – green fluorescent protein, PDGFRα - platelet derived growth factor receptor α, Sca-1 – Stem cell antigen-1, α7 – Integrin α7.

Supplemental Figure 9

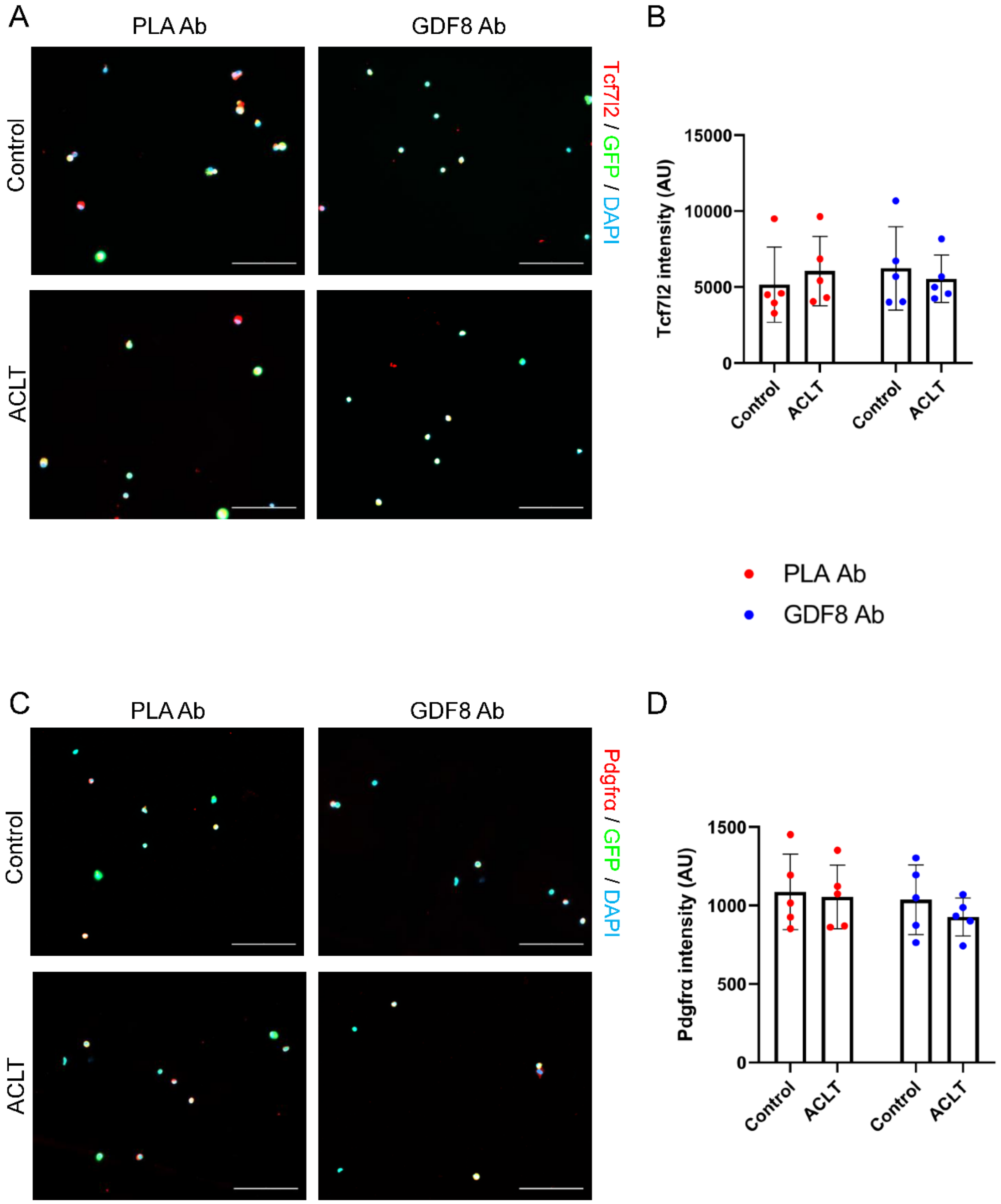

<u>Supplemental Figure 9</u>. Expression of Tcf7l2 and Platelet derived growth factor receptor (Pdgfrα) are unchanged in collagen 1-GFP+ cells after ACLT. A. Representative image of collagen 1-GFP+ cells isolated from the quadriceps and co-stained for Tcf7l2, scale bar=100µm. B. Quantification Tcf7l2 intensity. C. Representative image of collagen 1-GFP+ cells isolated from the quadriceps and co-stained for Pdgfrα, scale bar=100µm. D. Quantification Pdgfrα intensity. N=5 mice/group. PLA Ab – placebo antibody, GDF8 Ab – GDF8 antibody.

Supplemental Figure 10

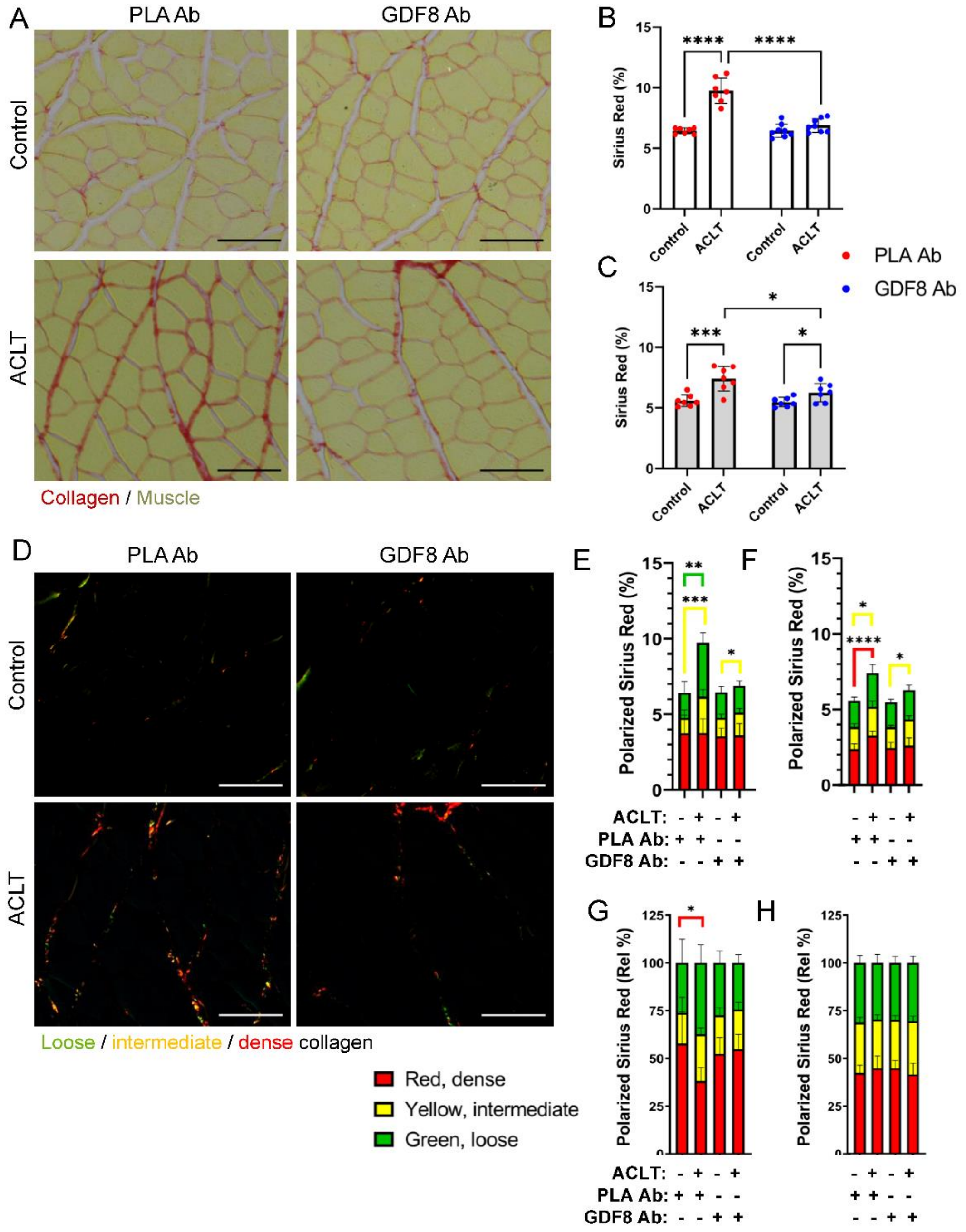

<u>Supplemental Figure 10</u>. Treatment with anti-GDF8 antibody reduces quadriceps fibrosis following ACL injury. A. Representative histochemical images of Sirius red, scale bar=100µm. B. Sirius red collagen content is elevated in quadriceps muscle one-week following ACLT and is rescued by treatment with GDF8 Ab (drug x injury interaction $p<0.001$). C. Sirius red collagen content is elevated in quadriceps muscle two weeks following ACLT and is rescued by treatment with GDF8 Ab (drug x injury interaction $p=0.035$). D. Representative images of Sirius red staining under polarized light, scale bar=100µm. E. Collagen packing as a fraction of total muscle area is altered one-week after ACLT. F. Collagen packing as a fraction of total muscle area is altered two weeks after ACLT. G. Collagen packing as a fraction of collagen staining is altered one week after ACLT with placebo treatment. H. Collagen packing as a fraction of collagen staining two weeks after ACLT. N=7-8 mice/group. *$p<0.05$, **$p<0.01$, ***$p<0.005$, ****$p<0.001$ represent Šidák's multiple comparison post hoc tests performed when significant interactions were detected via mixed models. PLA Ab – placebo antibody, GDF8 Ab – GDF8 antibody.

Supplemental Figure 11

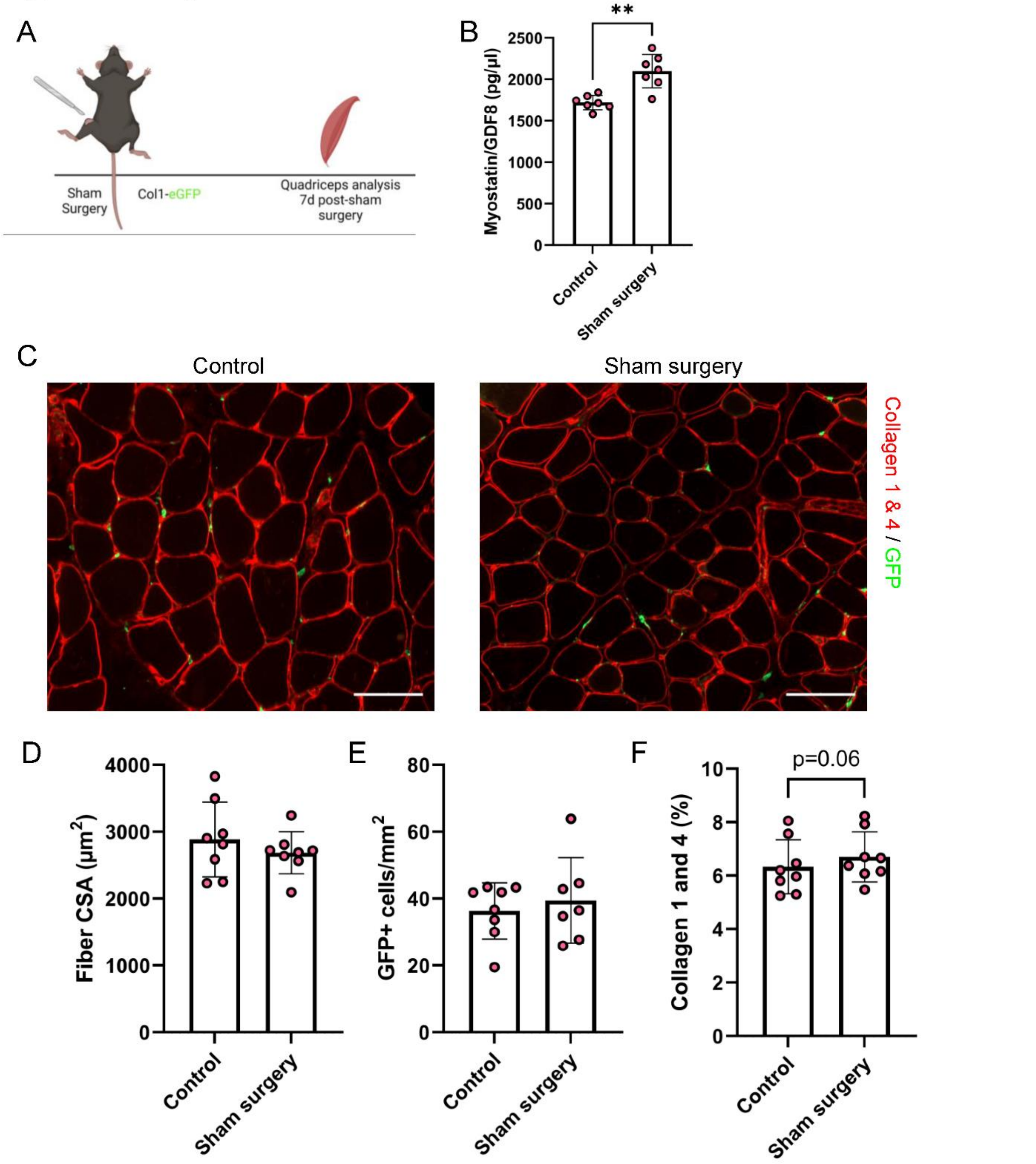

Supplemental Figure 11. Sham surgery does not alter quadriceps fiber size or fibrosis. A. Study diagram; mice underwent sham surgery and quadriceps muscles were collected 7d afterward. B. Sham surgery induces a significant elevation in quadriceps GDF8. C. Representative IHC images of collagens 1 and 4 and collagen 1-GFP+ cells in quadriceps muscle, scale bar=100µm. D. Sham surgery does not alter quadriceps muscle fiber size. E. Sham surgery does not alter quadriceps muscle collagen 1-GFP+ cell abundance. F. Sham surgery showed a trend for elevated quadriceps muscle collagen content. **$p<0.01$ via paired t-test. N=7 mice (B), N=8 mice (C-F).

Supplemental Figure 12

A

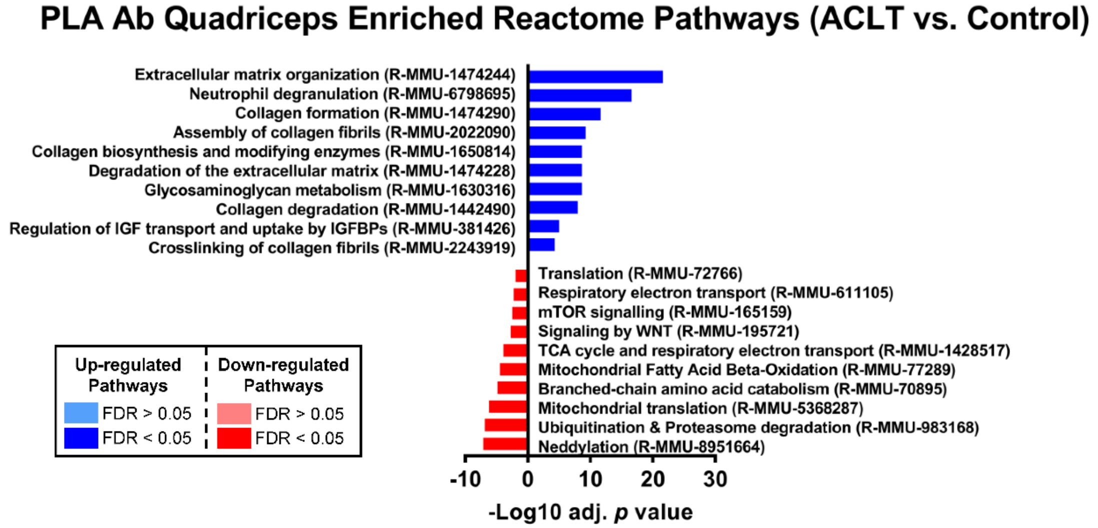

B

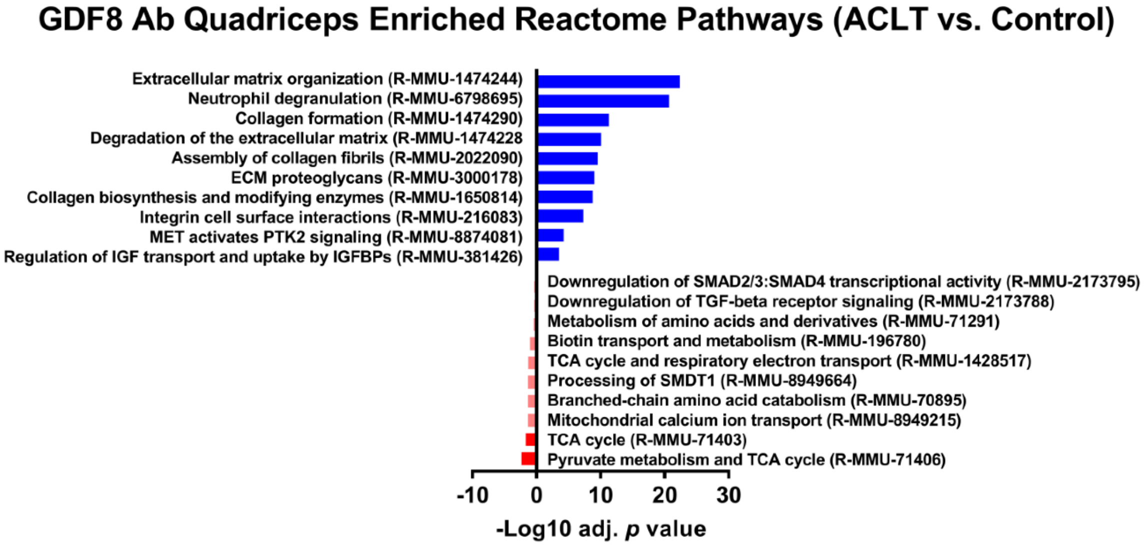

Supplemental Figure 12. ACLT induces substantial alterations to the quadriceps transcriptome. A. Most activated (blue) and inhibited (red) pathways associated with ACLT as compared to Control limb in GDF8-Ab treated mice using REACTOME Pathway Knowledgebase. B. Most activated (blue) and inhibited (red) pathways associated with ACLT as compared to Control limb in PLA-Ab treated mice using REACTOME Pathway Knowledgebase. Pathways having false discovery rate (FDR) P values < 0.05 for either direction are bright colors; pathways having FDR P values > 0.05 for either direction are muted colors. PLA Ab – placebo antibody, GDF8 Ab – GDF8 antibody.

Supplemental Figure 13

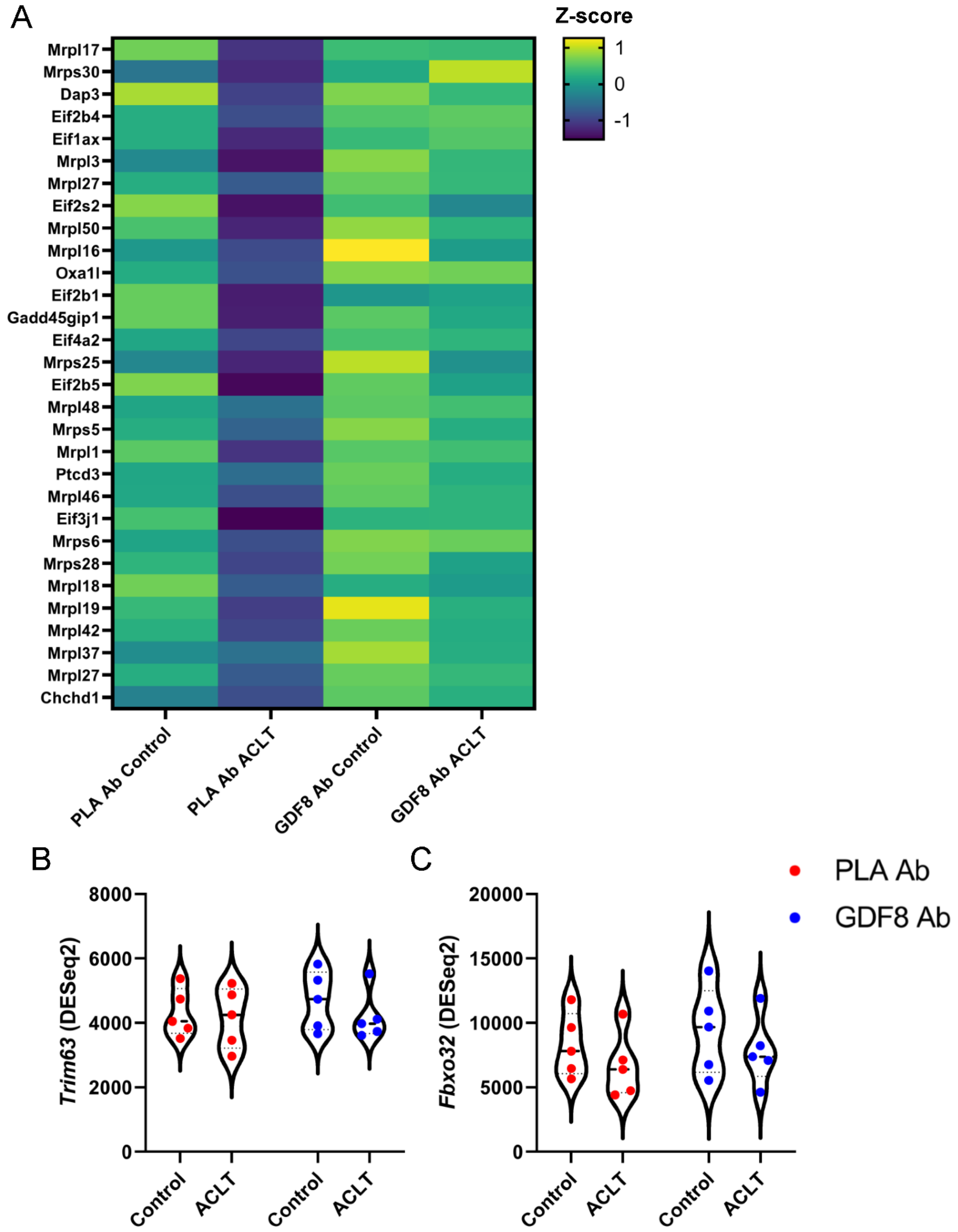

Supplemental Figure 13. Anti-GDF8 treatment after ACLT enhances expression of genes associated muscle protein anabolism. A. Heat map depicting relative abundance of genes associated with translation. B. Expression of *Trim63* is unchanged in the quadriceps muscle following ACLT. C. Expression of *Fbxo32* is unchanged in the quadriceps muscle following ACLT. N=5 mice/group. PLA Ab – placebo antibody, GDF8 Ab – GDF8 antibody.

Supplemental Figure 14

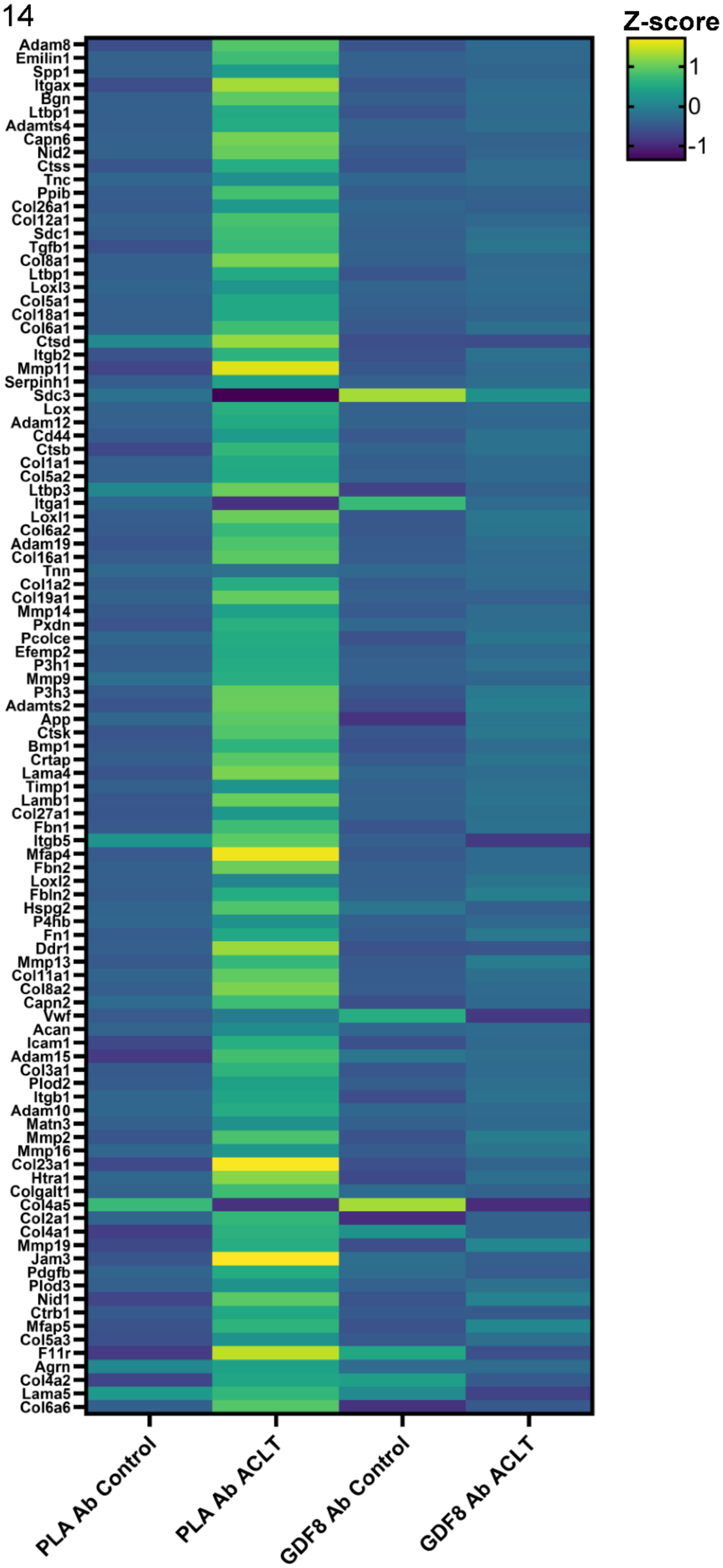

Supplemental Figure 14. Anti-GDF8 treatment after ACLT mitigates elevated expression of genes associated with quadriceps extracellular matrix remodeling, collagen biosynthesis, and cross-linking. Heat map depicting relative abundance of genes associated with extracellular matrix remodeling, collagen biosynthesis, and cross-linking. PLA Ab – placebo antibody, GDF8 Ab – GDF8 antibody.

Supplemental Figure 15

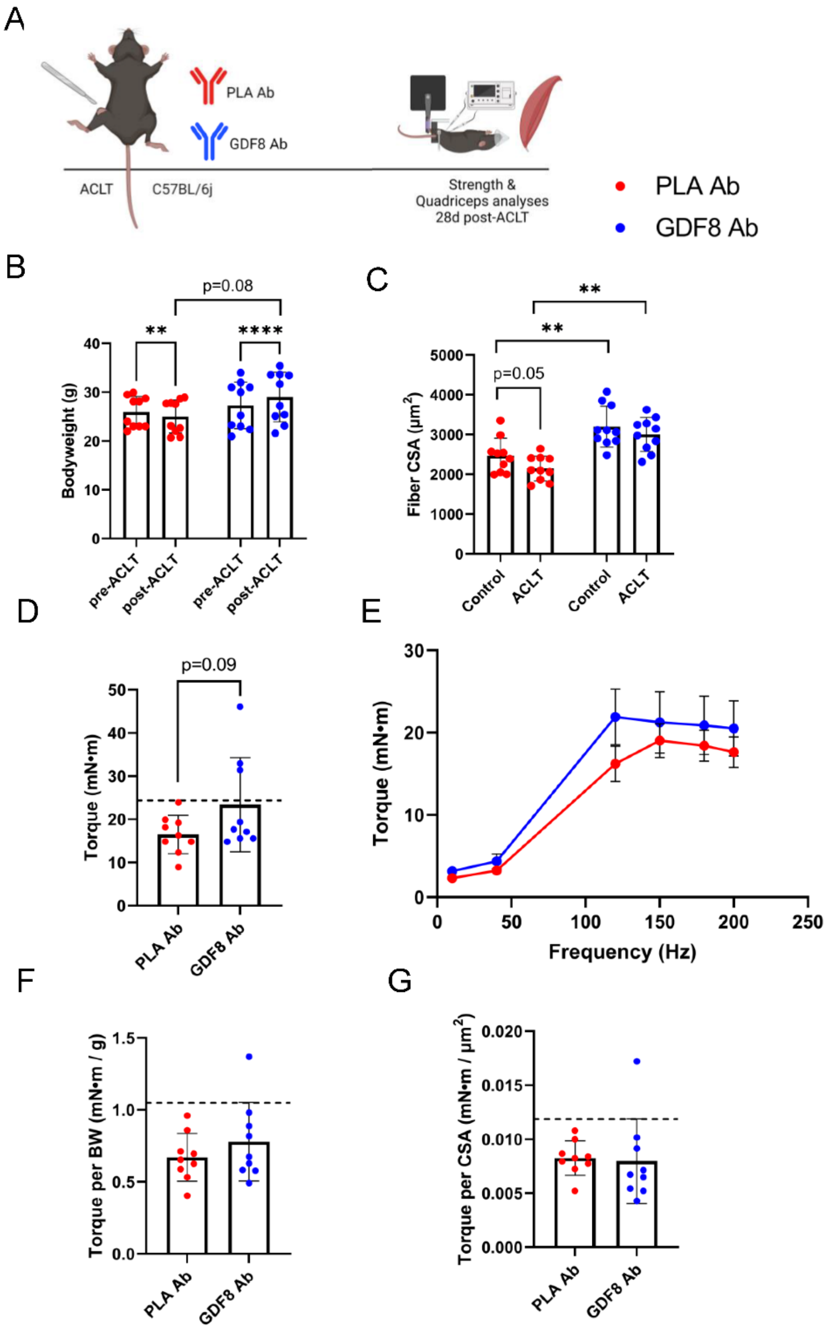

Supplemental Figure 15. Longer term anti-GDF8 antibody treatment mitigates quadriceps atrophy and weakness and bodyweight loss following ACLT. A. Study diagram; mice underwent ACLT and were treated biweekly with subcutaneous injections and quadriceps muscles were collected 28d afterward. B. Anti-GDF8 treatment protected against bodyweight loss after ACLT (drug x injury interaction $p<0.001$). C. ACLT results in quadriceps muscle fiber atrophy in mice that remains through 28 days after ACL injury and is rescued by treatment with GDF8 Ab (drug x injury interaction $p=0.042$). D. Anti-GDF8 treatment showed a trend to enhance knee extension peak isometric torque 28 days after ACLT (dashed line represents mean value from control mice). E. Force frequency curve for quadriceps torque at 7d post-ACLT. F. Bodyweight normalized peak quadriceps torque. G. Mean fiber cross-sectional area (CSA) normalized peak quadriceps torque. N=10 mice/group, **$p<0.01$, ****$p<0.001$ represent Šidák's multiple comparison post hoc tests performed when significant interactions were detected via mixed models. PLA Ab – placebo antibody, GDF8 Ab – GDF8 antibody, CSA – cross-sectional area.

Supplemental Figure 16

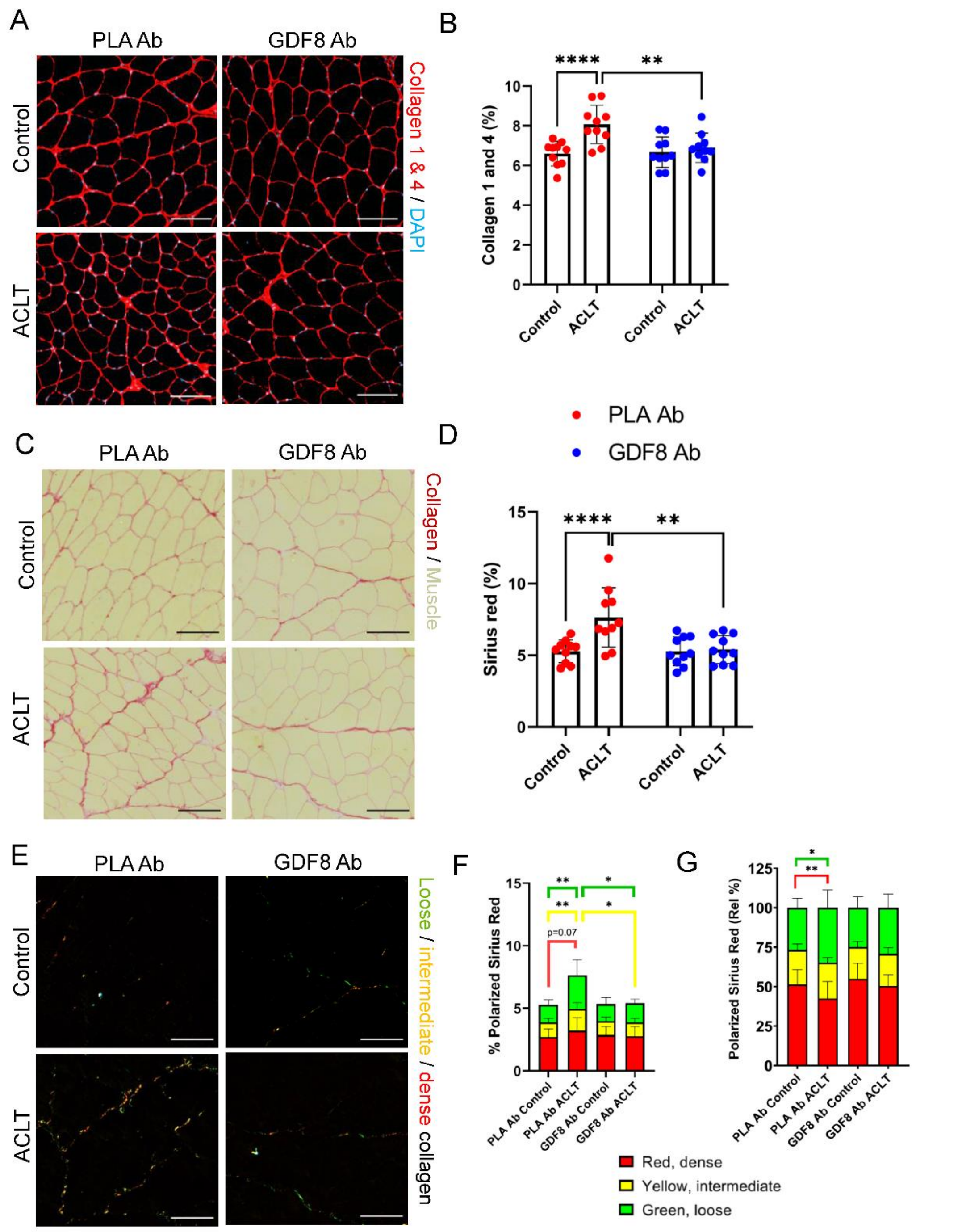

Supplemental Figure 16. Longer term anti-GDF8 antibody treatment mitigates quadriceps fibrosis following ACLT. A. Representative IHC images of collagens 1 and 4 in quadriceps muscle, scale bar=100µm. B. Content of collagens 1 and 4 is elevated in quadriceps 28 days after ACL injury and is rescued by GDF8 Ab (drug x injury interaction p=0.004). C. Representative histochemical images of Sirius red, scale bar=100µm. D. Sirius red collagen content is elevated in quadriceps muscle following ACLT and is rescued by treatment with GDF8 Ab (drug x injury interaction p=0.001). E. Representative images of Sirius red staining under polarized light, scale bar=100µm. F. Collagen packing as a fraction of total muscle area is altered by ACLT. G. Collagen packing as a fraction of collagen staining is altered by ACLT with placebo treatment. N=10 mice/group, *p<0.05, **p<0.01, ****p<0.001 represent Šidák's multiple comparison post hoc tests performed when significant interactions were detected via mixed models. PLA Ab – placebo antibody, GDF8 Ab – GDF8 antibody.

Supplemental Figure 17

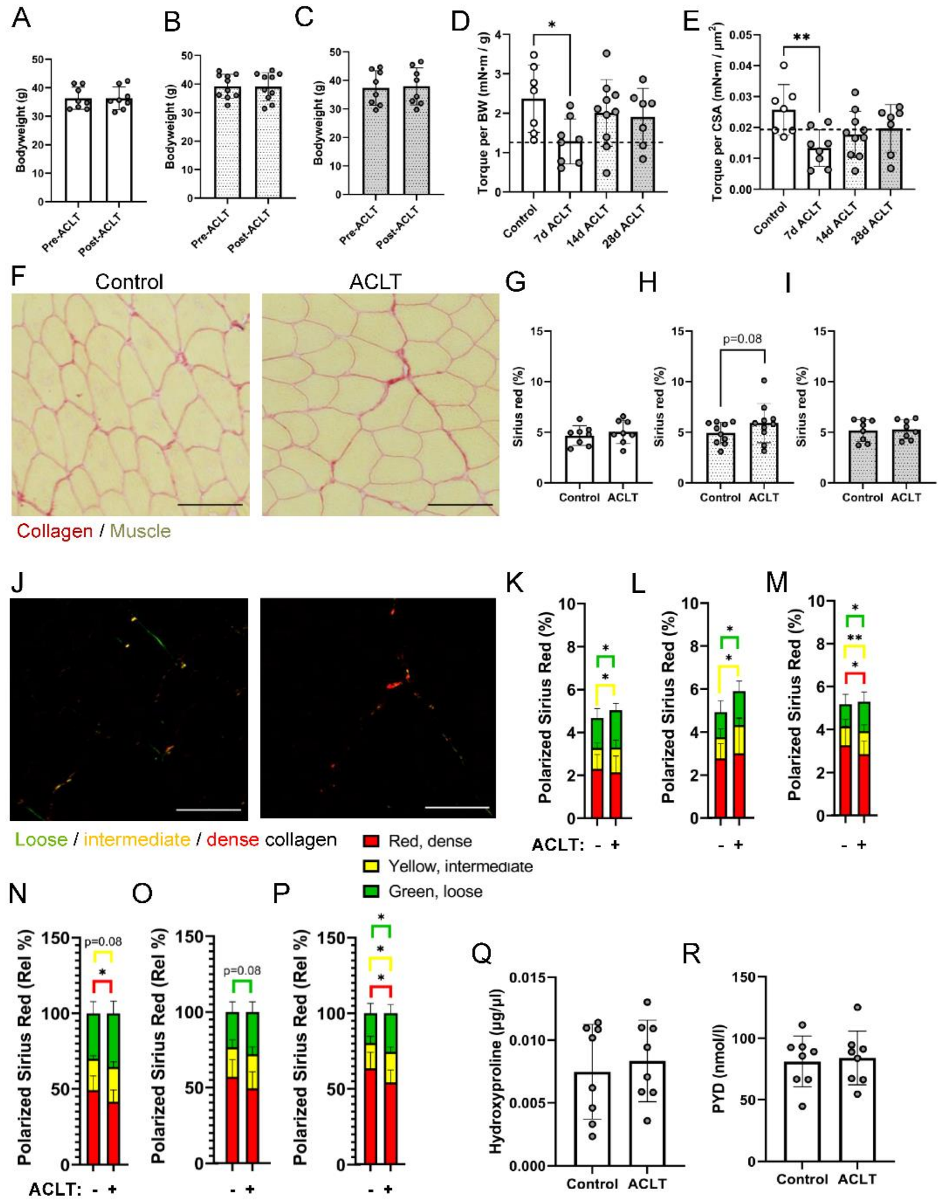

Supplemental Figure 17. GDF8 knockout mitigates quadriceps weakness and fibrosis following ACL injury. A-C. GDF8 knock out (GDF8KO) preserves bodyweight A. one-week, B. two weeks, and C. four weeks after ACLT. D. Bodyweight normalized peak quadriceps torque (dashed line represents mean value from wild type mice). E. Mean fiber cross-sectional area (CSA) normalized peak quadriceps torque (dashed line represents mean value from wild type mice). F. Representative histochemical images of Sirius red, scale bar=100µm. G. GDF8KO protects against increased quadriceps collagen content one-week post-ACLT. H. Quadriceps collagen is elevated at 14d post-ACLT following ACLT. I. GDF8KO protects against increased quadriceps collagen content four weeks post-ACLT. J. Representative images of Sirius red staining under polarized light, scale bar=100µm. K-M. Collagen packing as a fraction of total muscle area is altered at K. one-week, L. two weeks, and M. four weeks after ACLT in GDF8KO mice. N-P. Collagen packing as a fraction of collagen staining is altered N. one-week, O. two weeks, and P. four weeks after ACLT in GDF8KO mice. Q. GDF8KO mice are protected against increases in quadriceps hydroxyproline abundance one-week following ACLT. R. GDF8KO mice are protected against increases in quadriceps pyridinoline (PYD) cross-linking one-week following ACLT. N=8-10 mice/group. *p<0.05, **p<0.01 via one factor ANOVA (D and E). *p<0.05, **p<0.01 via paired sample t-test (K-P).

Supplemental Figure 18

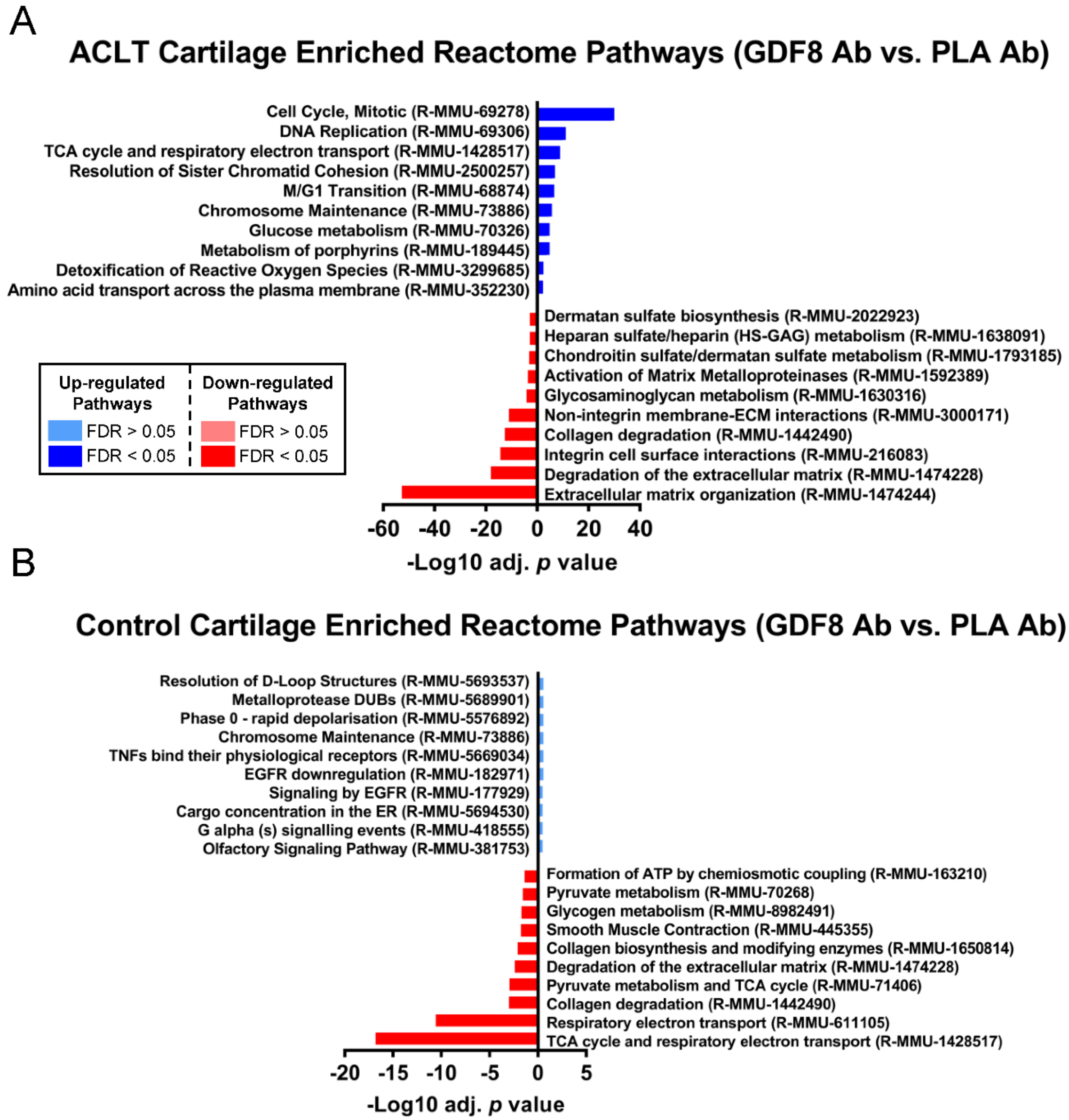

Supplemental Figure 18. Anti-GDF8 treatment mitigates articular cartilage extracellular matrix remodeling transcriptomic signature after ACLT. A. Most activated (blue) and inhibited (red) pathways associated with GDF8 inhibition in the ACLT-injured articular cartilage using REACTOME Pathway Knowledgebase. B. Most activated (blue) and inhibited (red) pathways associated with GDF8 inhibition in Control articular cartilage using REACTOME Pathway Knowledgebase. Pathways having false discovery rate (FDR) P values < 0.05 for either direction are bright colors; pathways having FDR P values > 0.05 for either direction are muted colors. RNA was pooled from 4 mice to yield 2 biological replicates per group (n = 2 replicates per injury and treatment).

Supplemental Figure 19

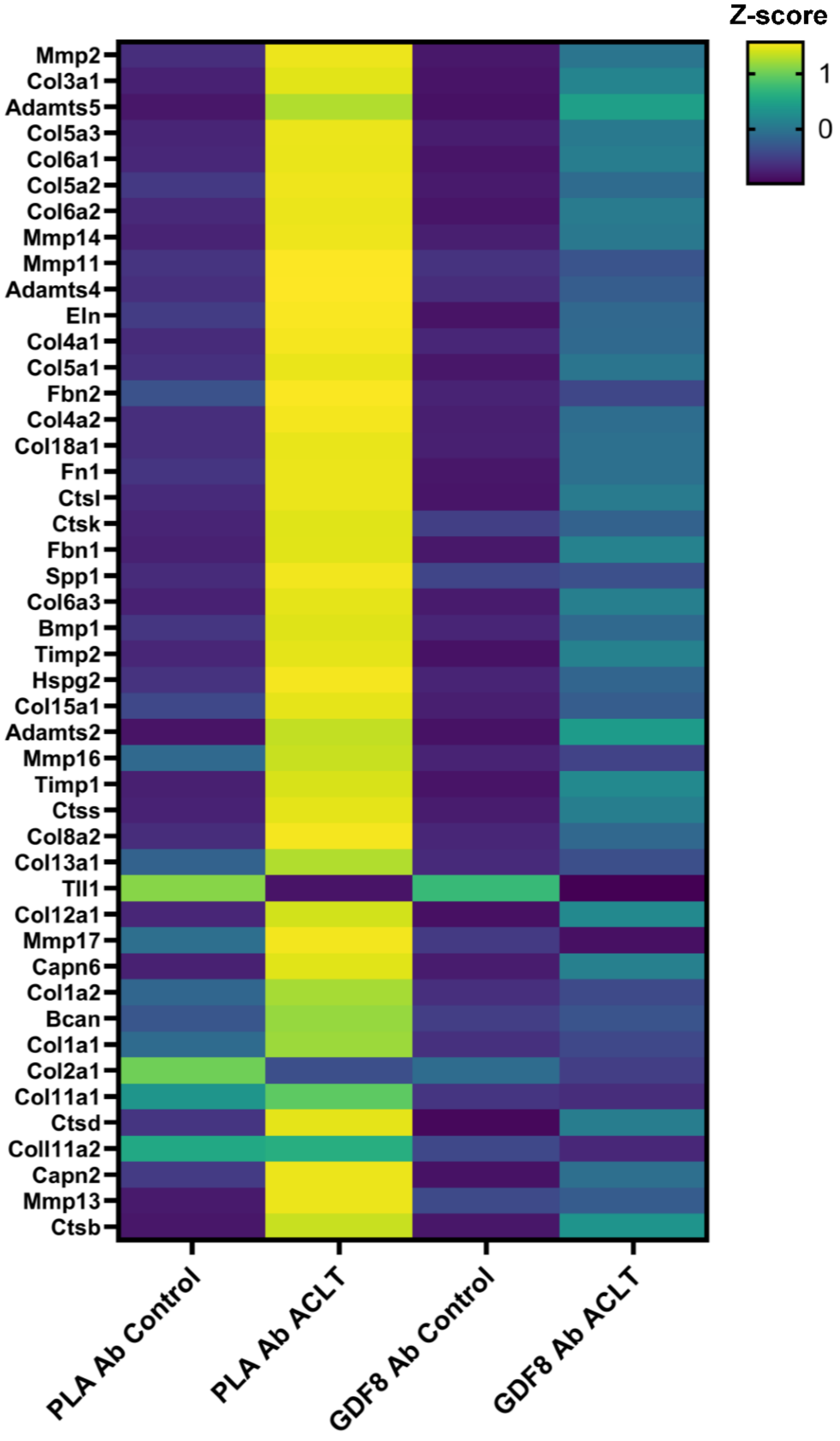

Supplemental Figure 19. Anti-GDF8 treatment after ACLT mitigates elevated expression of extracellular matrix remodeling genes in articular cartilage. Heat map depicting relative abundance of genes associated with extracellular matrix remodeling, collagen biosynthesis and degradation. PLA Ab – placebo antibody, GDF8 Ab – GDF8 antibody.

Supplemental Figure 20

A

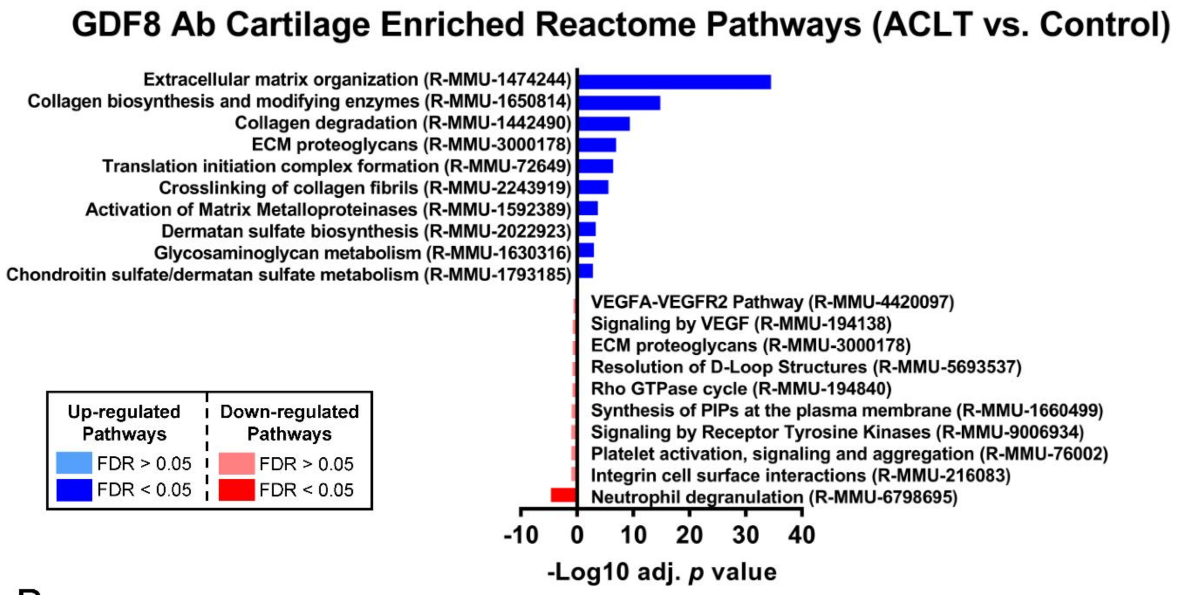

B

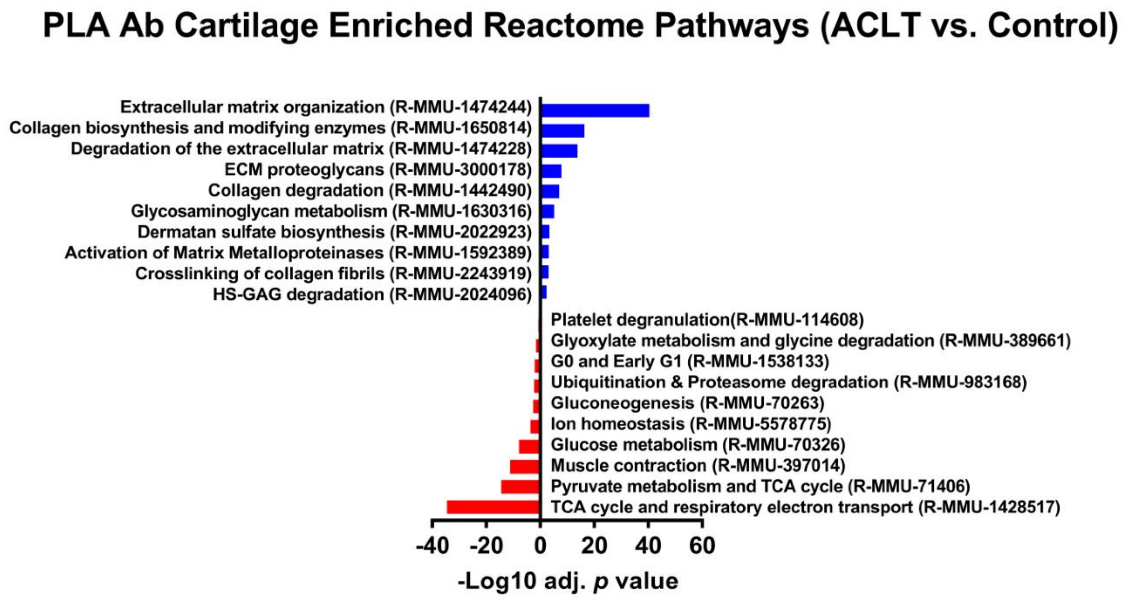

Supplemental Figure 20. ACLT induces substantial alterations to the articular cartilage transcriptome. A. Most activated (blue) and inhibited (red) pathways associated with ACLT as compared to Control limb in GDF8-Ab treated mice using REACTOME Pathway Knowledgebase. B. Most activated (blue) and inhibited (red) pathways associated with ACLT as compared to Control limb in PLA-Ab treated mice using REACTOME Pathway Knowledgebase. Pathways having false discovery rate (FDR) P values < 0.05 for either direction are bright colors; pathways having FDR P values > 0.05 for either direction are muted colors. PLA Ab – placebo antibody, GDF8 Ab – GDF8 antibody.

Supplemental Figure 21

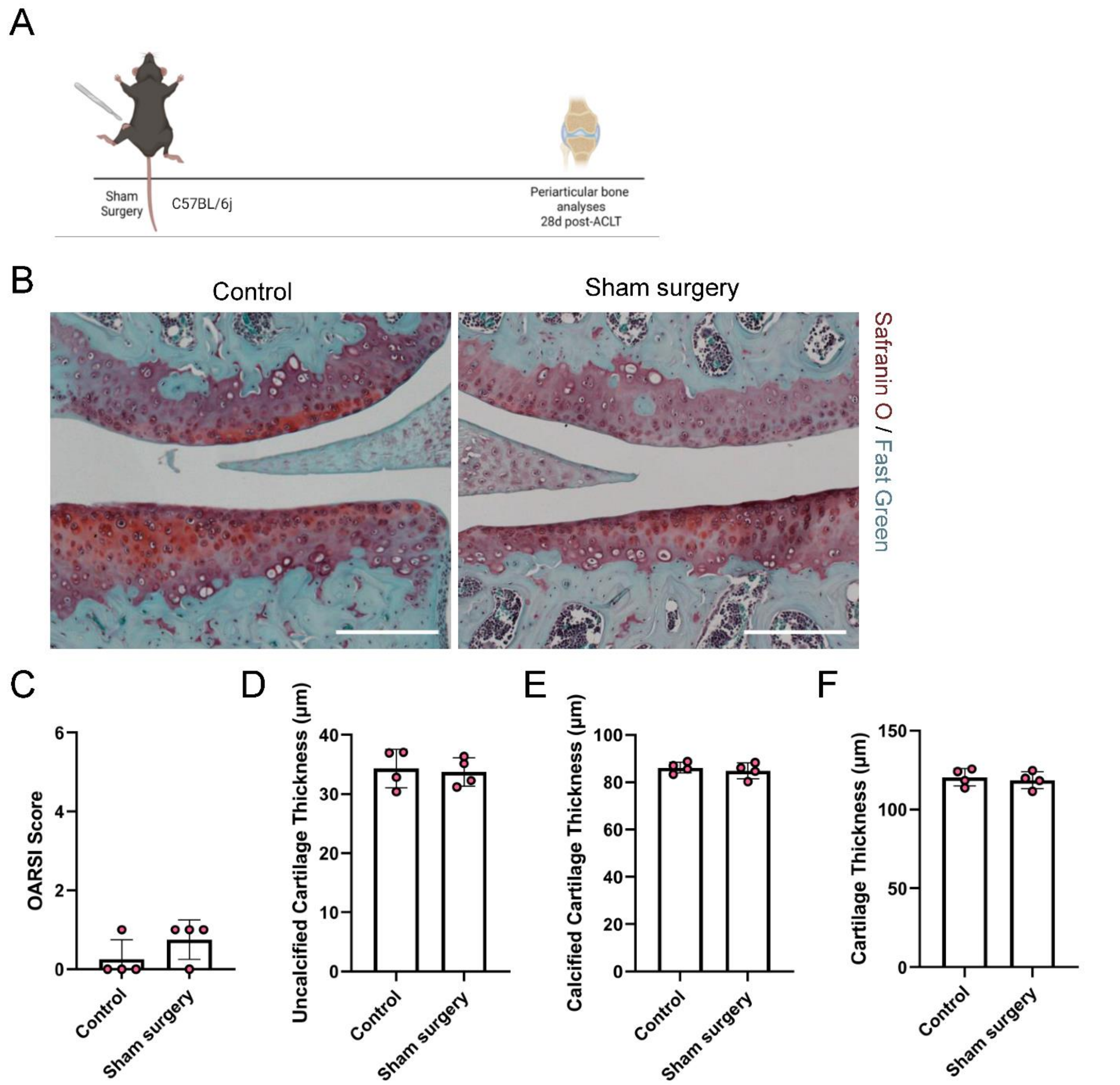

Supplemental Figure 21. Sham surgery does not significantly damage articular cartilage. A. Study diagram; mice underwent sham surgery and whole knee joints were collected 28 days afterward. B. Representative images of safranin O and fast green from whole fixed knee joints, scale bar=200µm. C. Medial tibial plateau joint score based on the OARSI scoring system shows slight elevation in cartilage damage 28 days following sham surgery. D. Quantification of uncalcified cartilage zones 28 days after sham surgery. E. Quantification of calcified cartilage zones 28 days after sham surgery. F. Total articular cartilage thickness 28 days after sham surgery. N=4 mice.

Supplemental Figure 22

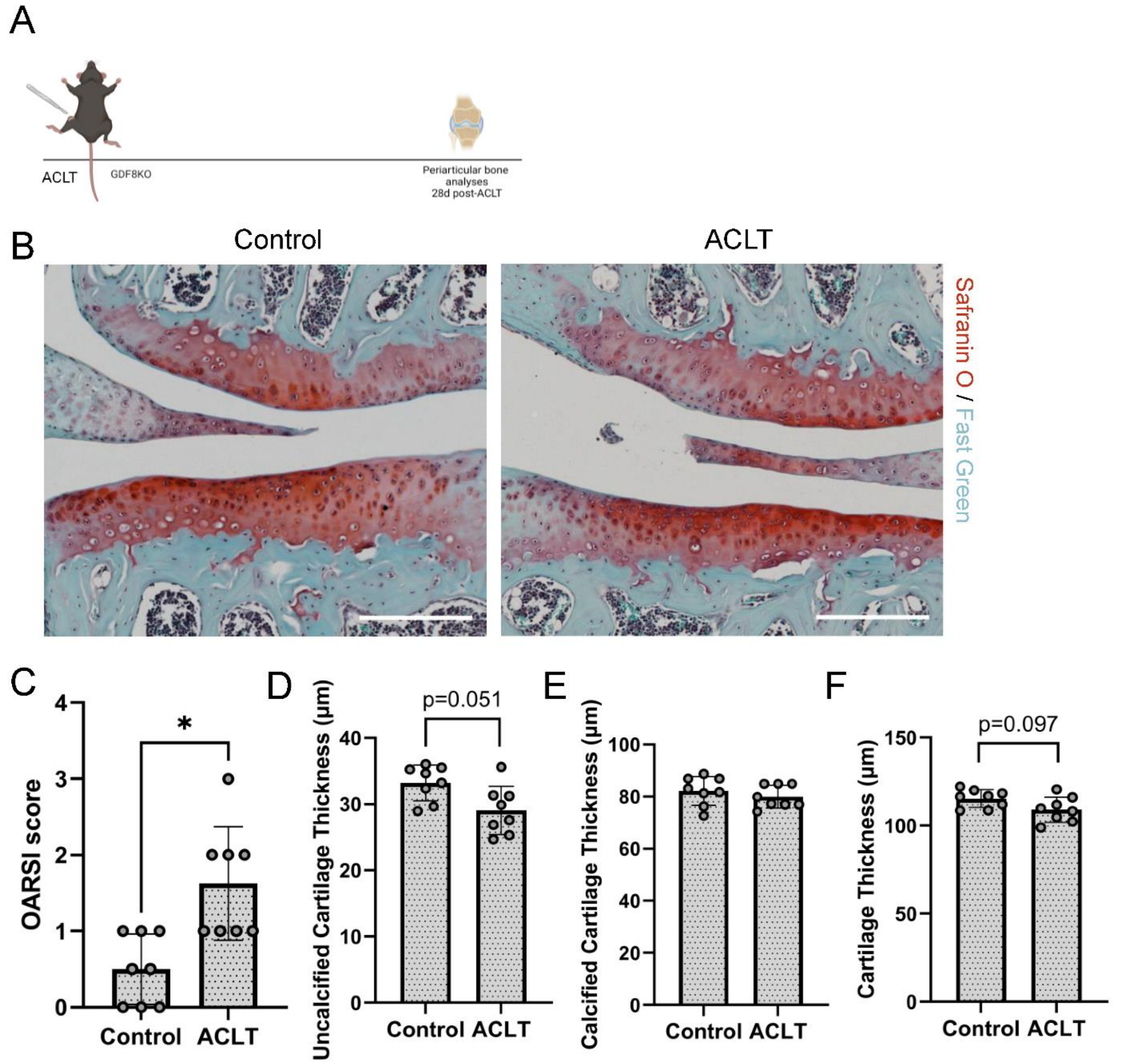

Supplemental Figure 22. GDF8 knockout attenuates the severity of post-traumatic knee osteoarthritis. A. Study diagram; mice underwent ACLT and whole knees were collected 28 days post-ACLT for histopathology. B. Representative images of safranin O and fast green from whole fixed knee joints, scale bar=200µm. C. Medial tibial plateau joint score based on the OARSI scoring system shows mild severity post-traumatic osteoarthritis (PTOA) severity in GDF8 knockout (GDF8KO) animals. D. Quantification of uncalcified cartilage zones shows thinning 28 days after ACLT in GDF8KO mice. E. Quantification of calcified cartilage zones shows protection after ACLT in GDF8KO mice. H. Total articular cartilage tended to be thinner 28 days after ACLT in GDF8KO mice. N=8 mice. *p<0.05 via paired sample t-test.

Supplemental Figure 23

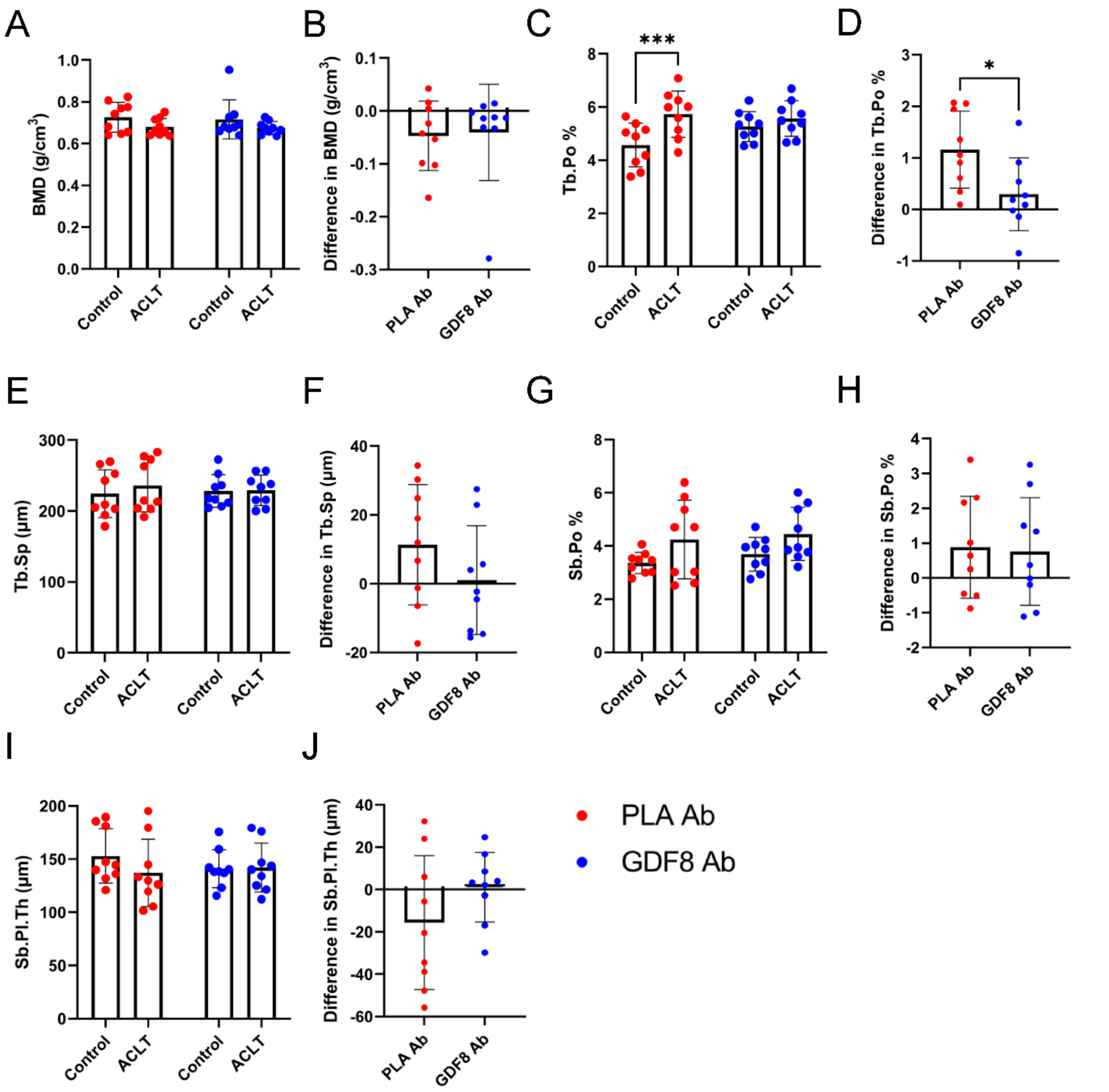

Supplemental Figure 23. GDF8 antibody mitigates trabecular bone porosity following ACL injury. There was no effect of drug treatment or ACLT on trabecular bone mineral density (BMD, A-B). Anti-GDF8 treatment mitigated the increase in trabecular porosity 28 days following ACLT (Tb.Po, C-D, C. drug x injury interaction p=0.023). There was no effect of drug treatment or ACLT on trabecular separation (Tb.Sp, E-F) or on subchondral bone plate porosity (Sp.Po, G-H) or subchondral bone plate thickness (Sb.Pl.Th, I-J). N=9 mice/group. ***p<0.005 represent Šidák's multiple comparison post hoc tests performed when significant interactions were detected via mixed models (C), *p<0.05 via paired sample t-test (F).

Supplemental Figure 24

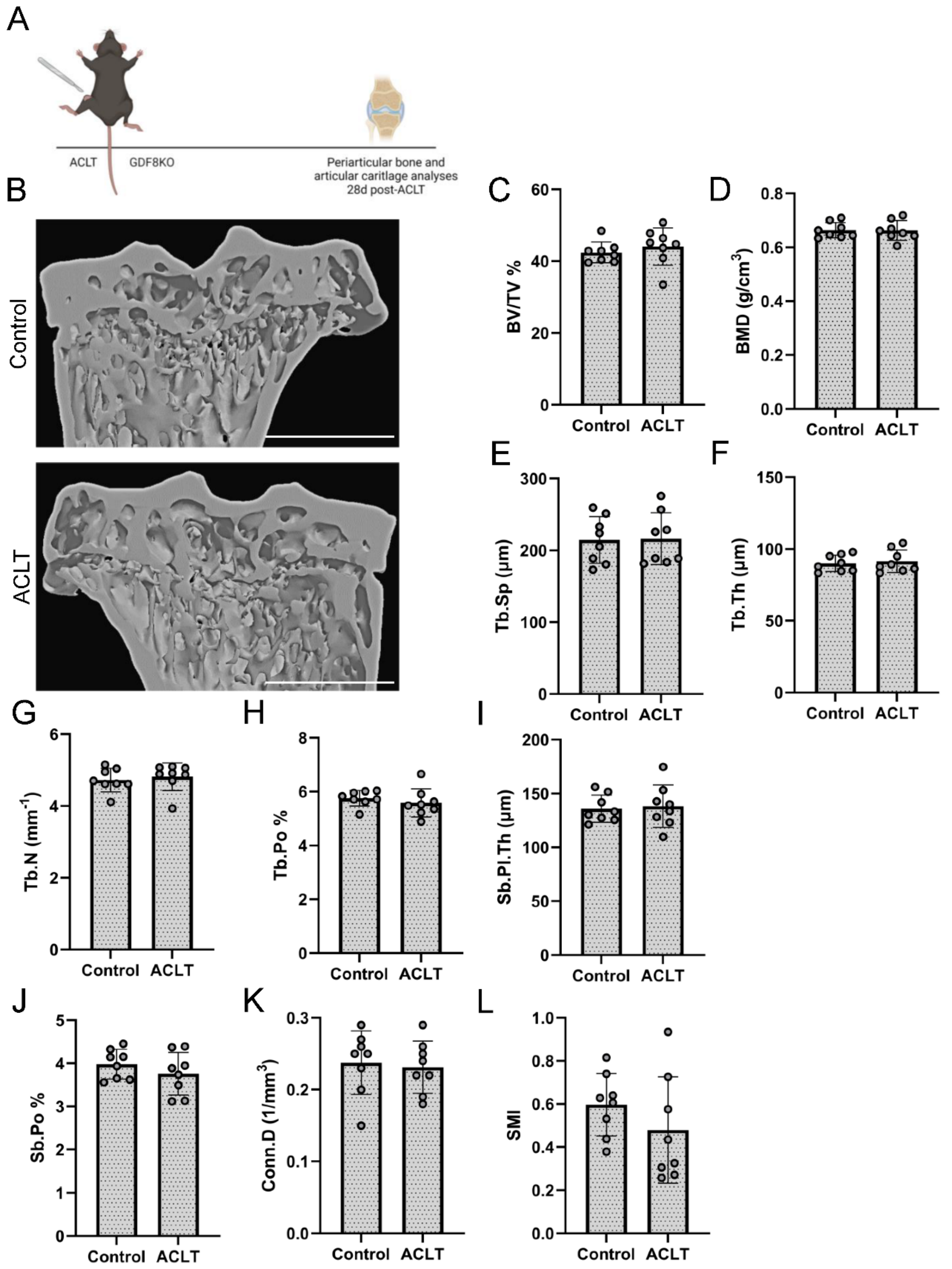

Supplemental Figure 24. GDF8 knockout preserves periarticular bone microarchitecture following ACL injury. A. Study diagram; GDF8 knockout (GDF8KO) mice had whole knees fixed for microCT analysis 28 days post ACL transection (ACLT). B. Representative microCT images showing 3D reconstruction of coronal sections, scale bar=1mm. GDF8KO preserved bone volume fraction (BV/TV %, C), bone mineral density (BMD, D), trabecular separation (Tb.Sp, E), trabecular thickness (Tb.Th, F) and trabecular number (Tb.N, G), trabecular porosity (Tb.Po, H), subchondral bone plate thickness (Sb.Pl.Th, I), subchondral bone plate porosity (Sp.Po, J), connectivity density (Conn.D, K), or structure model index (SMI, L) 28 days post-ACLT. N=8 mice.

Supplemental Tables

Supplemental Table 1. Subject demographics

	**Mean ± SD**	**Range**	**Median**
Combined			
N	23		
Age	18.5 ± 4.7	15 - 35	17
BMI	25.7 ± 3.5	21.4 - 34.0	25.1
Days post-injury	46 ± 47	9 - 152	22
Males			
N	9		
Age	16.8 ± 1.0	16 - 18	16
BMI	27.2 ± 3.9	22.2 - 34.0	25.8
Days post-injury	33 ± 33	9 - 116	21
Females			
N	14		
Age	19.6 ± 5.8	15 - 35	17
BMI	24.7 ± 3.0	21.4 - 30.6	23.8
Days post-injury	55 ± 54	11 - 152	30

Supplemental Table 2. Fiber counts for cross-sectional area analysis

Human		Mean ± SD
	Healthy	959 ± 510
	ACL-injured	809 ± 529
	1wk post-ACLR	879 ± 396
	4mo post-ACLR	1083 ± 590
**Mouse**		**Mean ± SD**
**7d**		
	PLA Ab Control	2115 ± 770
	PLA Ab ACLT	1385 ± 215
	GDF8 Ab Control	1750 ± 279
	GDF8 Ab ACLT	1581 ± 363
	GDF8KO Control	2296 ± 80
	GDF8KO ACLT	2404 ± 102
**14d**		
	PLA Ab Control	1331 ± 140
	PLA Ab ACLT	1325 ± 145
	GDF8 Ab Control	1457 ± 196
	GDF8 Ab ACLT	1476 ± 162
	GDF8KO Control	2356 ± 299
	GDF8KO ACLT	2258 ± 364
**28d**		
	PLA Ab Control	1350 ± 127
	PLA Ab ACLT	1364 ± 166
	GDF8 Ab Control	1310 ± 123
	GDF8 Ab ACLT	1496 ± 195
	GDF8KO Control	2234 ± 138
	GDF8KO ACLT	2153 ± 147
